# Hemispheric Asymmetry in the Genetic Overlap between Schizophrenia and White Matter Microstructure

**DOI:** 10.34133/cbsystems.0451

**Published:** 2026-01-09

**Authors:** Yujie Zhang, Mengge Liu, Shaoying Wang, Wanwan Zhang, Haoyang Dong, Qian Qian, Yue Wu, Qian Wu, Jinglei Xu, Ying Zhai, Haolin Wang, Jingchun Liu, Yuxuan Tian, Qi Luo, Xinxing Li, Lining Guo, Fengtan Li, Feng Liu

**Affiliations:** ^1^ Department of Radiology and Tianjin Key Laboratory of Functional Imaging, Tianjin Medical University General Hospital, Tianjin 300052, China.; ^2^ School of Medical Imaging, Tianjin Medical University, Tianjin 300070, China.

## Abstract

White matter microstructure, essential for neural communication, is genetically influenced and often disrupted in schizophrenia. Large-scale genome-wide association studies have identified over 200 genome-wide significant loci for schizophrenia, yet the extent to which schizophrenia shares genetic architecture with white matter microstructure—particularly across multidimensional diffusion tensor imaging (DTI) metrics and hemispheric distinctions—remains incompletely understood. Here, we employed the conditional/conjunctional false discovery rate (cond/conjFDR) approach to investigate the genetic overlap between schizophrenia and white matter microstructure. These analyses utilized large-scale genome-wide association datasets for schizophrenia (*N*_case_ = 53,386, *N*_control_ = 77,258) and the microstructure of 48 white matter tracts (*N* = 33,224), derived from individuals of European ancestry. White matter integrity was assessed using fractional anisotropy (FA), mean diffusivity (MD), and 3 eigenvalues. Additionally, we performed comprehensive functional and validation analyses for the shared loci. We identified 435 shared loci, including 154 loci exclusive to 3 eigenvalues. Hemisphere-specific analysis of white matter tracts revealed lateralized patterns, with 25.5% to 34.4% of loci being left-specific and 23.9% to 33.7% right-specific. Enrichment analysis highlighted the shared loci related to nervous system and central nervous system development, supporting their role in neurodevelopmental mechanisms. Validation analyses across diverse methods and datasets further confirmed the reliability of the shared loci. This study demonstrates a complex, shared genetic architecture between schizophrenia and white matter microstructure, highlighting hemispheric genetic asymmetry and the value of multidimensional DTI metrics in uncovering the genetic basis of structural brain abnormalities.

## Introduction

Schizophrenia is a complex and highly heterogeneous psychiatric disorder that affects approximately 1% of the global population [1]. Typically manifesting in adolescence or early adulthood, schizophrenia is characterized by a range of symptoms, including hallucinations, delusions, disorganized thinking, and cognitive impairments [2]. Despite considerable advances in understanding its underlying mechanisms, the exact etiology of schizophrenia remains elusive [3]. It has long been understood that genetic factors play a fundamental role in the occurrence and development of schizophrenia, with genome-wide association studies (GWASs) identifying hundreds of loci associated with the disorder, thereby expanding our knowledge of its genetic architecture [4].

White matter, composed of myelinated axons that facilitate communication between different brain regions, is a key factor in maintaining cognitive function and mental health [5,6]. The integrity of white matter is essential for efficient neural signal transmission, and disruptions in its microstructure have been implicated in various neuropsychiatric conditions, including schizophrenia [7,8]. One of the most effective tools for studying white matter microstructure in vivo is diffusion tensor imaging (DTI), a type of magnetic resonance imaging-based neuroimaging technique that measures the diffusion of water molecules in brain tissue [9]. DTI enables a detailed examination of the orientation and integrity of white matter fibers by quantifying metrics such as fractional anisotropy (FA) and mean diffusivity (MD) [10]. These metrics are derived from 3 eigenvalues—*λ*1, *λ*2, and *λ*3—which represent the principal directions of water diffusion within white matter tracts [11]. The parameter *λ*1 (axial diffusivity) reflects the diffusion of water parallel to white matter tracts and is associated with axonal integrity [12]. Radial diffusion, derived by averaging *λ*2 and *λ*3, describes the diffusion of water perpendicular to the tract and is related to myelin integrity [13]. Together, these eigenvalues provide more specific and complementary information on microstructural changes beyond FA and MD. In schizophrenia research, changes in white matter integrity have been observed across multiple tracts, with alterations in FA and MD, indicating compromised microstructure [14]. These changes are intricately linked to alterations in *λ*1, *λ*2, and *λ*3, where specific shifts in these eigenvalues may suggest different types of microstructural damage. Furthermore, white matter microstructure is highly heritable [15]; twin and family studies, along with GWAS, have shown that a considerable proportion of the variance in DTI metrics is influenced by genetic factors, highlighting the substantial genetic contribution to white matter integrity [16–18].

Growing evidence suggests a genetic overlap between schizophrenia and white matter microstructure. Previous studies have identified a weak genetic correlation between the disorder and white matter microstructure using linkage disequilibrium score regression (LDSC) [19,20], as well as a correlation between the polygenic risk score (PRS) for schizophrenia and alterations in white matter [21]. While these findings indicate genome-wide genetic overlap, they cannot identify specific genetic variants that contribute to both traits. Identifying these variants is vital to understanding the precise biological pathways involved and for developing targeted therapeutic strategies. Moreover, these methods like LDSC and PRS often underestimate the genetic overlap because they aggregate effects across the entire genome, leading to cancellation when different loci have effect directions that vary between traits, thereby overlooking more complex patterns of genetic sharing. In contrast, the conditional and conjunctional false discovery rate (cond/conjFDR) method enables the identification of shared genetic variants between the 2 traits, even when their effects differ across loci [22]. Leveraging this approach can thus reveal previously unrecognized genetic links, providing a more detailed understanding of the molecular mechanisms.

Building on this foundation, recent studies utilizing the cond/conjFDR approach have begun to identify specific genetic variants associated with both schizophrenia and alterations in white matter microstructure [23]. However, these studies primarily focused on FA and MD, overlooking the 3 eigenvalues of the diffusion tensor. These eigenvalues, which capture diffusivity along the principal axes, are fundamental to calculating FA and MD [24]. A direct examination of these eigenvalues could provide a more detailed understanding of specific microstructural changes in white matter, thereby enhancing insights into the pathophysiology of schizophrenia. Furthermore, Parker et al. [20] have analyzed average values across bilateral fiber tracts without examining hemisphere-specific differences in the shared genetic architecture between schizophrenia and white matter microstructure across the whole brain. Investigating these hemispheric differences could reveal whether genetic influences on white matter microstructure are uniformly distributed or show distinct patterns between hemispheres, shedding light on the role of lateralization in schizophrenia.

**Fig. 1.**
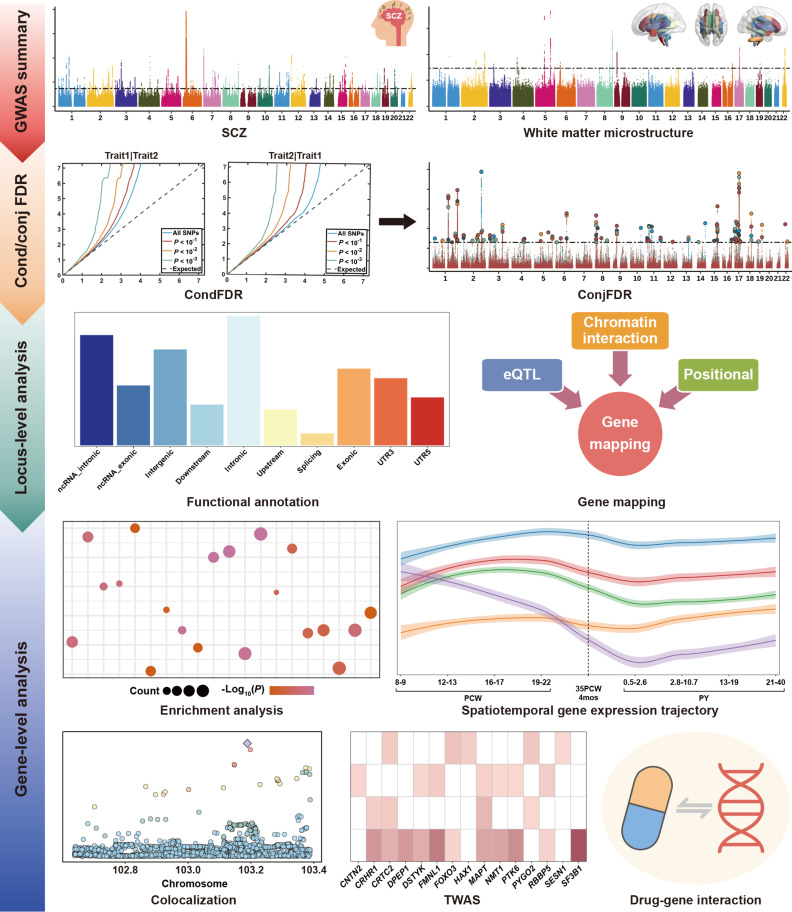
Flowchart of the study design and analysis. Based on genome-wide association study (GWAS) summary statistics for schizophrenia and 5 DTI parameters of white matter, we conducted conditional and conjunctional false discovery rate (cond/conjFDR) analyses to identify genomic loci associated with schizophrenia and white matter microstructure, as well as loci shared between them. For the identified loci, locus-level analyses were performed, including functional annotation and gene mapping. Finally, gene-level analyses encompassed the following: enrichment analysis, spatiotemporal gene expression trajectory analysis, colocalization analysis, transcriptome-wide association study (TWAS), and drug–gene interaction analysis. eQTL, expression quantitative trait locus; ncRNA, noncoding RNA; PCW, postconceptional weeks; PY, postnatal years; SCZ, schizophrenia; UTR3, 3′ untranslated region; UTR5, 5′ untranslated region.

Given the substantial heritability of both schizophrenia and white matter microstructure and the accumulating evidence for hemispheric lateralization in brain structure and function [25], we hypothesized (a) that a substantial proportion of genetic loci would be shared between these 2 traits, and (b) that a considerable proportion of the shared loci would show hemispheric specificity, reflecting potential lateralized genetic influences on white matter organization. To test these hypotheses, we applied the cond/conjFDR method using the largest available GWAS datasets for schizophrenia (*N*_case_ = 53,386, *N*_control_ = 77,258) [4] and for the microstructure of 48 hemisphere-specific white matter tracts (*N* = 33,224) [26] from individuals of European ancestry. Our comprehensive analysis incorporated key DTI metrics such as FA and MD, as well as the 3 eigenvalues of the diffusion tensor, to identify genetic loci shared between schizophrenia and white matter microstructure and assess their potential hemispheric specificity. The analytic framework of our study is illustrated in Fig. 1.

## Methods

### Data sources

The GWAS statistics for schizophrenia were obtained from the Psychiatric Genomics Consortium (PGC), which included 74,776 cases and 101,023 controls in the primary analysis [4]. For this study, we selected a subset of individuals of European ancestry, resulting in a dataset of 53,386 cases and 77,258 controls. White matter microstructure data, including summary statistics for 5 DTI parameters, were sourced from the UK Biobank and cover 33,224 individuals of European ancestry [26]. The DTI parameters analyzed—FA, MD, and the 3 eigenvalues (*λ*1, *λ*2, and *λ*3)—were derived from 48 white matter tracts defined by the JHU ICBM-DTI-81 white matter labels atlas [27,28]. Descriptions and abbreviations of the phenotypes are provided in Table S1, with further details of the GWAS dataset available in the corresponding publications [4,26]. Given the potential confounding due to population stratification in multi-ancestry data and variations in sample sizes across populations, we chose to use GWAS summary statistics from individuals of European ancestry to enhance the validity and interpretability of our results. Supplementary Methods provides an overview of the quality control procedures and key characteristics of the GWAS summary statistics on schizophrenia and white matter microstructure. All GWAS datasets used in this study were approved by the relevant ethics committees of the original studies, and informed consent was obtained from all participants.

### Cond/conjFDR analysis

Using an empirical Bayesian framework, the condFDR analysis enhances the detection of genetic variants by incorporating information from related phenotypes, thereby increasing the power to identify true associations [22]. Based on the strength of the association with the secondary phenotype, the condFDR analysis reorders the test statistics and recalculates the relationships between genetic variants—i.e., single-nucleotide polymorphisms (SNPs)—and the primary phenotype [29]. Additionally, conditional quantile–quantile (*Q*–*Q*) plots were constructed to visually depict SNP enrichment across traits by displaying the *P* value distributions for the primary phenotype across all SNPs or those associated with the secondary phenotype at varying significance levels (*P* < 0.1, *P* < 0.01, and *P* < 0.001). Regions with complex linkage disequilibrium (LD), including the major histocompatibility complex (MHC) region (chr6:25,119,106-33,854,733) and the 8p23.1 region (chr8:7,200,000-12,500,000) [30], were excluded from the analysis.

To explore genetic variants shared between schizophrenia and white matter microstructure, we conducted cond/conjFDR analysis. Specifically, 2 condFDR analyses were performed: one with schizophrenia as the primary phenotype and a specific DTI parameter as the secondary, and then with the roles reversed. This dual approach generated 2 condFDR values for each schizophrenia–white matter pair, with the conjFDR defined as the maximum of these values, allowing us to identify shared genetic variants between schizophrenia and the specific DTI parameters. Consistent with previous studies [31–33], the significance thresholds were set at 0.01 for condFDR and 0.05 for conjFDR analyses. A thorough explanation of the cond/conjFDR method can be found in Supplementary Methods.

### Genomic loci identification

Independent genomic loci were defined using the sumstats.py script (https://github.com/precimed/python_convert). Specifically, independent significant SNPs were identified as those with LD *r*^2^ < 0.6 with each other and meeting the thresholds of condFDR < 0.01 or conjFDR < 0.05 in the respective analyses. Among these SNPs, lead SNPs were defined as those with LD *r*^2^ < 0.1 with any other independent significant SNP. Genomic locus boundaries were determined by including candidate SNPs in LD *r*^2^ ≥ 0.6 with at least one independent significant SNP. Loci separated by less than 250 kb were merged into a single locus, with the SNP showing the smallest FDR value within each merged locus assigned as the lead SNP. In the MHC region and 8p23.1—regions with complex LD—all loci were merged, and the SNP with the smallest FDR value was chosen as the lead SNP. Additionally, for each DTI parameter, overlapping loci identified across the 48 fiber tracts were combined into distinct loci, with the lead SNP having the lowest FDR value selected as the top lead SNP.

To identify novel genomic loci, we used the following 2 criteria. First, the locus must be at least 500 kb away from any significant locus (*P* < 1 × 10^−6^) identified in the original GWAS [4,26], which was defined using the same method as in the present study. Second, all candidate SNPs within the locus must be absent from the NHGRI-EBI GWAS Catalog (https://www.ebi.ac.uk/gwas/) [34]. All of these criteria must be met for a locus to be considered novel.

### Functional annotation and enrichment analysis

The FUMA online platform (https://fuma.ctglab.nl/) was used to functionally annotate candidate SNPs with conjFDR < 0.1 [35]. The annotation included Combined Annotation Dependent Depletion (CADD) scores, RegulomeDB scores, and 15-core chromatin states. CADD integrates information from 63 annotations into a single score, with values exceeding 12.37 indicating potential pathogenicity [36]. The RegulomeDB score incorporates diverse regulatory data to assess the regulatory function and mechanism of a given variant, with lower scores indicating greater regulatory potential [37]. The 15-core chromatin states were predicted by the ChromHMM model based on 5 chromatin markers, using 15 categories to represent chromatin accessibility and regulatory activity of genomic regions, ranging from active promoter states to a quiescent state [38,39]. Additionally, these candidate SNPs were mapped to protein-coding genes through 3 approaches: positional mapping, which links SNPs to genes within a 10-kb window; expression quantitative trait locus (eQTL) mapping, which aligns SNPs with genes based on brain tissue data from Genotype-Tissue Expression (GTEx) v8 [40]; and chromatin interaction mapping, which matches SNPs to genes using 4 chromatin interaction datasets, including those from adult and fetal cortex [41], as well as from the dorsolateral prefrontal cortex and hippocampus in the GSE87112 cell line [42]. After excluding mapped genes located in the MHC region and 8p23.1, we conducted Gene Ontology (GO) biological process functional enrichment analysis on protein-coding genes shared between schizophrenia and individual DTI parameters using g:Profiler (https://biit.cs.ut.ee/gprofiler/gost) [43]. Multiple comparisons in the enrichment analysis were corrected using Benjamini–Hochberg FDR (BH-FDR) approach with a significance threshold of 0.05. Furthermore, to investigate the tissue- and cell-type specificity of genes shared between schizophrenia and all white matter microstructural phenotypes, we employed the Web-based Cell-type Specific Enrichment Analysis of Genes (WebCSEA) tool [44].

### Lifespan spatiotemporal gene expression trajectory analysis

To determine the spatiotemporal brain expression trajectories of genes associated with both schizophrenia and each specific DTI parameter, we utilized the processed human messenger RNA sequencing (mRNA-seq) dataset from the PsychENCODE study [45]. The dataset included 607 tissue samples from 41 postmortem brains, spanning an age range from 8 postconceptional weeks (PCW) to 40 postnatal years (PY), with gene expression levels represented as reads per kilobase of transcript per million mapped reads (RPKM). Expression profiles were categorized into 9 developmental stages: window 1 (8 to 9 PCW), window 2 (12 to 13 PCW), window 3 (16 to 17 PCW), window 4 (19 to 22 PCW), window 5 (35 PCW to 4 months), window 6 (0.5 to 2.6 PY), window 7 (2.8 to 10.7 PY), window 8 (13 to 19 PY), and window 9 (21 to 40 PY). A log_2_ transformation was applied to the expression values by adding 1 to each sample’s expression value, followed by centering the values around the mean using the R function scale(center = TRUE, scale = FALSE) + 1 [46]. The expression levels of each brain tissue sample were estimated by averaging the RPKM values of genes uniquely shared by schizophrenia and each specific DTI parameter. The expression trajectories were fitted using a nonlinear locally estimated scatterplot smoothing (LOESS) regression line, and 95% confidence intervals were calculated.

### Colocalization analysis

To assess whether the loci identified by conjFDR and gene expression share the same causal variants, we performed colocalization analyses using the Bayesian method implemented in the R package coloc [47]. GWAS summary statistics for schizophrenia and each white matter DTI parameter were tested against cis-eQTL summary data derived from 2,865 brain cortical samples of 2,443 unrelated individuals of European ancestry [48]. For each genetic locus shared between schizophrenia and a given DTI phenotype, we evaluated whether the association signals for the 2 traits colocalized with brain eQTLs within the same region. Colocalization was considered to occur when the posterior probability of a shared causal variant (PP.H4) exceeded 0.8, using the default prior settings.

### Transcriptome-wide association analysis

We conducted a transcriptome-wide association study (TWAS) using S-PrediXcan [49,50] to assess whether the expression of genes mapped from shared loci is associated with schizophrenia and the corresponding white matter microstructural phenotypes. Based on GTEx v8 reference data [51], S-PrediXcan provides eQTL-based prediction models of gene expression in 13 brain tissues, including amygdala, anterior cingulate cortex (BA24), caudate (basal ganglia), cerebellar hemisphere, cerebellum, cortex, frontal cortex (BA9), hippocampus, hypothalamus, nucleus accumbens (basal ganglia), putamen (basal ganglia), spinal cord (cervical c-1), and substantia nigra. To integrate associations across tissues, we further applied S-MultiXcan [52] to test the joint effects of gene expression across all 13 brain tissues. Significant gene–trait associations were determined using BH-FDR correction (*P* < 0.05).

### Drug–gene interaction analysis

The Drug–Gene Interaction Database (DGIdb; https://dgidb.org) is a publicly accessible resource that integrates drug and gene data from multiple sources (e.g., ChemIDplus, ChEMBL, and DrugBank), encompassing over 10,000 genes, 20,000 drugs, and more than 70,000 interaction records [53]. DGIdb also provides an interaction score for each drug–gene interaction, calculated based on supporting publications and data sources, to indicate its reliability. For the genes shared between schizophrenia and white matter microstructure, we employed DGIdb to identify potential drug–gene interactions.

### Validation analysis

To validate the effectiveness and advantages of the cond/conjFDR method, as well as the loci shared between schizophrenia and white matter microstructure, we performed several validation steps.

First, we used LDSC to calculate the global genetic correlation between schizophrenia and white matter microstructure phenotypes [54,55], which allowed us to assess the effectiveness of the cond/conjFDR method and validate or refute previous LDSC results [19,20]. Specifically, we used precomputed LD scores from the 1000 Genomes Project European population data (https://doi.org/10.5281/zenodo.7768714), restricting the analyses to HapMap3 SNPs. To account for multiple testing and ensure statistical significance, a BH-FDR correction with a threshold of *P* < 0.05 was applied.

Second, we utilized the Local Analysis of [co]Variant Association (LAVA) method [56], an integrated framework designed for local genetic correlation analysis within specific genomic regions, to replicate the findings from the cond/conjFDR analysis [57]. Univariate tests were conducted to assess the local genetic signal of the loci identified by conjFDR in schizophrenia and their corresponding white matter traits. Only loci demonstrating a significant univariate signal for both phenotypes were carried forward to bivariate tests, which assessed the local genetic correlations between schizophrenia and these white matter traits. Both univariate and bivariate tests were adjusted for multiple comparisons using the BH-FDR method, with a significance threshold set at *P* < 0.05.

Third, we employed an independent schizophrenia dataset, consisting of an East Asian population with 22,778 cases and 35,362 controls [58], to assess the replication of shared genomic loci between schizophrenia and white matter traits. Specifically, we calculated the consistency of allelic effect directions between the discovery dataset used in this study and the independent GWAS dataset for the lead SNPs within the shared loci associated with schizophrenia and each white matter microstructure parameter across the 48 fiber tracts [59]. This approach quantified the number of lead SNPs available in the independent sample and those showing consistent effect directions across both datasets. To determine the statistical significance of this consistency, we performed an exact binomial test, with *P* values corrected using the BH-FDR method.

Finally, we compared our findings with those from a previously published study that identified shared genomic loci between schizophrenia and white matter FA [20]. Since the locus ranges were not provided in that study, we defined them as extending 250 kb upstream and downstream from the lead SNPs. A locus was considered replicable if it overlapped with the distinct loci identified in our study as being shared between schizophrenia and FA phenotypes.

## Results

### Shared loci between schizophrenia and white matter microstructure revealed by cond/conjFDR

Conditional *Q*–*Q* plots revealed SNP enrichment between schizophrenia and white matter microstructure, indicating polygenic overlap between these traits (Figs. S1 to S5). At a significance level of condFDR < 0.01, we identified 402, 395, 433, 396, and 391 distinct loci associated with schizophrenia when conditioned on FA, MD, *λ*1, *λ*2, and *λ*3, respectively (Tables S2 to S6). Conversely, 481, 405, 496, 447, and 421 distinct loci were found to be associated with FA, MD, *λ*1, *λ*2, and *λ*3 when conditioned on schizophrenia (Tables S7 to S11). Detailed results of the condFDR analysis, including genomic locations and functional annotations for each locus identified for schizophrenia and the 5 DTI parameters, are provided for each white matter tract in Tables S12 to S16 and S17 to S21.

**Fig. 2.**
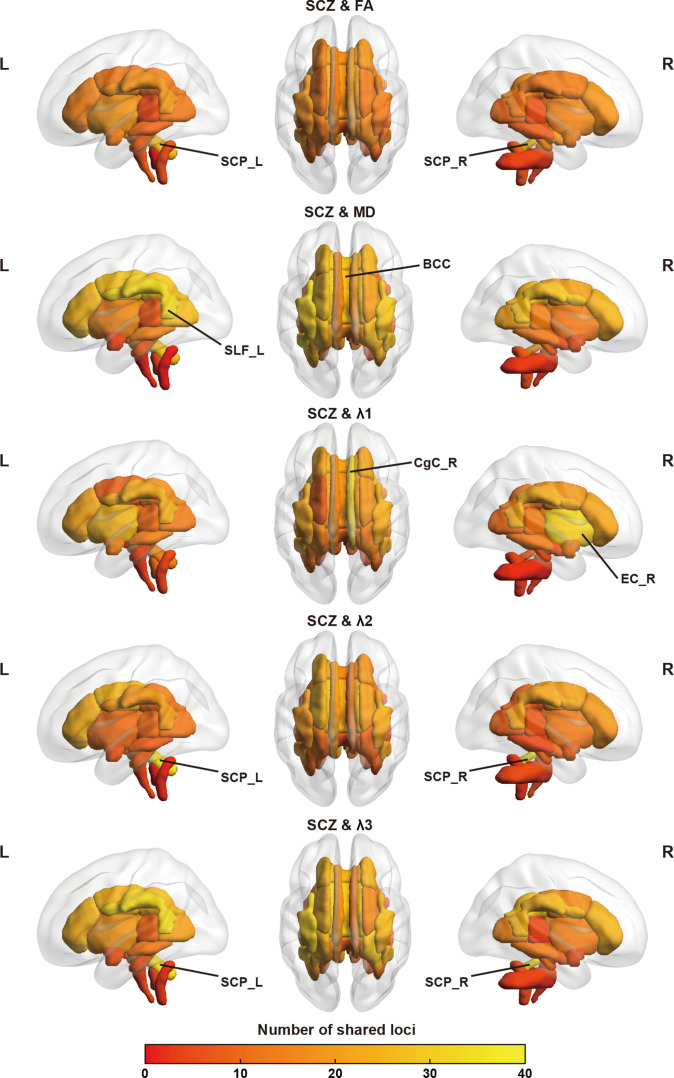
Brain maps showing the number of genomic loci shared between schizophrenia and 5 DTI parameters across 48 white matter tracts. The color bar represents the number of shared loci, shown as a gradient from yellow (low) to red (high). The white matter tracts with the 2 highest numbers of shared loci are labeled in the figure. BCC, body of corpus callosum; CgC, cingulum cingulate gyrus; EC, external capsule; FA, fractional anisotropy; L, left; MD, mean diffusivity; R, right; SCP, superior cerebellar peduncle; SCZ, schizophrenia; SLF, superior longitudinal fasciculus.

**Fig. 3.**
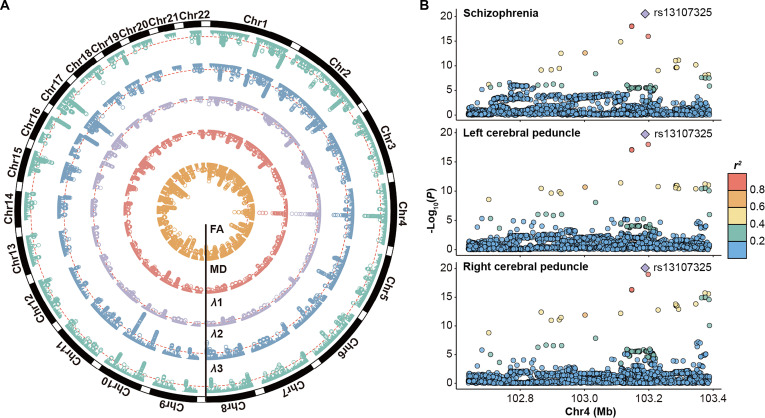
Results of loci shared between schizophrenia and white matter microstructure. (A) Circos Manhattan plot showing genomic loci shared between schizophrenia and 5 white matter DTI parameters identified through conjFDR analysis. From the inside to the outside, the layers represent FA, MD, *λ*1, *λ*2, and *λ*3. Each DTI parameter is shown in a distinct color. The conjFDR value for each SNP is determined by the minimum value across the 48 fiber tracts. The red dashed lines indicate the threshold of conjFDR < 0.05, and significant SNPs are highlighted as enlarged hollow circles. (B) LocusZoom plot of the genomic region chr4:102,638,777-103,388,441 showing regional association patterns for schizophrenia and *λ*1 in the bilateral CPs. The lead SNP rs13107325 is indicated. Chr, chromosome; FA, fractional anisotropy; MD, mean diffusivity.

**Fig. 4.**
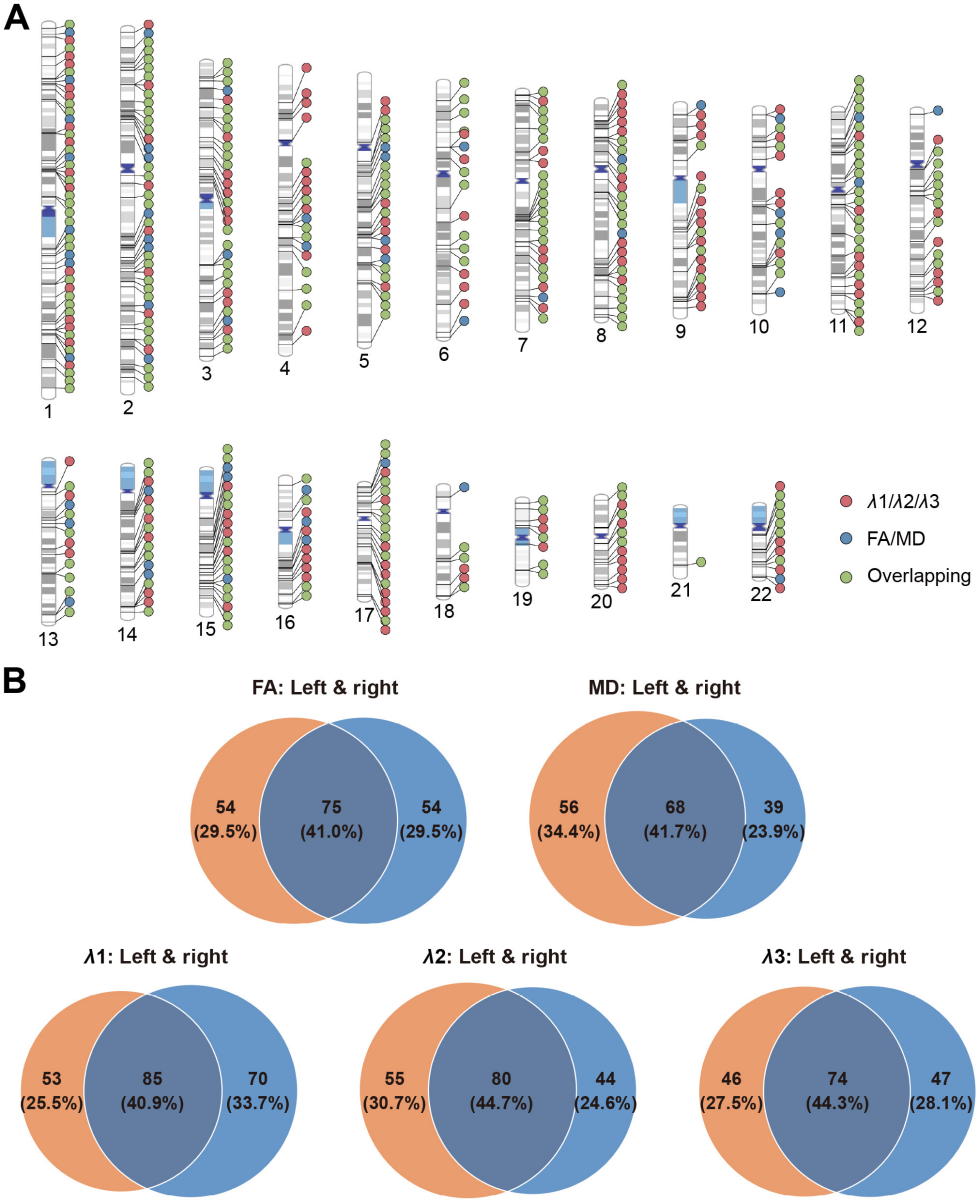
Chromosomal distribution and lateralized patterns of loci shared between schizophrenia and white matter microstructure. (A) Chromosomal distribution of distinct loci shared between schizophrenia and white matter microstructure identified by conjFDR analysis. Red represents loci specific to eigenvalues, blue represents loci specific to FA/MD, and green represents loci that are common to both the eigenvalues and FA/MD. Each dot corresponds to the top lead SNP of the distinct loci. (B) Venn diagram illustrating the lateralized patterns of loci shared between schizophrenia and the microstructure of 21 pairs of white matter tracts. The number and percentage of hemisphere-specific loci, along with shared loci, are shown. FA, fractional anisotropy; MD, mean diffusivity.

Building on these findings, and to test our first hypothesis, we next applied conjFDR to identify loci jointly associated with both traits. At a significance threshold of conjFDR < 0.05, we identified several loci shared between schizophrenia and each of the 5 DTI parameters across 48 white matter tracts (Fig. 2). The Manhattan plots for the 5 DTI parameters, showing the minimum conjFDR values across 48 tracts for all SNPs, are presented in Fig. 3A. The detailed results of the conjFDR analysis, including genomic locations and functional annotations for each shared locus, are provided in Tables S22 to S26 and Fig. S6. Among these loci, the most significant was located at chr4:102,638,777-103,388,441 and was shared between schizophrenia and *λ*1 in the bilateral cerebral peduncles (CPs; lead SNP rs13107325, left: conjFDR = 1.63 × 10^−14^; right: conjFDR = 1.72 × 10^−14^; Fig. 3B). After aggregating the loci across all 48 tracts (Tables S27 to S31), we identified 198, 188, 233, 200, and 182 distinct loci for FA, MD, *λ*1, *λ*2, and *λ*3, respectively. The distributions of the lead SNPs across chromosomes for each DTI parameter are shown in Figs. S7 to S11. By further combining loci across all 5 DTI parameters, we identified a total of 435 distinct loci, of which 49 were shared across all DTI parameters (Table S32). Additionally, 154 loci were exclusively shared between schizophrenia and at least one of the 3 eigenvalues (*λ*1, *λ*2, or *λ*3), but not with FA or MD (Fig. 4A). Of these 435 distinct loci, 112 were novel for schizophrenia, 130 were novel for white matter microstructure, and 27 for both (Table S32).

To test our second hypothesis, we examined whether the shared loci exhibited hemisphere-specific patterns, aiming to explore potential lateralized genetic influences on white matter microstructure. Of the 48 tracts, 42 (21 pairs) were present in both hemispheres, and our analysis focused on these 21 pairs. Specifically, we investigated whether these loci were left-specific, right-specific, or shared between the 2 hemispheres. The proportion of left-specific loci for FA, MD, *λ*1, *λ*2, and *λ*3 ranged from 25.5% to 34.4%, while right-specific loci ranged from 23.9% to 33.7% (Fig. 4B). The chromosomal distributions of left-specific, right-specific, and shared loci for the 5 DTI parameters are shown in Figs. S12 to S16. Furthermore, Fig. S17 depicts the proportions of left-specific, right-specific, and overlapping loci for each tract pair across the 5 DTI parameters. Notably, the left posterior thalamic radiation (PTR) and tapetum (TAP) consistently showed a higher proportion of shared loci than their right-sided counterparts. These findings support our second hypothesis that the loci shared by schizophrenia and distinct DTI parameters may exhibit hemispheric specificity.

### Functional annotation and enrichment analysis

Among the genomic loci shared between schizophrenia and the DTI parameters FA, MD, *λ*1, *λ*2, and *λ*3, a total of 33, 22, 29, 23, and 16 lead SNPs, respectively, were identified as pathogenic, with CADD scores exceeding 12.37 (Tables S22 to S26). For example, the SNP rs45510500, shared between schizophrenia and *λ*1 in the right superior longitudinal fasciculus, had the highest CADD score of 29.10 (Table S24). Moreover, the lead SNPs shared between schizophrenia and the 5 DTI parameters showed RegulomeDB scores of less than 3 for 6.80%, 7.65%, 5.49%, 8.05%, and 7.05%, respectively, suggesting a higher likelihood of regulatory function (Tables S22 to S26). Furthermore, the majority of these SNPs exhibited a minimum chromatin state of less than 8 (ranging from 90.44% to 92.73%), indicating that these shared loci are predominantly located in regions with open chromatin states (Tables S22 to S26).

**Fig. 5.**
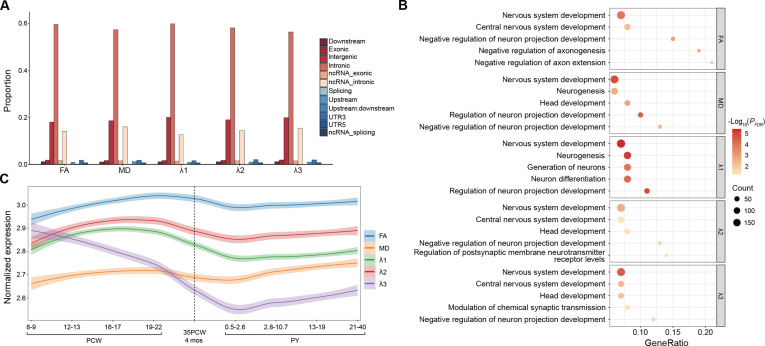
Functional annotation, enrichment analysis, and gene expression trajectory patterns of genes shared between schizophrenia and white matter microstructure. (A) Distribution of functional categories for candidate SNPs with conjFDR < 0.1 in loci shared between schizophrenia and white matter microstructure. (B) Bubble plot showing the significant biological processes enriched in protein-coding genes shared between schizophrenia and each DTI parameter. The *x* axis represents the ratio of genes enriched in the pathway to the total number of genes in the gene set, while the *y* axis represents the GO terms. The bubble size corresponds to the number of enriched genes, and the color represents the −log_10_-transformed BH-FDR adjusted *P* values. (C) Development trajectories of genes uniquely shared between schizophrenia and each specific DTI parameters of white matter. The *x* axis represents post-conception days, divided into 9 developmental stages. The *y* axis represents the whole-brain expression levels. A nonlinear LOESS regression fits the curve, and the shaded area represents the 95% confidence interval. Vertical dotted lines indicate births. FA, fractional anisotropy; FDR, false discovery rate; MD, mean diffusivity; ncRNA, noncoding RNA; PCW, postconceptional weeks; PY, postnatal years; UTR3, 3ʹ untranslated region; UTR5, 5ʹ untranslated region.

Annotation of candidate SNPs with conjFDR < 0.1 within the loci shared between schizophrenia and FA, MD, *λ*1, *λ*2, and *λ*3 revealed that the majority were intronic variants, with percentages of 59.55%, 57.35%, 59.85%, 58.09%, and 56.33%, respectively (Fig. 5A). Using 3 mapping approaches, these candidate SNPs were mapped to genes, identifying 1,289, 1,177, 1,335, 1,336, and 1,171 distinct protein-coding genes for FA, MD, *λ*1, *λ*2, and *λ*3, respectively (Tables S33 to S37). Of these, 499 genes were shared between schizophrenia and all 5 DTI parameters, while FA, MD, *λ*1, *λ*2, and *λ*3 uniquely shared 109, 62, 293, 82, and 23 genes with schizophrenia, respectively (Fig. S18). Detailed information on these candidate SNPs is provided in Tables S38 to S42. Enrichment analyses of the mapped genes revealed 132, 178, 293, 148, and 96 significantly enriched biological pathways for FA, MD, *λ*1, *λ*2, and *λ*3, respectively (Fig. 5B and Tables S43 to S47). Notably, the genes associated with all 5 DTI parameters were enriched in biological processes related to nervous system development, negative regulation of neuron projection development, and central nervous system development. Genes shared between schizophrenia and FA, MD, or *λ*1 were enriched in synapse organization, whereas those shared with *λ*2 and *λ*3 showed no such enrichment. In addition, cell-type and tissue-specific enrichment analysis was conducted using WebCSEA for 2,189 unique genes shared between schizophrenia and white matter microstructure. As illustrated in Fig. S19, the strongest enrichment was observed for erythroid progenitors of bone marrow (*P* = 6.09 × 10^−80^). Additional enriched cell types included monocyte (*P* = 1.99 × 10^−78^) and the radial glial cell (*P* = 1.99 × 10^−74^).

### Spatiotemporal gene expression trajectory across lifespan

The lifespan spatiotemporal gene expression trajectory analysis revealed dynamic changes in the expression of genes uniquely shared between schizophrenia and white matter microstructure, as indicated by the distinct trajectories observed across the 5 DTI parameters (Fig. 5C). Among these, genes associated with FA, MD, *λ*1, and *λ*2 displayed a relatively consistent temporal pattern, with a notable peak observed during the post-gestation period (19 to 22 PCW), followed by a gradual decline that continued into early childhood. The lowest expression levels were observed between 0.5 and 2.6 PY. After this decline, gene expression trajectories stabilized during later childhood and adolescence (approximately 2.8 to 19 PY) and remained relatively steady into adulthood, up to 40 PY. In contrast, genes associated with *λ*3 exhibited a distinct trajectory, with expression beginning to decline during the early-gestation period (8 to 9 PCW), reaching its lowest point between 0.5 and 2.6 PY, before gradually increasing through adulthood (up to 40 PY).

### Colocalization analysis

We performed a colocalization analysis to determine whether the loci shared between schizophrenia and white matter microstructure also serve as causal variants for both traits and gene expression. Across the 5 DTI parameters, 2,919 trait-level loci were identified (Tables S22 to S26), corresponding to 435 distinct nonoverlapping loci after merging overlaps (Table S32). Colocalization was conducted on the 2,919 trait-level loci, and evidence of colocalization with gene expression was observed for schizophrenia at 577 loci (Table S48). For white matter, colocalization was identified in 230 of 240 DTI phenotypes, involving 422 genomic loci. Notably, schizophrenia and 200 white matter phenotypes colocalized with the expression of the same genes at shared loci. For example, both schizophrenia and the FA of the left CP colocalized with the expression of the *PCLO* (ENSG00000186472) gene at chr7:82,503,409-82,616,835 (schizophrenia: PP.H4 = 0.828; left CP: PP.H4 = 0.819).

### TWAS analysis

**Fig. 6.**
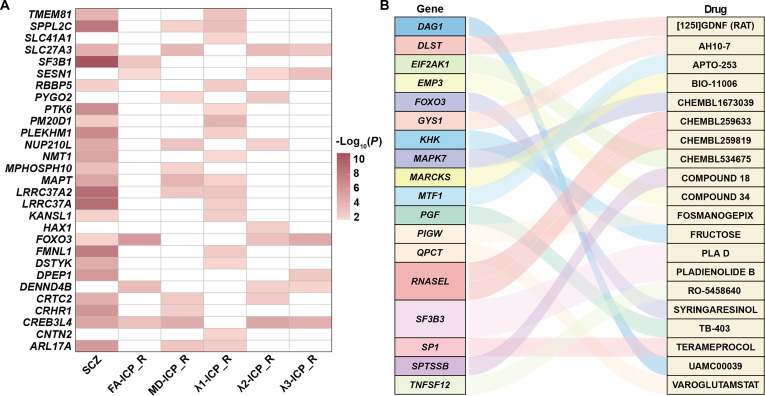
Brain expression and drug interaction profiles of genes shared between schizophrenia and white matter microstructure. (A) Gene–trait associations for schizophrenia and different DTI parameters (FA, MD, *λ*1, *λ*2, and *λ*3) of the right inferior cerebellar peduncle (ICP). The *x* axis represents traits, and the *y* axis lists gene names. The color of each rectangle corresponds to the −log_10_-transformed *P* value of the gene–trait association. (B) Predicted drug–gene interactions of the shared genes, with the top 20 interactions obtained from the DGIdb database. FA, fractional anisotropy; MD, mean diffusivity; SCZ, schizophrenia.

Using GWAS summary statistics for schizophrenia and white matter microstructural phenotypes together with eQTL data from 13 brain tissues, we conducted a TWAS analysis to examine whether the expression of genes identified by conjFDR was associated with both traits. After BH-FDR correction, we identified 7,348 significant gene–trait associations involving 880 unique genes across schizophrenia and 238 white matter microstructural phenotypes (Table S49). Notably, schizophrenia shared gene–trait associations with 237 of these phenotypes, with the only exception being *λ*3 of the right retrolenticular part of the internal capsule. Although the specific genes varied across phenotypes, the widespread overlap highlights robust transcriptomic convergence between schizophrenia and white matter microstructure. Representative associations were observed for *SPPL2C*, *MAPT*, *LRRC37A2*, and *ARL17A*, whose expression in the brain was significantly associated with schizophrenia as well as with MD and *λ*1 of the right inferior cerebellar peduncle (ICP) (Fig. 6A). Similarly, *SLC27A3* expression in the brain showed significant associations with MD, *λ*2, and *λ*3 of the right ICP, in addition to schizophrenia.

### Drug–gene interaction prediction

For the 2,189 unique genes shared between schizophrenia and white matter microstructure, we used DGIdb to predict potential drug–gene interactions. In total, 7,010 interactions were identified, involving 458 genes and 4,286 drugs (Table S50). Based on interaction scores provided by DGIdb, the top 20 drug–gene interactions are shown in Fig. 6B. Of these, BIO-11006 targeting *MARCKS* gene achieved the highest score, while PLA D and PLADIENOLIDE B were both predicted to target *SF3B3* gene.

### Validation analysis

To validate the effectiveness and advantages of the conjFDR method over the global genetic correlation approach, we performed LDSC analysis between schizophrenia and white matter microstructure phenotypes [54,55]. The analysis revealed that only one of the genetic correlations between schizophrenia and white matter microstructure phenotypes remained significant after FDR correction (Table S51): MD in the right fornix cres + stria terminalis (*r*_g_ = 0.136, SE = 0.036, BH-FDR *P* = 0.048). When considering nominal significance (*P* < 0.05, uncorrected), 77 correlations were identified across various white matter tracts (Fig. S20). Additionally, genetic correlations between FA and schizophrenia were predominantly negative, while correlations with MD, *λ*1, *λ*2, and *λ*3 tended to be positive.

To validate the loci shared between schizophrenia and white matter microstructure, we performed local genetic correlation analyses using LAVA [56]. Univariate analyses revealed that approximately 80% of the shared loci exhibited significant local genetic signals for both schizophrenia and white matter microstructure traits (Table S52). Based on these findings, we conducted bivariate analyses focusing on loci identified in the univariate analyses, over 60% of which showed significant local genetic correlations (Table S52). Among the loci with significant correlations, the most significant positive correlation was observed at chr8:8,088,230-10,283,602, where schizophrenia was positively correlated with FA in the left uncinate fasciculus (*r* = 0.57, BH-FDR *P* = 5.73 × 10^−4^). In contrast, the most significant negative correlation was found at chr4:102,638,777-103,388,441, where schizophrenia exhibited an inverse relationship with *λ*1 in the right CP (*r* = −1.00, BH-FDR *P* = 1.29 × 10^−7^). For further details, please refer to Table S52.

To evaluate the replication of shared genomic loci between schizophrenia and white matter traits, we further analyzed consistency in allelic effect directions using the independent dataset. For the shared loci between schizophrenia and each of the 5 DTI parameters across the 48 fiber tracts, the lead SNPs showed a high degree of consistency in allelic effect directions between the discovery and independent datasets, with agreement rates ranging from 76.47% to 78.99% across all parameters (Fig. S21). An exact binomial test was performed to assess the statistical significance of this consistency. After correction for multiple comparisons using the BH-FDR method, the results confirmed that the observed consistency in allelic directions was statistically significant for all 5 DTI parameters (Fig. S21), supporting the reliability of the shared loci between schizophrenia and white matter microstructure traits in an independent sample.

To determine whether our identified loci shared between schizophrenia and white matter FA could replicate the results from the previous study [20], we extended the lead SNPs identified by Parker et al. to include a 250-kb window upstream and downstream. This process resulted in the identification of 146 distinct loci across all FA traits. Of these, 102 loci overlapped with those found in our analysis (Table S53), providing evidence of the reproducibility of our results.

## Discussion

To the best of our knowledge, this study is the first to comprehensively explore the shared genetic architecture between schizophrenia and white matter microstructure, incorporating all 5 key DTI metrics—FA, MD, and the 3 eigenvalues (*λ*1, *λ*2, and *λ*3). We initially hypothesized (a) that schizophrenia and white matter microstructure would share a substantial proportion of genetic loci; and (b) that a considerable proportion of these shared loci would show hemispheric specificity, reflecting potential lateralized genetic influences on white matter organization. Our findings strongly support these hypotheses: We identified 435 distinct loci jointly associated with both traits, and approximately one-quarter to one-third of these loci exhibited hemisphere-specific patterns. These results provide novel evidence of widespread genetic overlap and lateralized genetic effects, offering new insights into the neurobiological basis of schizophrenia.

The conditional *Q*–*Q* plots demonstrate genetic overlap between schizophrenia and each DTI parameter (Figs. S1 to S5), a finding further supported by conjFDR analyses, which identified loci shared between schizophrenia and 48 white matter microstructures (Tables S22 to S26). Notably, several genes located near these shared loci exhibit strong neurobiological relevance, including *KIAA1109*, *FGF12*, and *EPHA7*. *KIAA1109*, located on chromosome 4q27, shows enriched expression in the pituitary, cerebellum, and cerebellar hemispheres according to GTEx [60]. It is functionally linked to endocytosis and vesicular trafficking, essential for neuronal migration and embryonic development, and its disruption has been associated with neurologic malformations and severe developmental delay [61], suggesting involvement in cortico-cortical association pathways. *FGF12* is highly expressed in developing cortical neurons and regulates neuronal excitability by modulating voltage-gated sodium channels [62,63]. Recombinant humanized *FGF12* (*rhFGF12*) confers neuroprotective effects in vitro and ameliorates central nervous system injury in zebrafish models [64], supporting its therapeutic potential. By stabilizing neuronal firing and promoting synaptic plasticity, *FGF12* may influence long-range white matter connectivity and could serve as a pharmacogenomic marker for mood stabilizer response. *EPHA7*, a member of the ephrin receptor tyrosine kinase family, is prominently expressed in the developing diencephalon and anterior mesencephalon [65], as well as in the frontal and occipital cortex [66]. It regulates axon guidance, dendritic spine formation, and synaptic maturation [67]. Reduced *EPHA7* expression in mice has been associated with shifts in corticothalamic projections [68], suggesting a role in the development of thalamocortical connectivity. Although also linked to tumor apoptosis and proliferation [69], its predominant neurodevelopment role highlights its relevance to white matter organization. Together, these loci offer insights into molecular mechanisms linking schizophrenia with white matter microstructure. Analogously, the observed functional convergence at the genetic level conceptually echoes principles described in neural decoding frameworks, where multilevel signal integration enables precise mapping between neural circuits and behavioral phenotypes [70].

By integrating all loci shared between schizophrenia and white matter microstructure, we identified 435 distinct loci, including 154 unique to the eigenvalue-based metrics (*λ*1, *λ*2, and *λ*3), suggesting that these metrics capture additional aspects of genetic overlap beyond FA and MD. A substantial proportion of these loci exhibited hemispheric specificity, with 25.5% to 34.4% left-specific and 23.9% to 33.7% right-specific (Fig. 4B; based on 21 bilateral tract pairs from the 48 tracts). These results provide empirical support for our hypothesis regarding hemispheric specificity and may reflect the intrinsic asymmetry of brain structure and function, as well as potential hemispheric differences in the genetic regulation of white matter integrity. At the tract level, the left PTR and TAP consistently harbored more shared loci than their right-sided counterparts across all DTI parameters (Fig. S17). This lateralized pattern likely reflects asymmetric developmental trajectories: PTR and TAP undergo protracted postnatal myelination [71] and may be shaped by hemispheric differences in developmental timing [72], potentially increasing the sensitivity of left-hemispheric tracts to genetic effects. Functionally, PTR relays sensory and motor information [73], and TAP mediates interhemispheric hippocampal communication relevant to memory and emotional regulation [74]—functions commonly affected in schizophrenia. These findings align with prior evidence for lateralized genetic effects in psychiatric disorders [75–77] and suggest hemisphere-specific mechanisms shaping white matter architecture. The dynamic nature of white matter microstructure, potentially shaped by hemisphere-specific genetic effects, may reflect fluctuating network communication patterns within large-scale brain systems, a pattern supported by observations of dynamic functional connectivity across time windows in animal studies [78]. Nevertheless, macrostructural features such as tract volume or cross-sectional area could enhance the detectability of genetic associations in larger tracts and should be considered as potential confounders when interpreting the observed hemispheric asymmetry [79,80].

Enrichment analysis of genes shared between schizophrenia and various DTI parameters revealed key biological processes related to neurodevelopment. As demonstrated in Tables S43 to S47, the shared loci across the 5 DTI parameters were significantly enriched in neurodevelopmental processes. These included nervous system development, which encompasses the progression from the formation to the maturation of neural tissue; negative regulation of neuron projection development, involving mechanisms that restrain axonal and dendritic outgrowth and thereby influence neural connectivity; and central nervous system development, which is essential for the integration and coordination of neural functions [81]. Genes shared between schizophrenia and FA, MD, and *λ*1 were enriched in synapse organization, whereas genes shared with *λ*2 or *λ*3 did not show significant enrichment, suggesting a more complex genetic landscape stemming from the diverse roles these loci play across different biological pathways [82,83]. Synapse organization, which governs the formation, maintenance, and pruning of synapses, is critical for efficient neural communication [84]. Dysregulation of these processes has been implicated in abnormal circuit formation and altered plasticity [85,86], providing a biologically meaningful link between the genetic architecture shared by schizophrenia and white matter microstructure and the neurodevelopmental basis of the disorder.

The spatiotemporal gene expression trajectory analysis reveals considerable fluctuations in the expression of genes shared between schizophrenia and white matter microstructure, particularly during the prenatal period, with stabilization occurring after 0.5 to 2.6 PY (Fig. 5C). Although *λ*3 and other DTI parameters exhibit varying temporal dynamics, all show marked prenatal activity, suggesting that these genes play a critical role in early neurodevelopment. This aligns with our enrichment analyses, which point to their involvement in key brain development processes. The observed temporal patterns support the neurodevelopmental hypothesis of schizophrenia, underscoring the importance of prenatal brain development in the disorder’s etiology [87,88]. By pinpointing critical periods of gene activity, this study offers insights into the neurodevelopmental mechanisms underlying schizophrenia, suggesting potential windows for early intervention and a better understanding of how disruptions in these processes may contribute to the pathophysiology of this disorder. Future research combining genomic discoveries with high-resolution neurotechnologies, such as high-throughput microelectrode arrays [89] and high-density electroencephalogram [90], may allow direct testing of genetically informed hypotheses and advance a mechanistic understanding of white matter asymmetry in schizophrenia.

Colocalization analysis was conducted to assess whether shared loci between schizophrenia and white matter microstructure also serve as causal variants through gene expression. For example, both schizophrenia and the FA of the left CP colocalized with the expression of the *PCLO* gene (Table S48). *PCLO* is moderately expressed in the developing cerebral cortex and plays a crucial role in neuronal development and survival [91]. These findings highlight *PCLO* as a promising candidate gene linking schizophrenia to alterations in white matter microstructure. Using TWAS, we identified significant gene–trait associations shared between schizophrenia and white matter microstructure. In particular, the expression of *SPPL2C*, *MAPT*, *LRRC37A2*, and *ARL17A* was significantly associated with schizophrenia as well as with MD and *λ*1 of the right ICP. Among these, *MAPT* encodes the microtubule-associated protein tau, which is crucial for axonal microtubule stabilization, neuronal survival, and development [92]. These findings suggest that the shared genetic architecture between schizophrenia and white matter microstructure may reflect disruptions in neuronal pathways. The 2,189 unique genes shared between schizophrenia and white matter microstructure were analyzed using DGIdb, which identified 4,286 drugs predicted to interact with these genes (Table S50). Notably, BIO-11006 exhibited the highest interaction score with the *MARCKS* gene, while PLA D and PLADIENOLIDE B were both predicted to target the *SF3B3* gene. These results provide a valuable resource of potential therapeutic targets and candidate drugs, which may inform future efforts to modulate schizophrenia and white matter microstructural alterations.

The genetic correlation analysis revealed that only one of the correlations between schizophrenia and white matter microstructure remained significant after FDR correction (Table S51), consistent with previous reports of weak correlations [19,20]. The limitation of LDSC in aggregating effects across the entire genome may account for this finding, as opposing effects at different loci could cancel each other out. In contrast, the cond/conjFDR method offers several advantages, including the ability to detect shared genetic variants even when their effects are heterogeneous across the genome. By combining data from 2 GWASs, this method increased statistical power and allowed for the identification of loci shared between schizophrenia and white matter microstructure. Notably, several lead SNPs exhibited opposite directions of effect, further supporting the notion that LDSC’s inability to capture these complexities contributes to its weak correlation (Tables S22 to S26). Validation of the shared loci was conducted through other 3 analyses, confirming the robustness of our findings. Over 60% of the univariate significant loci demonstrated significant local genetic correlations (Table S52), and the allelic effect direction test showed a high level of agreement in effect directions (76.47% to 78.99%) between the discovery and independent datasets (Fig. S21). Moreover, 102 of the 146 loci identified by Parker et al. [20] were successfully replicated in our study, further supporting the reliability of our results (Table S53). These results demonstrate that our results are reliable, and highlight the superior power of the cond/conjFDR method in identifying shared genetic variants.

While condFDR improves the discovery power for individual traits by leveraging cross-trait enrichment, conjFDR is designed to identify loci jointly associated with both traits, thereby pinpointing shared genetic signals. Despite their complementary strengths, several methodological constraints should be considered when interpreting our findings. First, the performance of these approaches depends heavily on the statistical power of both GWAS datasets. Robust cross-trait enrichment only emerges when both traits have adequately powered GWAS; insufficient sample sizes or weak association signals can markedly reduce sensitivity and discovery yield, particularly for condFDR analyses [93]. Second, both methods rely exclusively on marginal additive SNP effects [94], which limits their ability to capture nonlinear genetic architectures or epistatic interactions that may underlie complex traits. Third, despite exclusion of well-known long-range LD regions such as the MHC and 8p23.1, residual long-range LD or subtle population stratification may still inflate test statistics, generating spurious enrichment patterns and false positives [95]. Finally, cond/conjFDR is primarily applicable to common autosomal variants and has limited capacity to detect rare variants or signals on the sex chromosomes, which are often excluded from GWAS [96,97]. These caveats highlight the importance of interpreting the identified loci within the assumptions of these methods.

Several limitations of this study should be acknowledged. First, our analyses were primarily based on European-ancestry GWAS data. Although the consistency of allelic effect directions was confirmed using additional data from East Asian cohorts, the extent to which these findings generalize to non-European populations remains uncertain and requires further validation. Second, the analyses were restricted to common variants located on autosomes, excluding rare variants and those on the sex chromosomes, which may also play a role in the shared genetic architecture of schizophrenia and white matter microstructure. Third, while this study focused on 5 widely used DTI parameters, other complementary white matter metrics were not examined. Incorporating additional neuroimaging markers, such as the regional vulnerability index [98], in future work may yield a more comprehensive understanding of white matter alterations. Fourth, DTI-derived measures are inherently constrained by their spatial resolution and may be influenced by preprocessing procedures (e.g., resampling), which could introduce measurement noise and affect the accuracy of white matter estimates. Applying higher-resolution imaging datasets alongside standardized and rigorously quality-controlled processing pipelines may help address these methodological challenges. Fifth, functional validation of the identified shared loci was not performed. In the future, experimental studies will be needed to establish causal relationships and to elucidate the biological mechanisms linking these genetic variants to white matter microstructure. Finally, environmental factors and gene–environment interactions were not examined, although environmental exposures have been shown to shape brain structure and function [99] and influence psychiatric risk through biological pathways [100].

## Conclusion

This study provides compelling evidence of a shared genetic basis between schizophrenia and white matter microstructure, highlighting the critical role of white matter integrity in the pathophysiology of schizophrenia. By utilizing the cond/conjFDR approach, we identified substantial genetic overlap, revealing potential biological pathways involved in neurodevelopment and central nervous system function. Notably, the inclusion of additional DTI metrics and the examination of hemisphere-specific loci offer a more comprehensive understanding of the genetic influences on white matter, capturing more specific alterations in both the left and right hemispheres. These findings underscore the complexity of the genetic underpinnings of schizophrenia and suggest that targeting these shared loci may offer new avenues for understanding the structural brain abnormalities and cognitive impairments associated with this disorder.

## Data Availability

The GWAS summary statistics for the 5 DTI parameters of white matter are accessible at https://open.win.ox.ac.uk/ukbiobank/big40/. Summary statistics for schizophrenia GWAS in European and East Asian populations are available at https://pgc.unc.edu/for-researchers/download-results/. The processed mRNA-seq data are available at http://development.psychencode.org/. The DGIdb database can be accessed at https://dgidb.org. This study utilizes publicly available software and code, including cond/conjFDR analysis (https://github.com/precimed/pleiofdr), g:Profiler (https://biit.cs.ut.ee/gprofiler), LAVA (https://github.com/josefin-werme/LAVA), LDSC (https://github.com/bulik/ldsc), and sumstats.py (https://github.com/precimed/python_convert). The genomic loci identified through the condFDR and conjFDR analyses, together with the full analysis scripts used in this study, are publicly available at https://github.com/zyjpp/SCZ_WM.
