# Supplementary material for: Hemispheric Asymmetry in the Genetic Overlap between Schizophrenia and White Matter Microstructure
Source: Cyborg Bionic Syst. 2026 Jan 9;7:0451. doi: 10.34133/cbsystems.0451 (PMC12783525; doi:10.34133/cbsystems.0451)
Supplement: Supplementary 1 — Figs. S1 to S21 Tables S1 to S54 [file cbsystems.0451.f1.zip › Supplementary Materials.docx]

**Supplementary Materials**

**Hemispheric Asymmetry in the Genetic Overlap Between Schizophrenia and White Matter Microstructure**

Yujie Zhang^1#^, Mengge Liu^1#^, Shaoying Wang^1#^, Wanwan Zhang^1#^, Haoyang Dong^1^, Qian Qian^1^, Yue Wu^1^, Qian Wu^1^, Jinglei Xu^1^, Ying Zhai^1^, Haolin Wang^1^, Jingchun Liu^1^, Yuxuan Tian^1,2^, Qi Luo^1,2^, Xinxing Li^1,2^, Lining Guo^1*^, Fengtan Li^1*^, Feng Liu^1*^

**Affiliations:**

^1^Department of Radiology and Tianjin Key Laboratory of Functional Imaging, Tianjin Medical University General Hospital, 300052 Tianjin, China

^2^School of Medical Imaging, Tianjin Medical University, Tianjin, 300070, China

^#^These authors contributed equally to this study

***Correspondence to:**

Feng Liu, Department of Radiology, Tianjin Medical University General Hospital, No. 154, Anshan Road, Heping District, Tianjin 300052, China.

E-mail: fengliu@tmu.edu.cn

Lining Guo, Department of Radiology, Tianjin Medical University General Hospital, No. 154, Anshan Road, Heping District, Tianjin 300052, China.

E-mail: liningguo@tmu.edu.cn

Fengtan Li, Department of Radiology, Tianjin Medical University General Hospital, No. 154, Anshan Road, Heping District, Tianjin 300052, China.

E-mail: left9999@sina.com

**Contents**

[Supplementary Methods 5](#_Toc208858338)

[Overview of genome-wide association studies (GWAS) summary statistics 5](#_Toc208858339)

[Schizophrenia 5](#_Toc208858340)

[White matter microstructural phenotypes 6](#_Toc208858341)

[Conditional and conjunctional false discovery rate (cond/conjFDR) 8](#_Toc208858342)

[CondFDR 8](#_Toc208858343)

[ConjFDR 9](#_Toc208858344)

[References 10](#_Toc208858345)

[Supplementary Figures 12](#_Toc208858346)

[Fig. S1. Polygenic overlap between schizophrenia and white matter fractional anisotropy. 24](#_Toc208858347)

[Fig. S2. Polygenic overlap between schizophrenia and white matter mean diffusivity. 37](#_Toc208858348)

[Fig. S3. Polygenic overlap between schizophrenia and white matter λ1. 50](#_Toc208858349)

[Fig. S4. Polygenic overlap between schizophrenia and white matter λ2. 63](#_Toc208858350)

[Fig. S5. Polygenic overlap between schizophrenia and white matter λ3. 76](#_Toc208858351)

[Fig. S6. The Manhattan plot of SNP shared between schizophrenia and white matter microstructure. 79](#_Toc208858352)

[Fig. S7. Ideogram of genomic genetic variants shared between schizophrenia and white matter fractional anisotropy. 80](#_Toc208858353)

[Fig. S8. Ideogram of genomic genetic variants shared between schizophrenia and white matter mean diffusivity. 81](#_Toc208858354)

[Fig. S9. Ideogram of genomic genetic variants shared between schizophrenia and white matter λ1. 82](#_Toc208858355)

[Fig. S10. Ideogram of genomic genetic variants shared between schizophrenia and white matter λ2. 83](#_Toc208858356)

[Fig. S11. Ideogram of genomic genetic variants shared between schizophrenia and white matter λ3. 84](#_Toc208858357)

[Fig. S12. Hemispheric distribution of distinct loci shared between schizophrenia and fractional anisotropy in 21 pairs of white matter tracts. 85](#_Toc208858358)

[Fig. S13. Hemispheric distribution of distinct loci shared between schizophrenia and mean diffusivity in 21 pairs of white matter tracts. 86](#_Toc208858359)

[Fig. S14. Hemispheric distribution of distinct loci shared between schizophrenia and λ1 in 21 pairs of white matter tracts. 87](#_Toc208858360)

[Fig. S15. Hemispheric distribution of distinct loci shared between schizophrenia and λ2 in 21 pairs of white matter tracts. 88](#_Toc208858361)

[Fig. S16. Hemispheric distribution of distinct loci shared between schizophrenia and λ3 in 21 pairs of white matter tracts. 89](#_Toc208858362)

[Fig. S17. Proportions of left-specific, right-specific, and overlapping loci across 21 tract pairs under different DTI parameters. 90](#_Toc208858363)

[Fig. S18. Venn diagram of overlapping genes shared between schizophrenia and five diffusion tensor imaging parameters. 92](#_Toc208858364)

[Fig. S19. Tissue- and cell-type specificity of genes shared between schizophrenia and white matter microstructure. 93](#_Toc208858365)

[Fig. S20. Genetic correlations between schizophrenia and white matter microstructure. 94](#_Toc208858366)

[Fig. S21. The allelic effect direction test of lead SNPs shared between schizophrenia and white matter microstructure. 95](#_Toc208858367)

# **Supplementary Methods**

## **Overview of genome-wide association studies (GWAS) summary statistics**

### **Schizophrenia**

The GWAS summary statistics for schizophrenia were derived from a study conducted by the Psychiatric Genomics Consortium (PGC) [[1](#_ENREF_1)]. The primary analysis included a total of 74,776 cases and 101,023 controls. The samples were obtained from multiple studies, including the European Network of National Schizophrenia Networks Studying Gene-Environment Interactions (EU-GEI), the Wellcome Trust Case-Control Consortium 2 (WTCCC2), and the Thematically Organised Psychosis Research (TOP). Among these subjects, approximately 80% of the individuals (53,386 cases and 77,258 controls) were of European ancestry, involving 75 datasets. The sample sizes, along with the number of males and females in each dataset, are provided in Table S54, with the proportion of females ranging from 0.082 to 0.732. Given the diversity of studies included, the diagnostic criteria for schizophrenia also varied, including: (1) clinical evaluations and hospital records; (2) meeting criteria for schizophrenia-related disorders in different versions of the Diagnostic and Statistical Manual of Mental Disorders (DSM), covering schizophrenia, schizoaffective disorder, schizophreniform disorder or chronic schizophrenia; (3) diagnoses according to the International Classification of Diseases, 10th Revision (ICD-10) criteria, with codes F20-F29 and F30-F33; and (4) diagnoses based on Research Diagnostic Criteria (RDC) using the Schedule for Affective Disorders and Schizophrenia Lifetime Version (SADS-L).

In accordance with the standards established by the PGC [[2](#_ENREF_2)], sample-level quality control (QC) procedures were applied to the core PGC cohorts. This included excluding individuals with a missingness rate ≥ 2% and performing a robust relatedness test. Pairs of individuals with a PI_HAT > 0.2 were identified using PLINK v1.9 [[3](#_ENREF_3)], and one individual from each pair was randomly removed, preferentially retaining cases and trio members over case-control participants. SNP-level QC included filtering out variants with an imputation information score (INFO) ≤ 0.8, minor allele frequency (MAF) ≤ 0.05, and significant deviations from Hardy-Weinberg equilibrium (HWE: *p* ≤ 1E-6 in controls or *p* ≤ 1E-10 in cases). Given the diverse origins of the core PGC cohorts, multiple measures were implemented to address population stratification. These included monitoring the genomic inflation factor (λ_GC_) within acceptable ranges and applying principal component analysis (PCA) filters to adjust for strongly stratified datasets. The first 20 PCs were included as covariates in association analyses, which were conducted using an additive logistic regression model. For detailed information on the QC procedures, please refer to the original PGC study [[1](#_ENREF_1)].

### **White matter microstructural phenotypes**

The GWAS summary statistics for white matter microstructure phenotypes were obtained from the UK Biobank, comprising 33,224 individuals of European ancestry [[4](#_ENREF_4)]. After preprocessing of the diffusion magnetic resonance imaging (dMRI) data, five parameters—fractional anisotropy (FA), mean diffusivity (MD), and the three eigenvalues (λ1, λ2, λ3)—were derived for 48 tracts using the tract-based spatial statistics (TBSS) method [[5](#_ENREF_5)]. Detailed information on the fiber tracts is available in the JHU ICBM-DTI-81 white matter label atlas [[6](#_ENREF_6),[7](#_ENREF_7)]. Prior to the GWAS analysis, sample-level QC was performed, including filtering out individuals with high heterozygosity or a missingness rate > 5%, identifying sex mismatches by comparing self-reported sex with genetically inferred sex from Affymetrix data, and excluding related individuals based on kinship coefficients calculated using KING [[8](#_ENREF_8)]. SNP-level QC criteria included excluding variants with MAF < 0.001, INFO < 0.3, or HWE -log_10_(*p*) > 7. Hypothesis testing was also conducted for batch effects, plate effects, HWE deviations, sex effects, and array effects, and any marker or marker-batch combination with a *p*-value < 1E-12 was considered to have failed QC and was subsequently set to missing [[9](#_ENREF_9)]. In the GWAS analysis, a comprehensive set of confounders was controlled for, including age, head size, sex, head motion during functional MRI, scanner table position, imaging center, scan-date-related slow drifts, and the first 40 population genetic PCs.

## **Conditional and conjunctional false discovery rate (cond/conjFDR)**

### **CondFDR**

The condFDR method utilizes GWAS summary statistics from both a primary and a secondary phenotype, applying an empirical Bayesian framework to identify single nucleotide polymorphisms (SNPs) associated with the primary phenotype given evidence from the secondary phenotype [[10](#_ENREF_10)]. The condFDR estimate for each SNP is obtained by computing the cumulative distribution function (CDF) of the primary phenotype conditional on the nominal *p*-values of the secondary phenotype.

For a specified *p*-value cutoff, the false discovery rate (FDR) is defined as:

$$\begin{aligned} FDR\left( p \right)=\frac{\pi_{0}F_{0}\left( p \right)}{F\left( p \right)}\#\left( 1 \right) \end{aligned}$$

where *π*_0_ is the proportion of null SNPs, $F_{0}$ is the null CDF, and $F$ is the CDF of all SNPs. Under the null hypothesis, *p*-values are assumed to be a *priori* independent and identically distributed. Thus, $F_{0}$ corresponds to the uniform CDF on [0, 1], which simplifies the formula to:

$$\begin{aligned} FDR\left( p \right)=\frac{\pi_{0}p}{F\left( p \right)}\#\left( 2 \right) \end{aligned}$$

The empirical CDF $F$ can be estimated as *q*  =  *Ν_p_* / *Ν*, where *Ν_p_* is the number of SNPs with *p*-values < *p*, and *N* is the total number of SNPs. Substituting $F$ with *q* and setting *π*_0_ = 1 yields:

$$\begin{aligned} FDR\left( p \right)=\frac{p}{q}\#\left( 3 \right) \end{aligned}$$

Therefore, the FDR is equivalent to the nominal *p*-value divided by its empirical quantile. This can be visualized in quantile-quantile (*Q-Q*) plots, where the relationship is expressed as:

$$\begin{aligned} {-log}_{10}\left( FDR\left( p \right) \right)={log}_{10}\left( q \right)-{log}_{10}\left( p \right)\#\left( 4 \right) \end{aligned}$$

Thus, the estimated FDR corresponds to the horizontal displacement of the *Q-Q* curve relative to the expected line *x* = *y*, with larger shifts indicating smaller FDR values.

### **ConjFDR**

The conjFDR method identifies SNPs associated with both primary and secondary phenotypes simultaneously. ConjFDR is defined as the posterior probability that a given SNP is null for both phenotypes, conditional on the observed *p*-values [[10](#_ENREF_10)]:

$$\begin{aligned} FDR\left( p_{1},p_{2} \right)=\frac{\pi_{0}\left( p_{1},p_{2} \right)F_{0}\left( p_{1},p_{2} \right)}{F\left( p_{1},p_{2} \right)}\#\left( 5 \right) \end{aligned}$$

where $\pi_{0}\left( p_{1},p_{2} \right)$ is the proportion of SNPs null for both traits, $F_{0}\left( p_{1},p_{2} \right)=p_{1}p_{2}$ is the joint null CDF, and $F\left( p_{1},p_{2} \right)$ is the empirical joint CDF. In practice, the condFDR procedure is applied twice, alternating between the primary and secondary phenotype, and the conjFDR is conservatively defined as the maximum of the two condFDR values:

$\begin{aligned} {FDR}_{phe1&phe2}=max\left\{ {FDR}_{phe1|phe2},{FDR}_{phe2|phe1} \right\}\#\left( 6 \right) \end{aligned}$

## **References**

[1] Trubetskoy V, Pardinas A F, Qi T, Panagiotaropoulou G, Awasthi S, Bigdeli T B, Bryois J, Chen C Y, Dennison C A, Hall L S, et al. Mapping genomic loci implicates genes and synaptic biology in schizophrenia. *Nature*. 2022;604(7906):502-508.

[2] Lam M, Awasthi S, Watson H J, Goldstein J, Panagiotaropoulou G, Trubetskoy V, Karlsson R, Frei O, Fan C C, De Witte W, et al. RICOPILI: Rapid Imputation for COnsortias PIpeLIne. *Bioinformatics*. 2020;36(3):930-933.

[3] Chang C C, Chow C C, Tellier L C, Vattikuti S, Purcell S M, Lee J J. Second-generation PLINK: rising to the challenge of larger and richer datasets. *Gigascience*. 2015;4:7.

[4] Smith S M, Douaud G, Chen W, Hanayik T, Alfaro-Almagro F, Sharp K, Elliott L T. An expanded set of genome-wide association studies of brain imaging phenotypes in UK Biobank. *Nat Neurosci*. 2021;24(5):737-745.

[5] Miller K L, Alfaro-Almagro F, Bangerter N K, Thomas D L, Yacoub E, Xu J, Bartsch A J, Jbabdi S, Sotiropoulos S N, Andersson J L, et al. Multimodal population brain imaging in the UK Biobank prospective epidemiological study. *Nat Neurosci*. 2016;19(11):1523-1536.

[6] Mori S, Oishi K, Jiang H, Jiang L, Li X, Akhter K, Hua K, Faria A V, Mahmood A, Woods R, et al. Stereotaxic white matter atlas based on diffusion tensor imaging in an ICBM template. *Neuroimage*. 2008;40(2):570-582.

[7] Wakana S, Jiang H, Nagae-Poetscher L M, van Zijl P C, Mori S. Fiber tract-based atlas of human white matter anatomy. *Radiology*. 2004;230(1):77-87.

[8] Manichaikul A, Mychaleckyj J C, Rich S S, Daly K, Sale M, Chen W M. Robust relationship inference in genome-wide association studies. *Bioinformatics*. 2010;26(22):2867-2873.

[9] Bycroft C, Freeman C, Petkova D, Band G, Elliott L T, Sharp K, Motyer A, Vukcevic D, Delaneau O, O'Connell J, et al. The UK Biobank resource with deep phenotyping and genomic data. *Nature*. 2018;562(7726):203-209.

[10] Andreassen O A, Thompson W K, Schork A J, Ripke S, Mattingsdal M, Kelsoe J R, Kendler K S, O'Donovan M C, Rujescu D, Werge T, et al. Improved detection of common variants associated with schizophrenia and bipolar disorder using pleiotropy-informed conditional false discovery rate. *PLoS Genet*. 2013;9(4):e1003455.

# **Supplementary Figures**


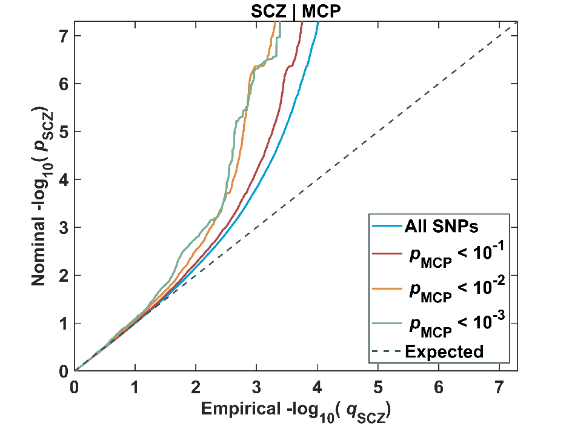

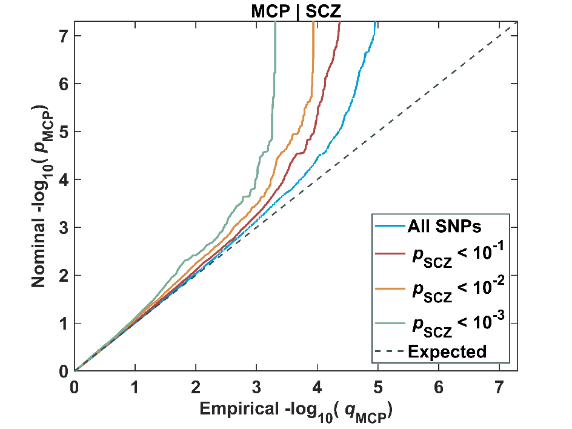

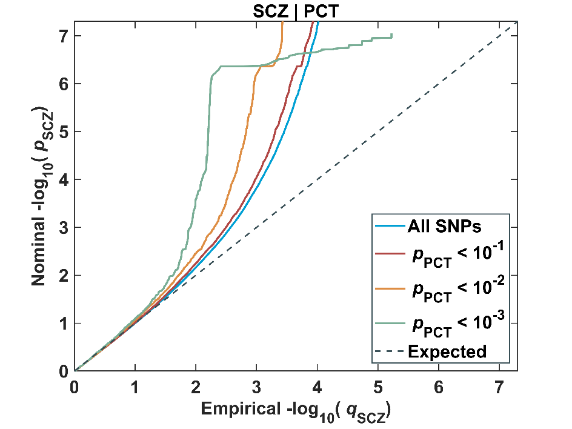

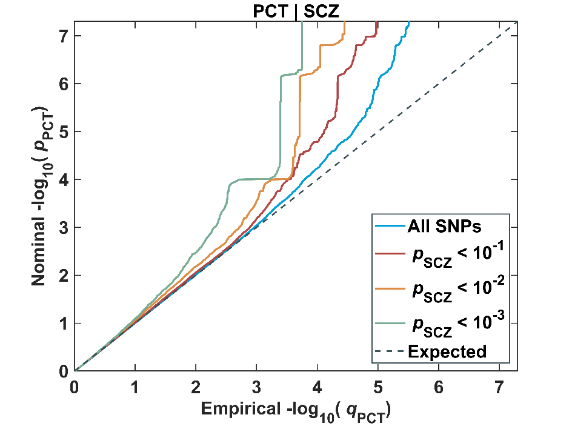

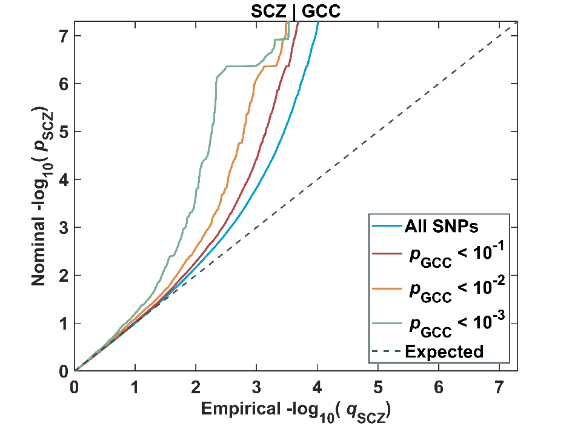

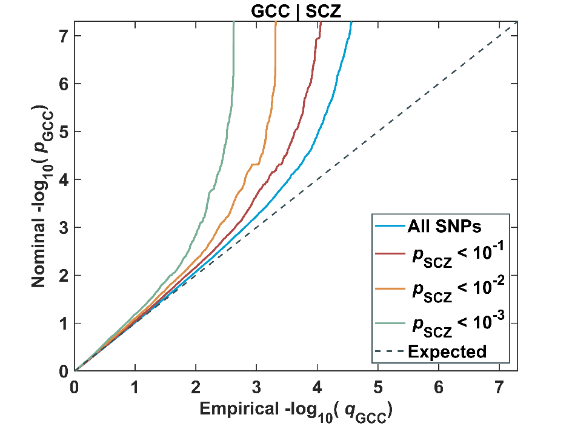

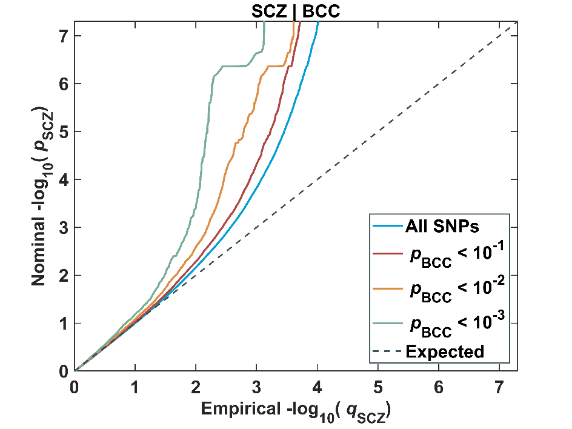

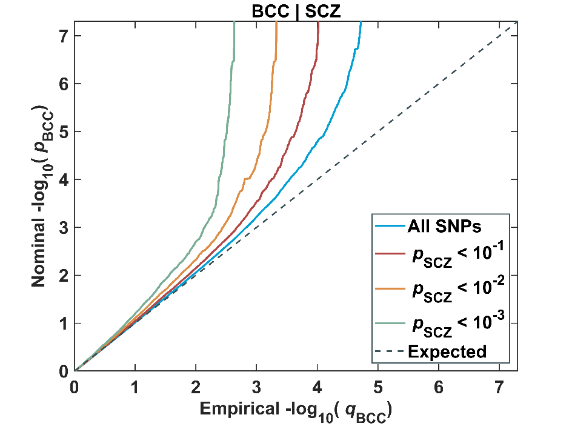

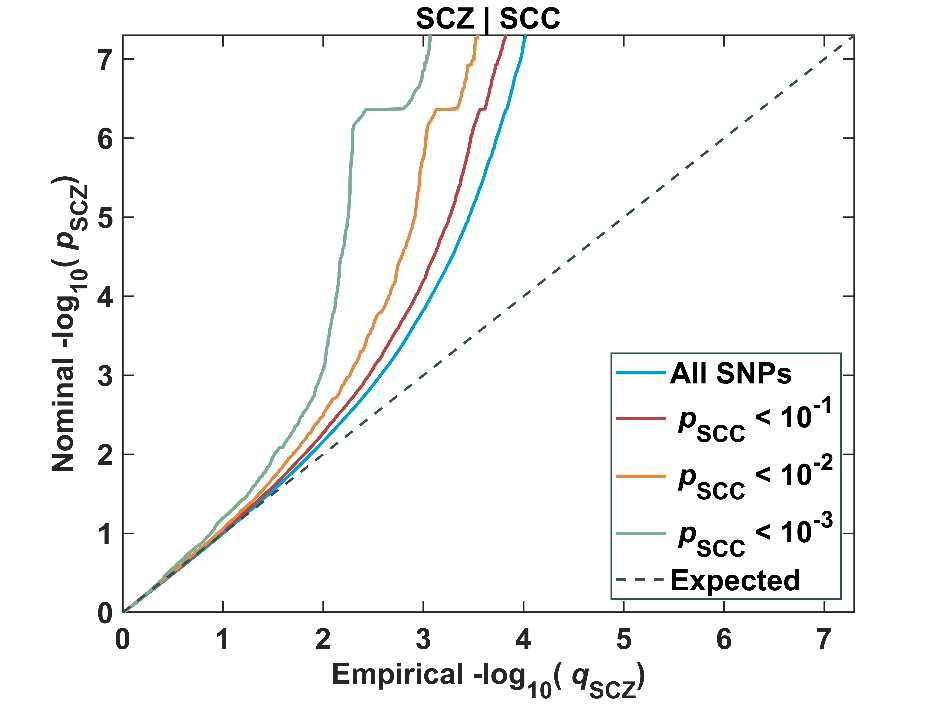

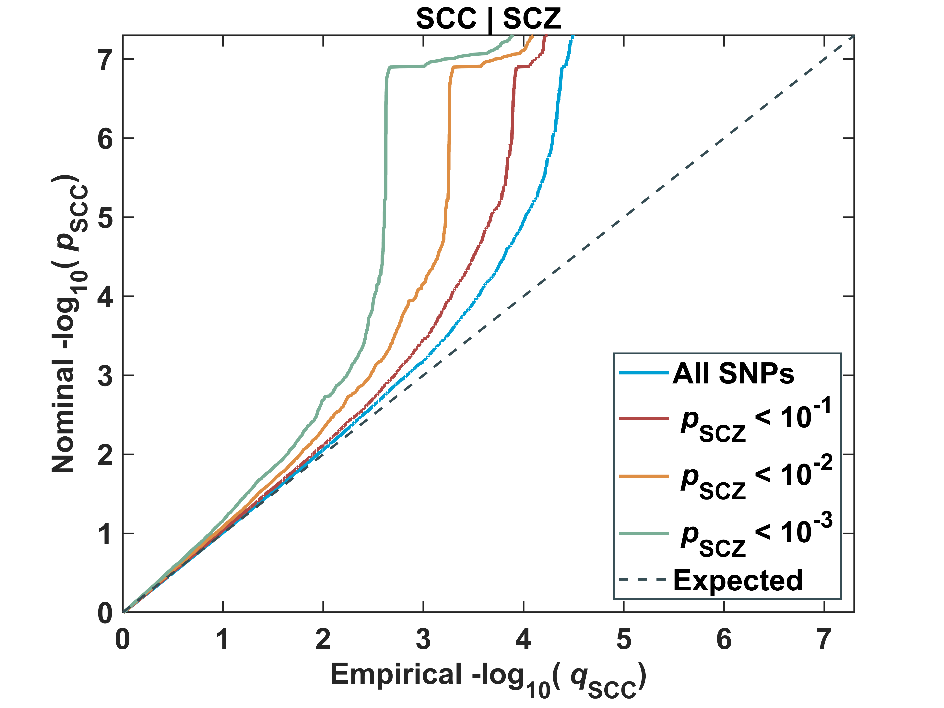

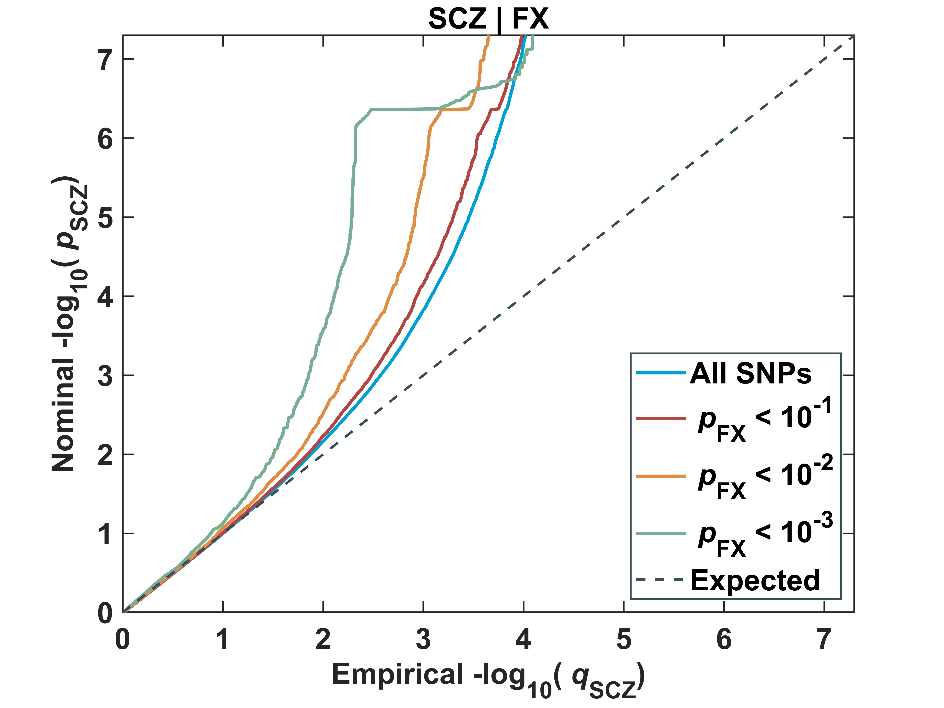

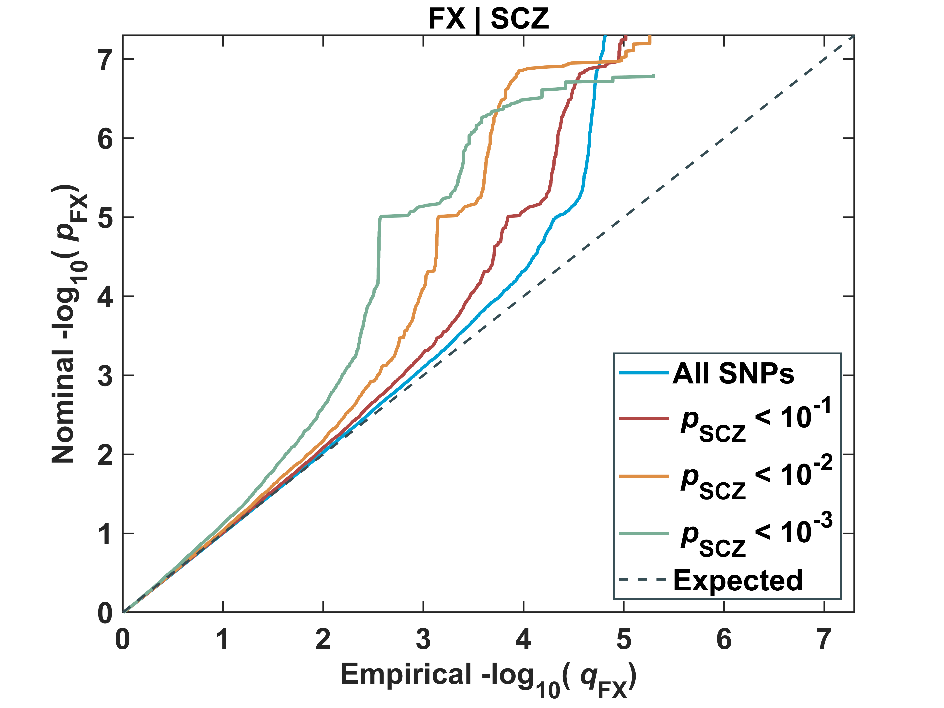

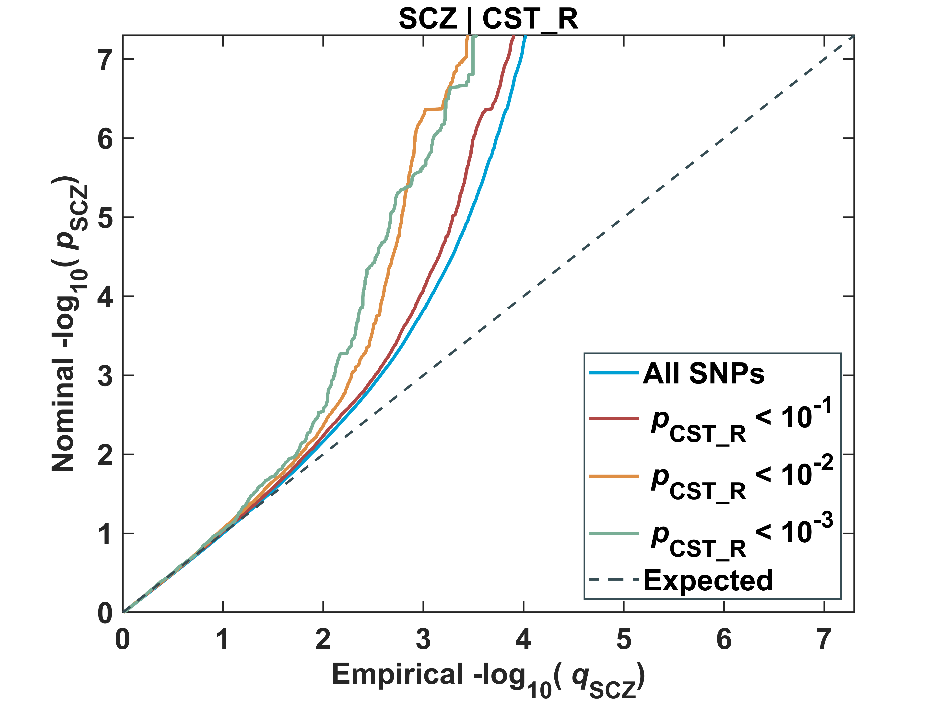

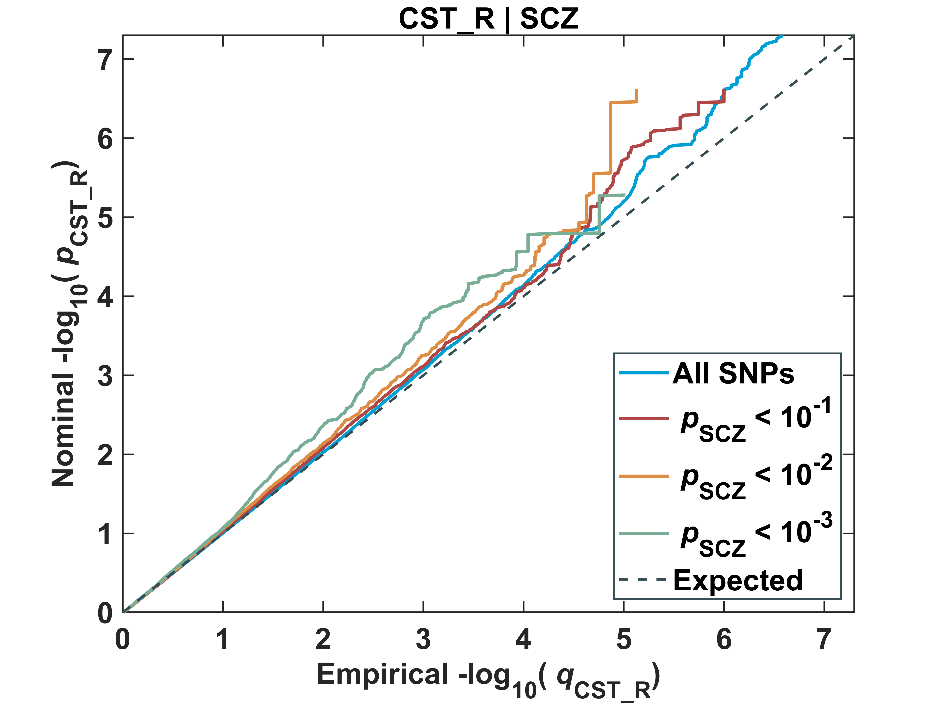

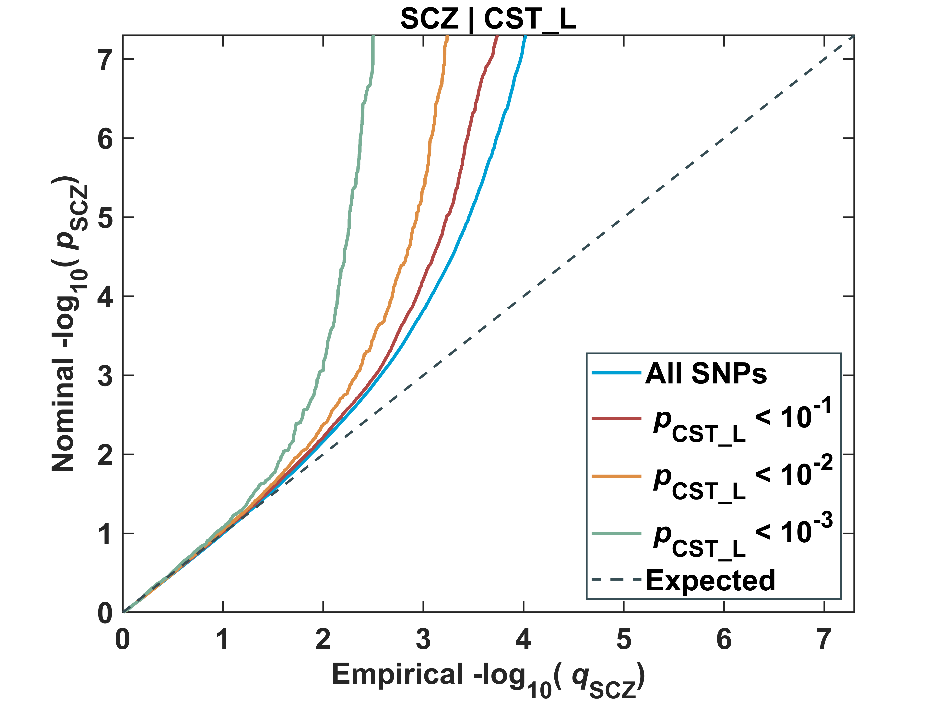

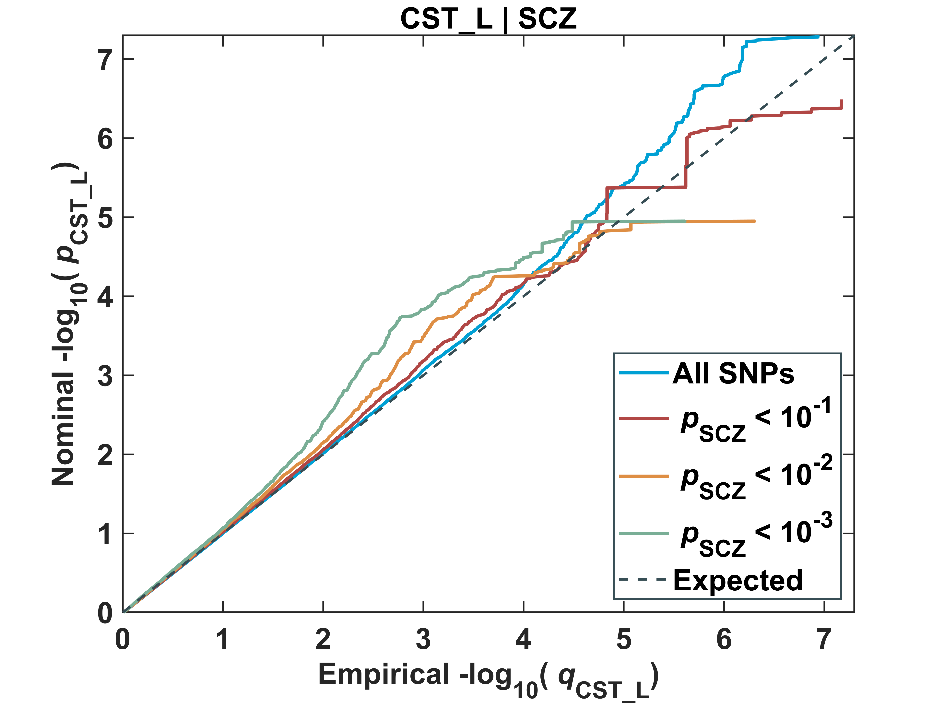

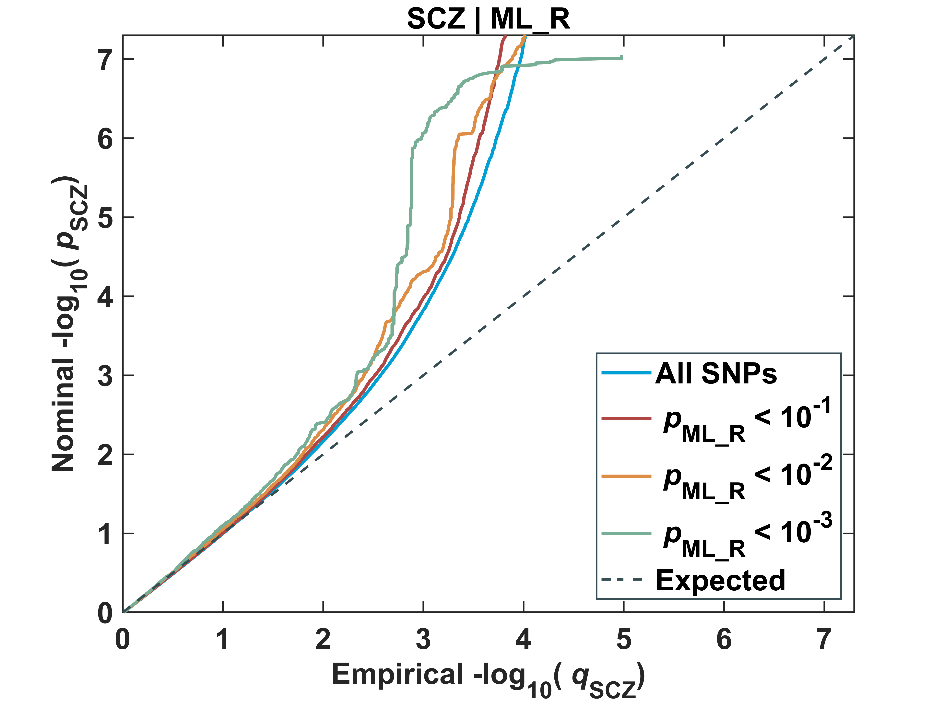

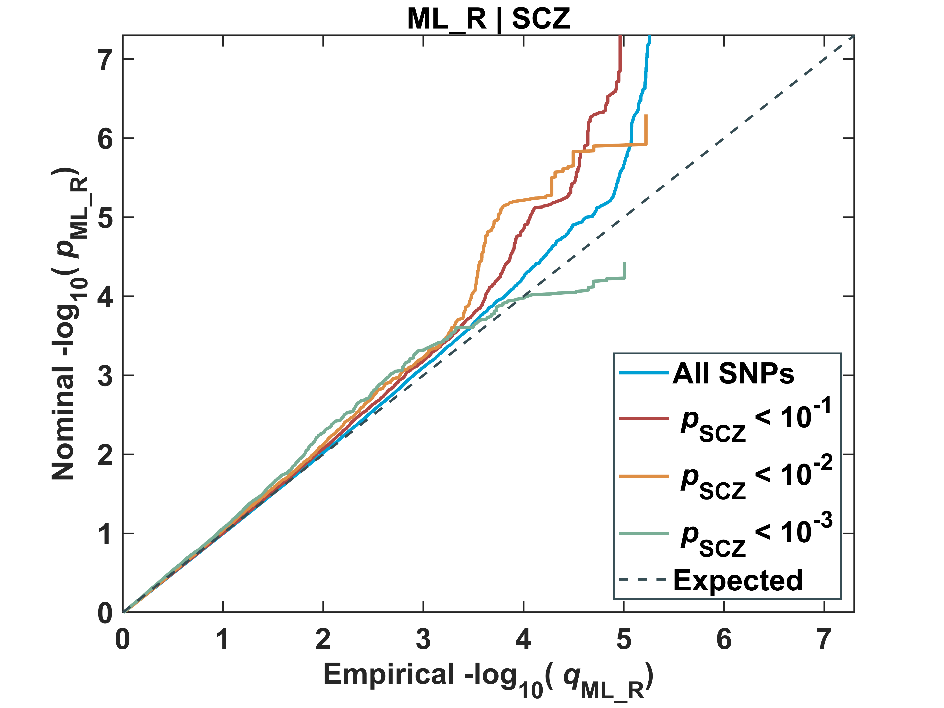

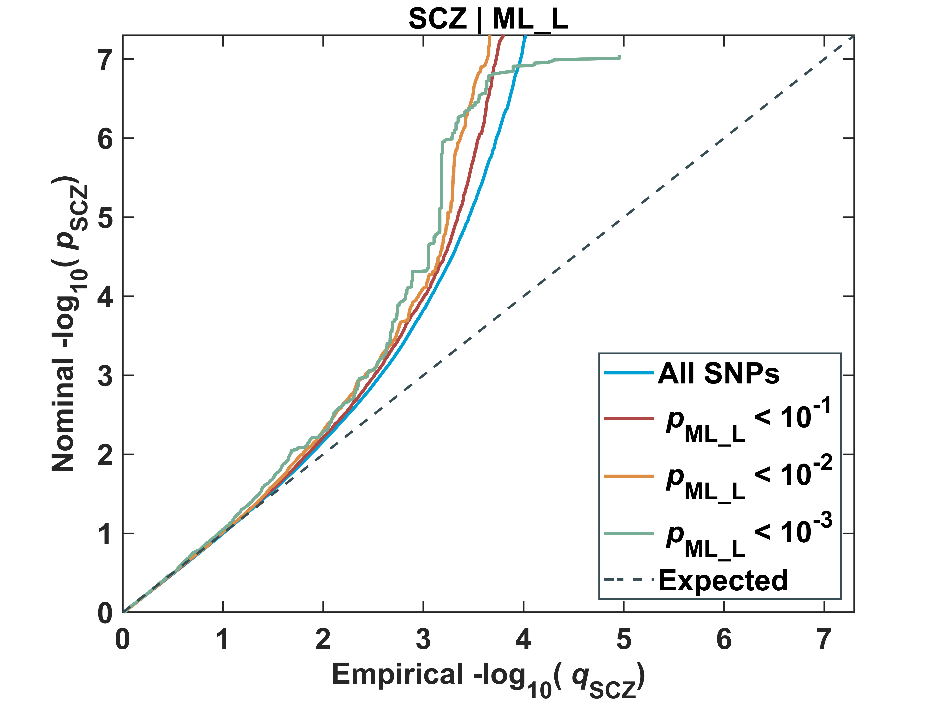

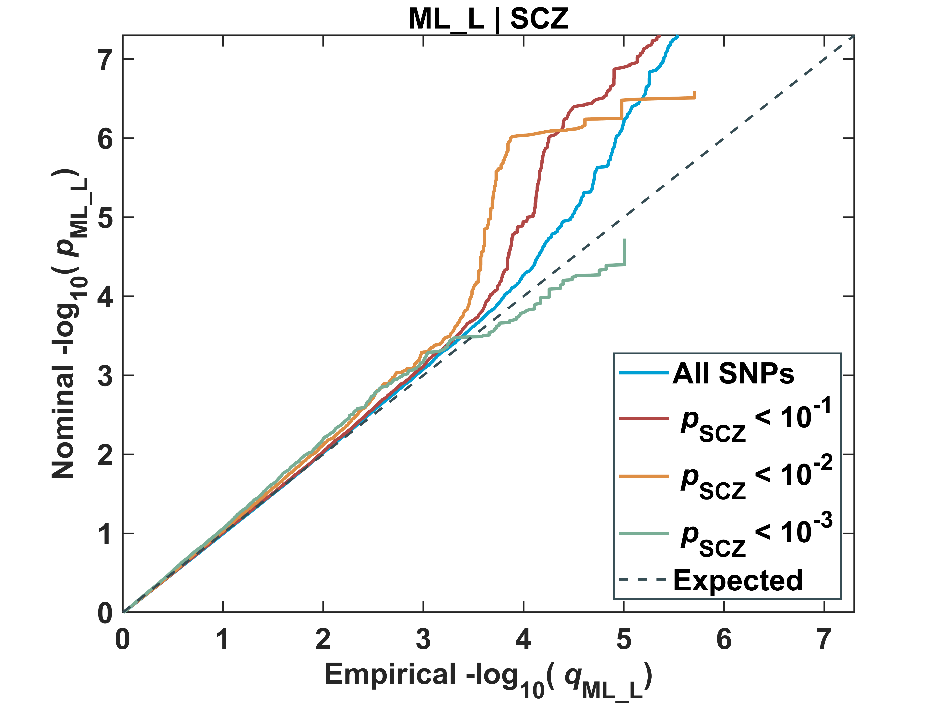

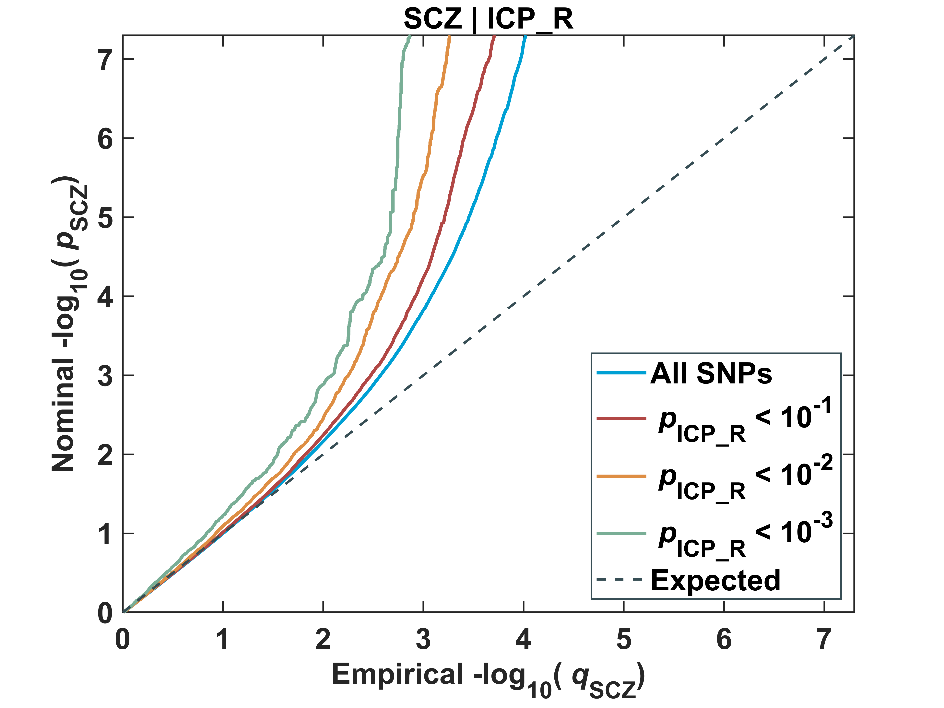

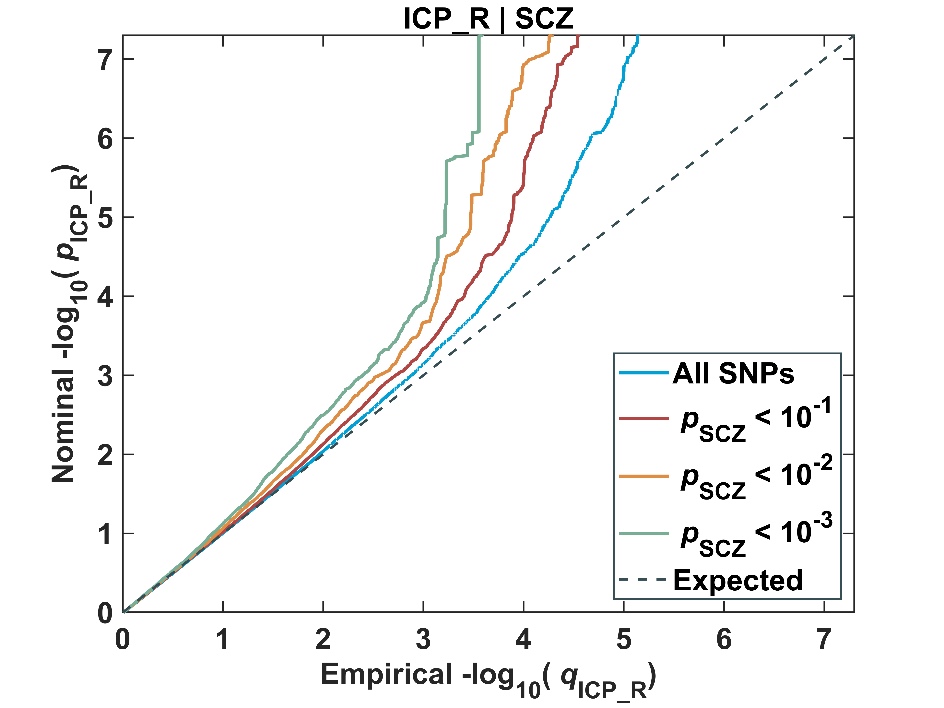

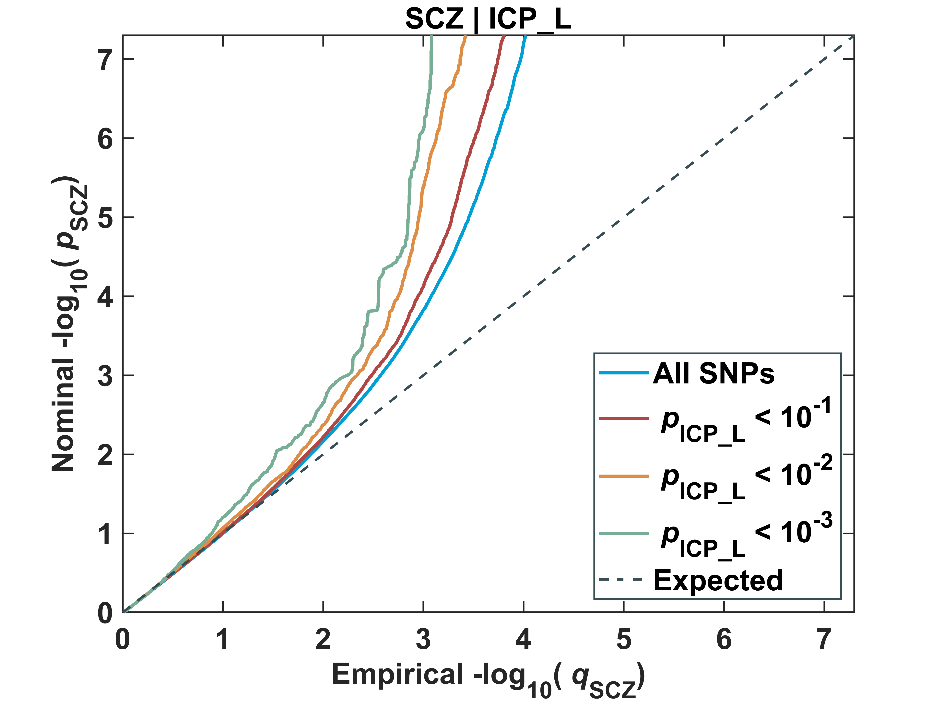

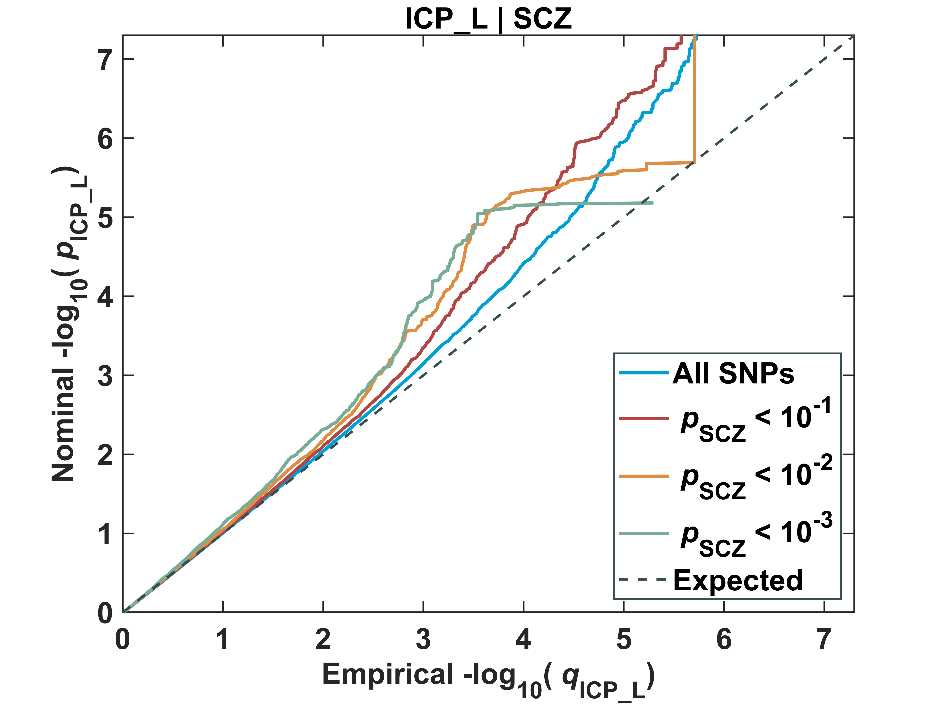

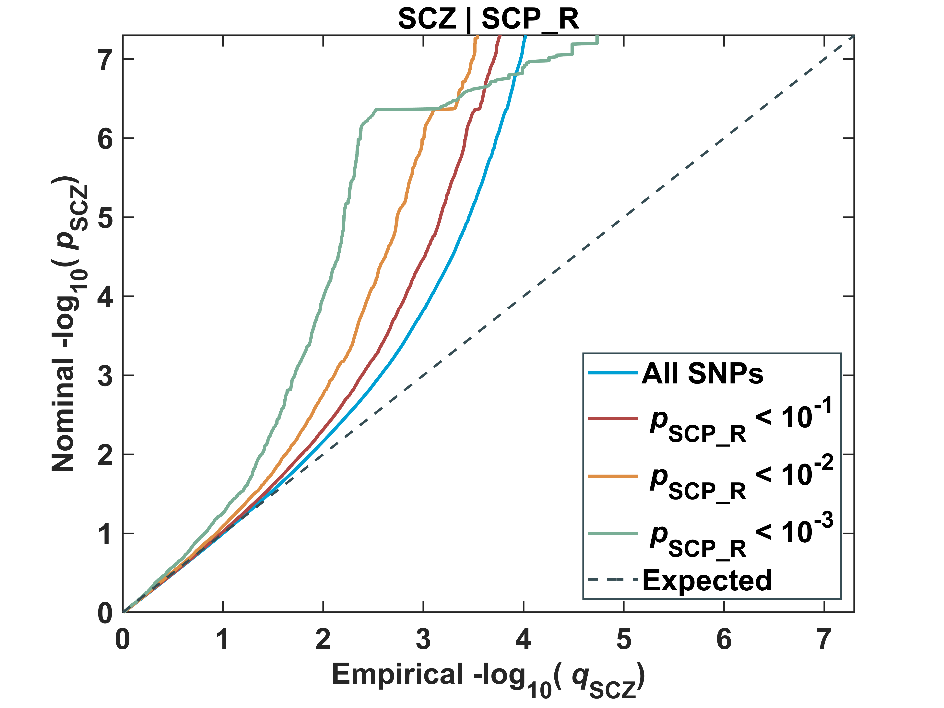

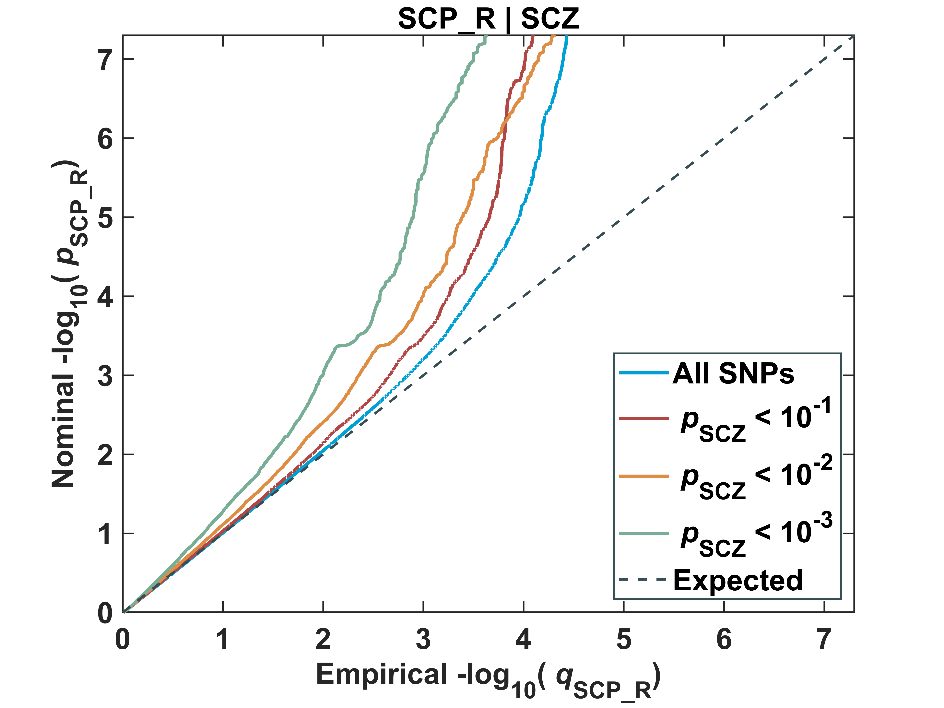

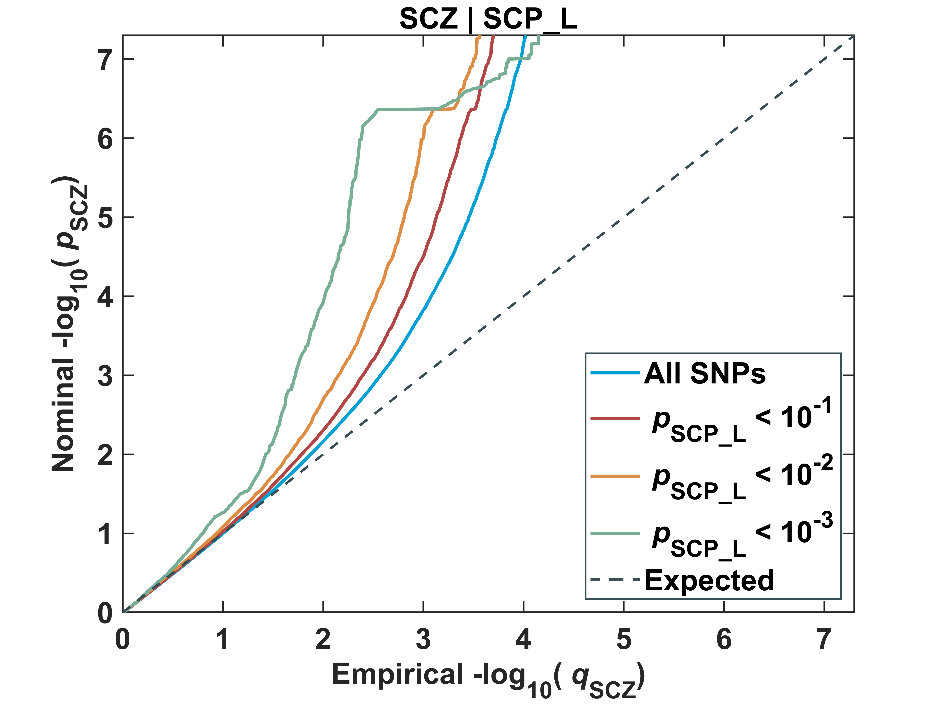

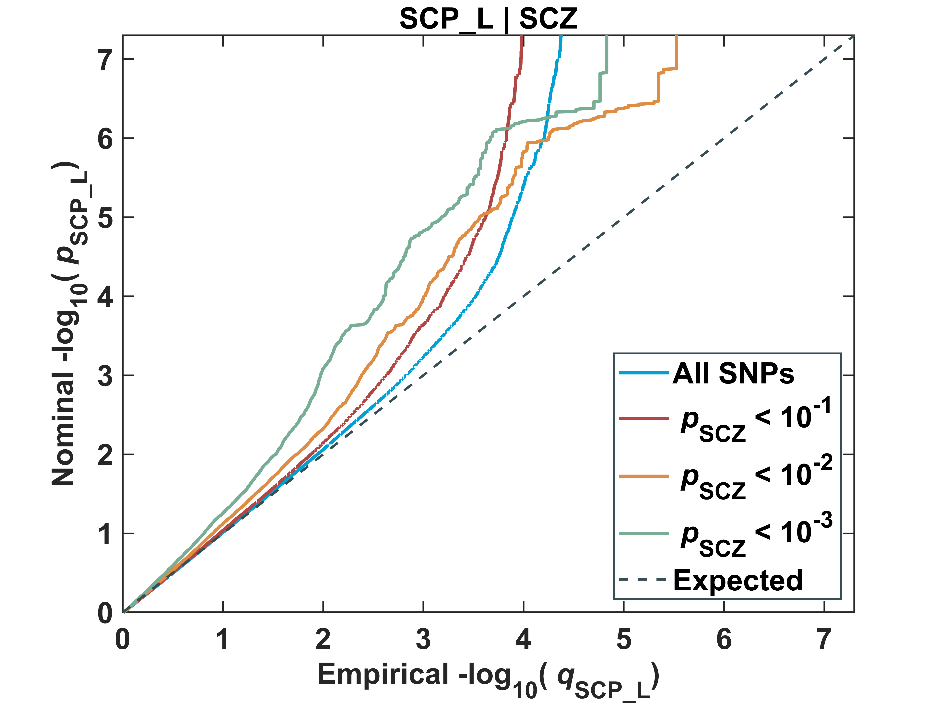

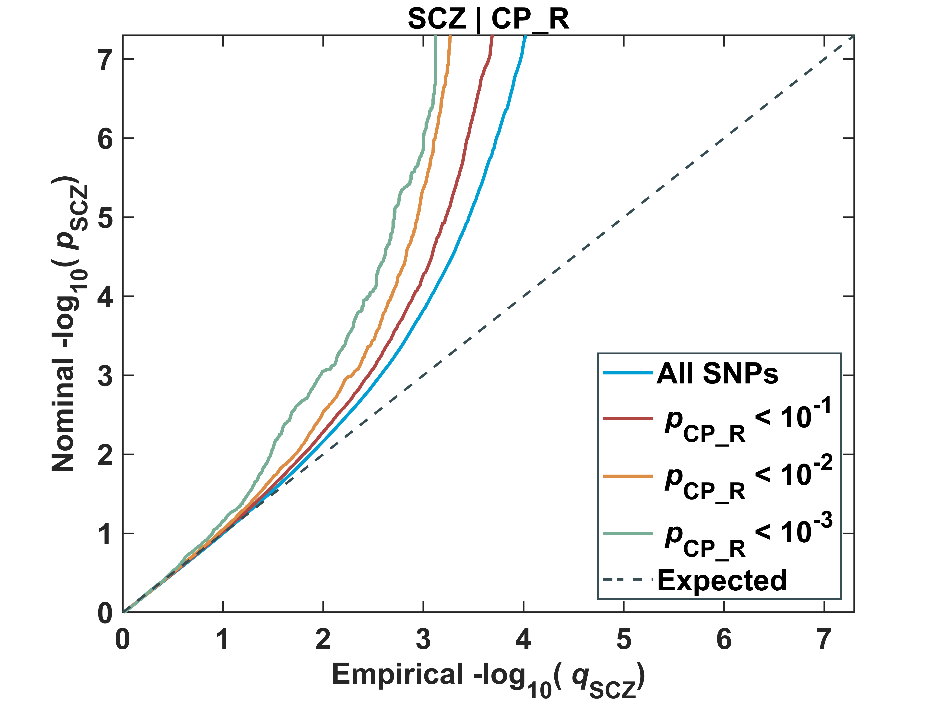

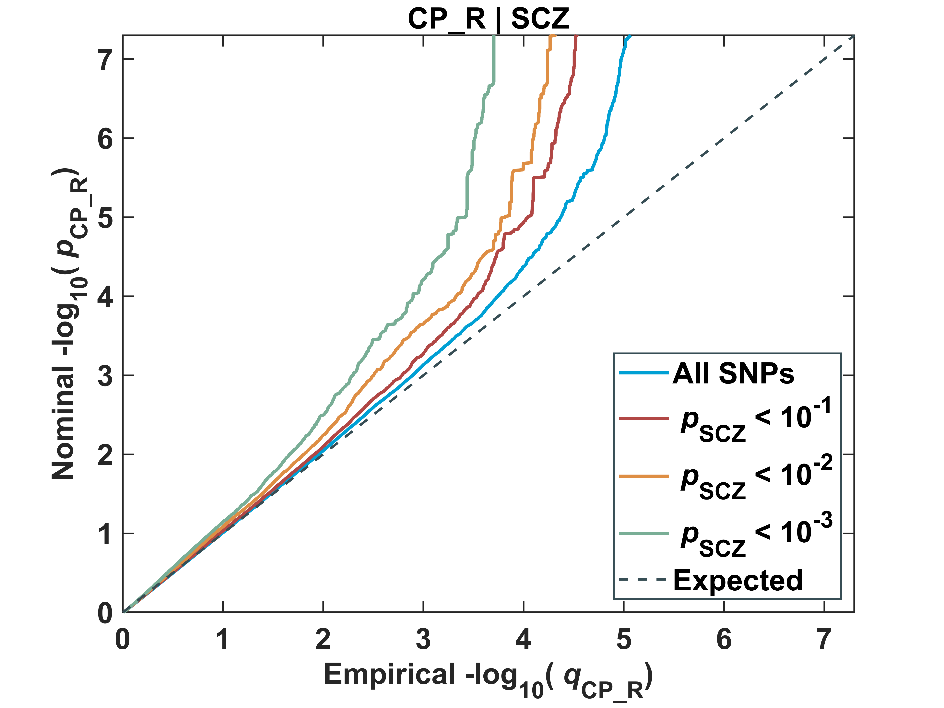

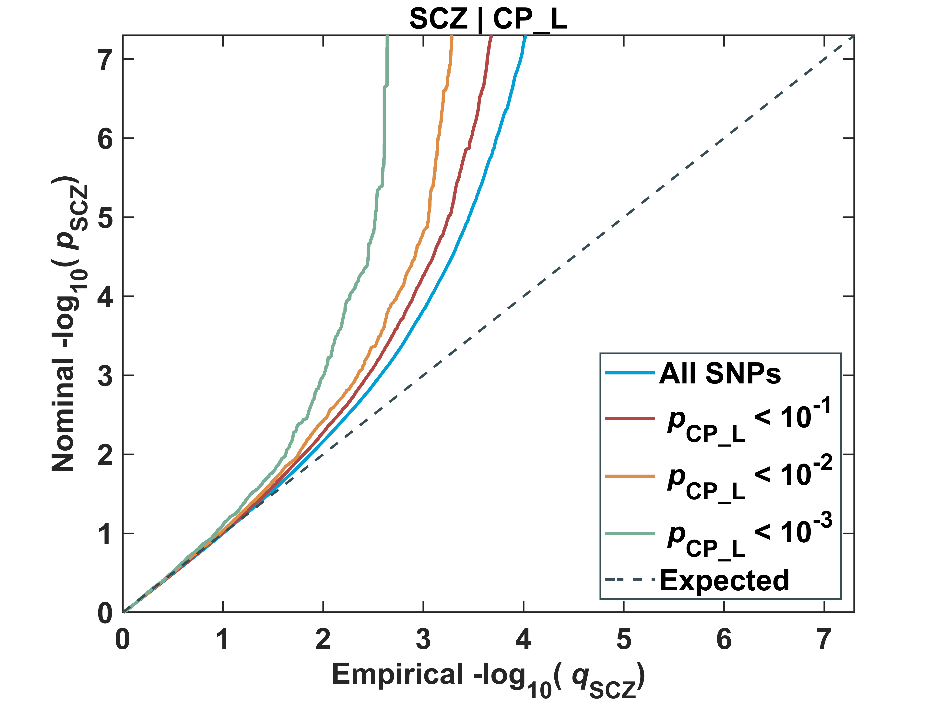

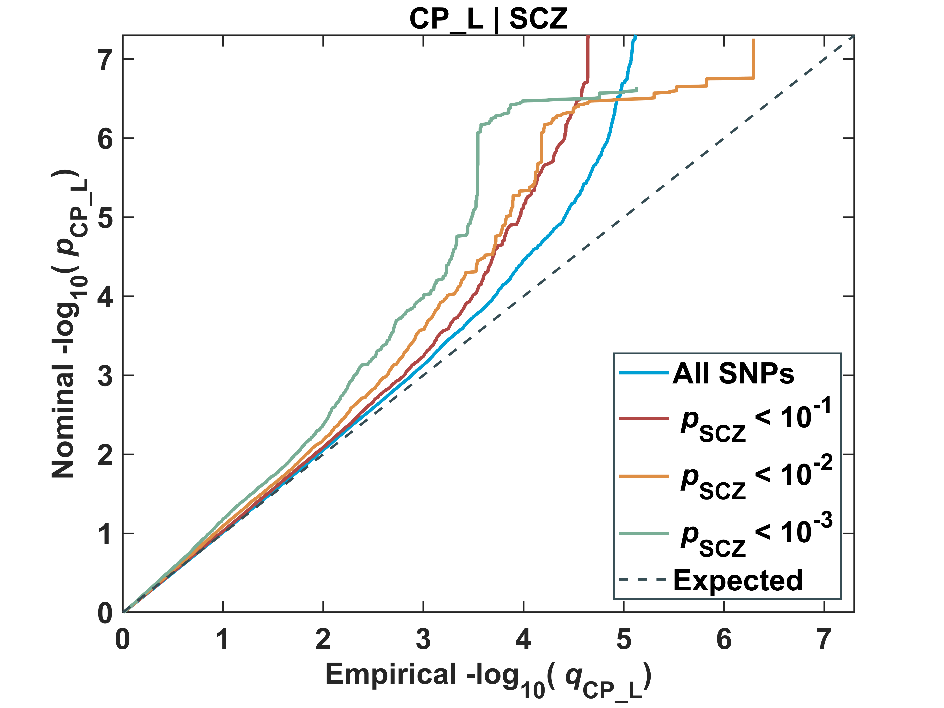

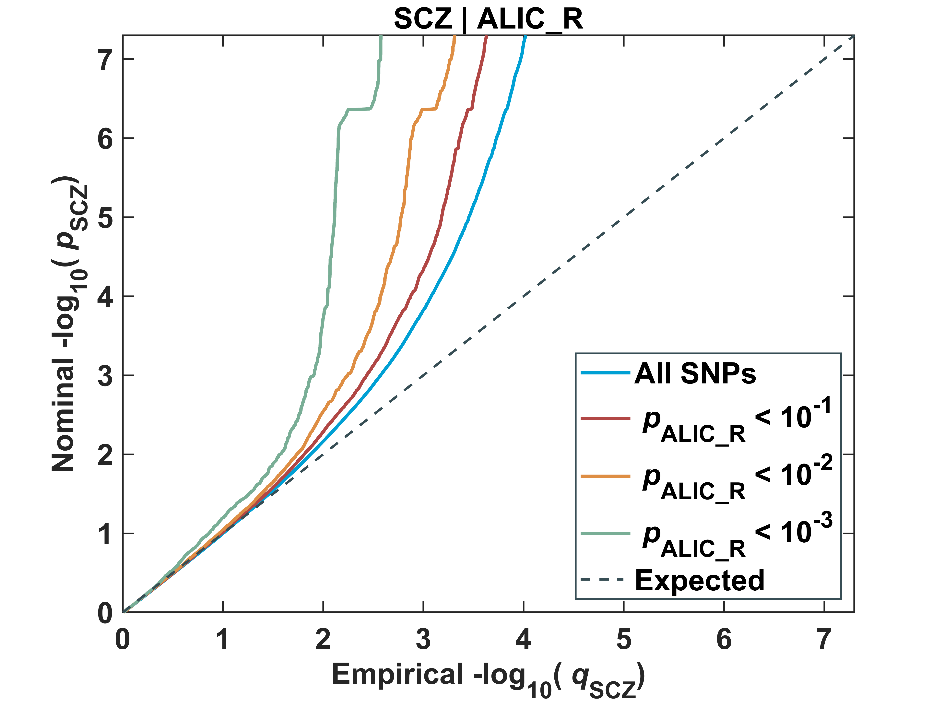

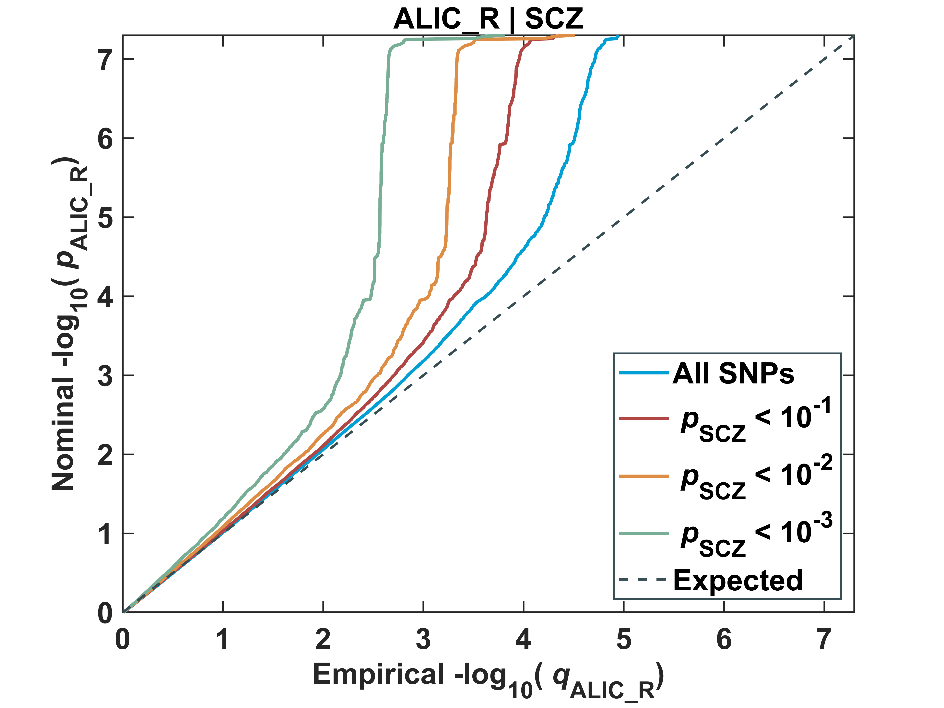

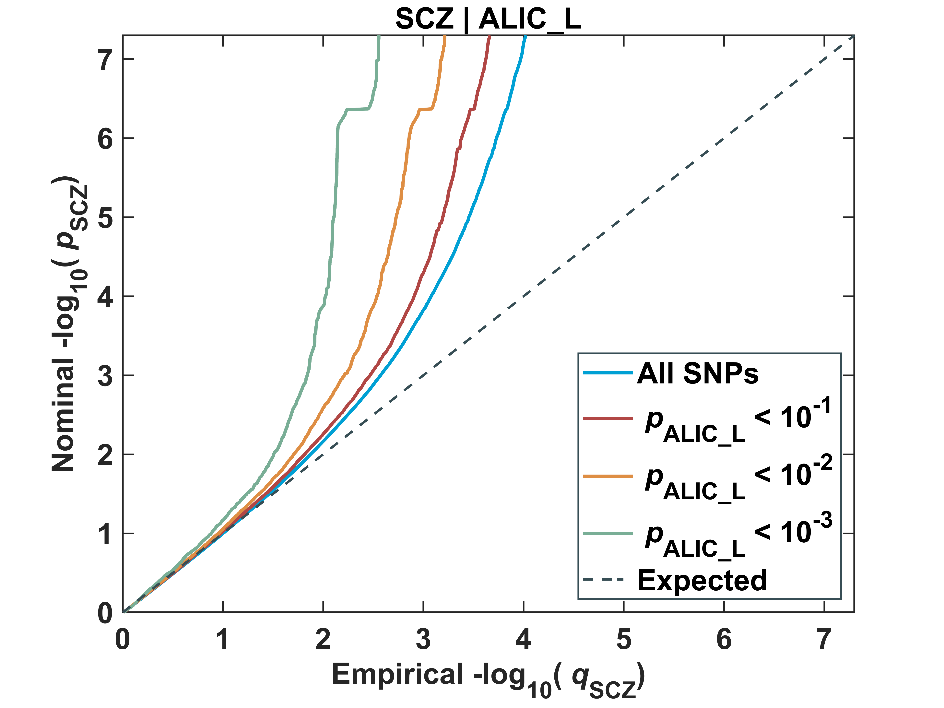

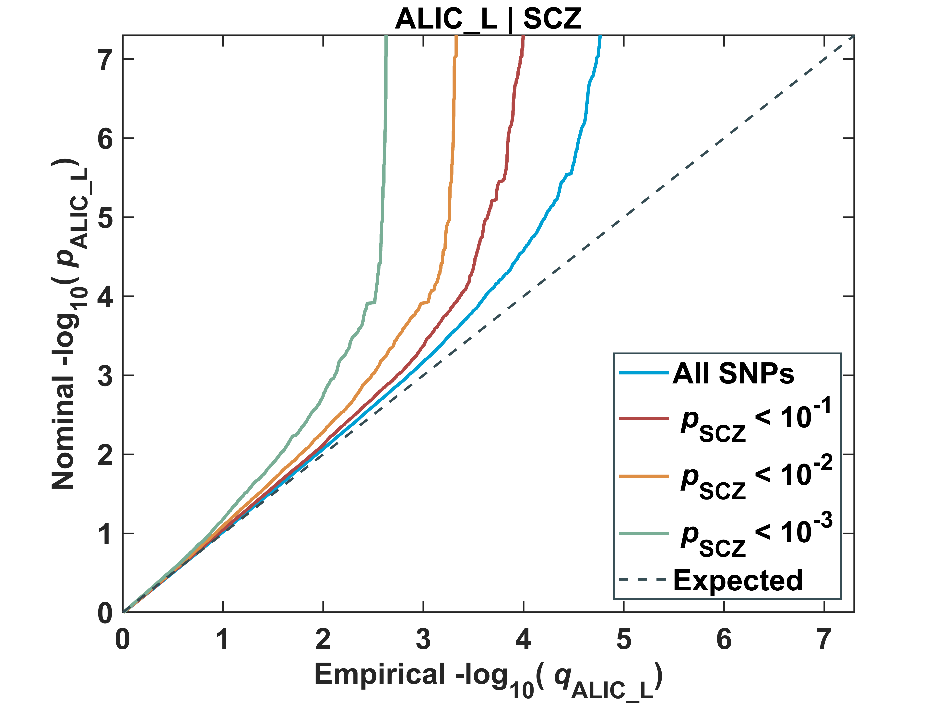

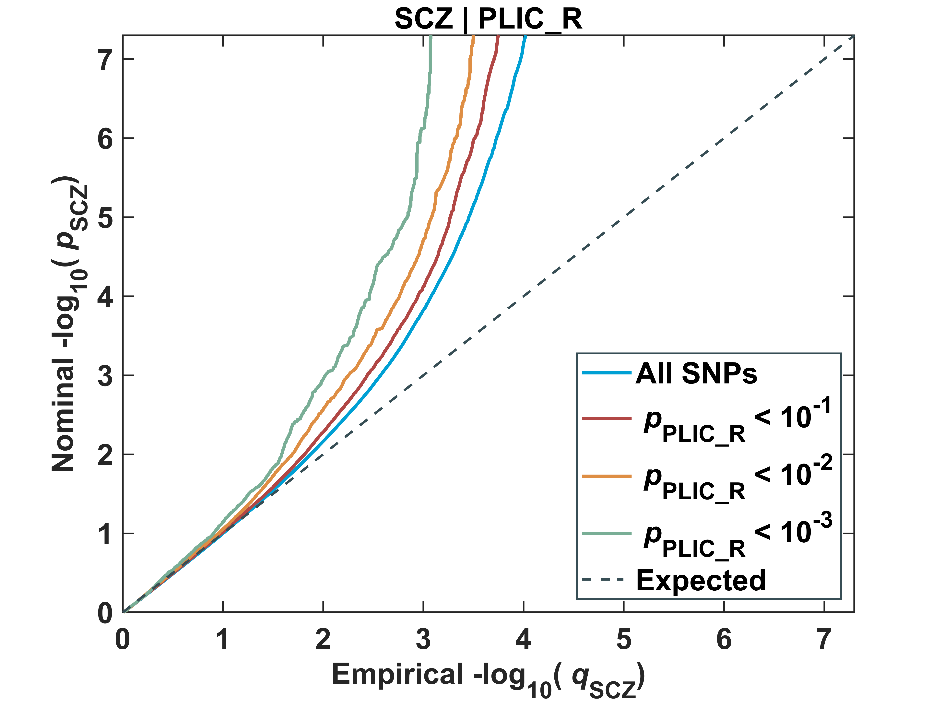

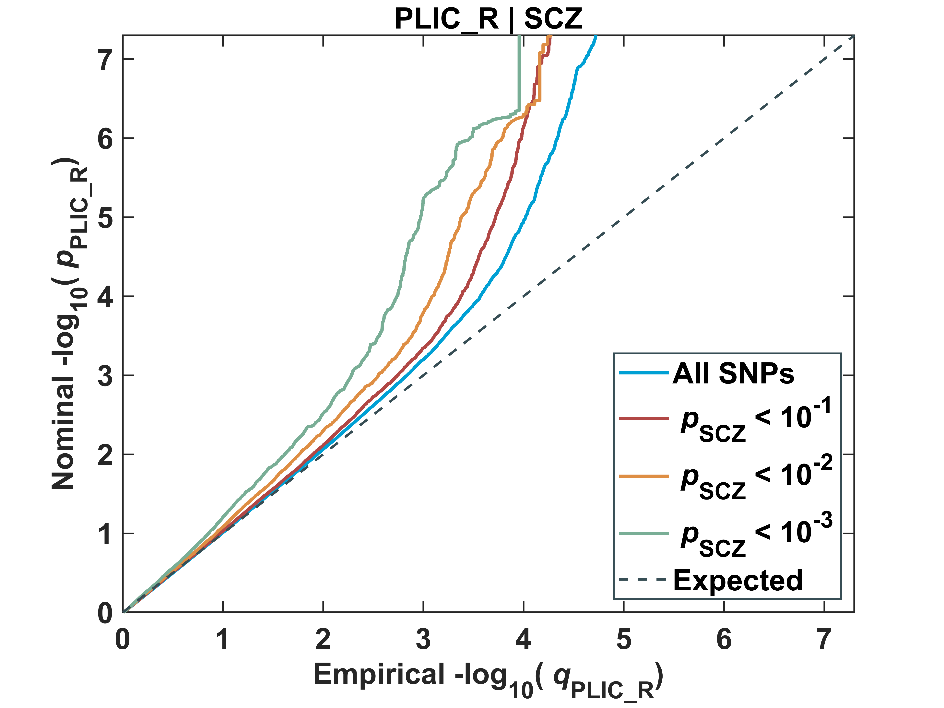

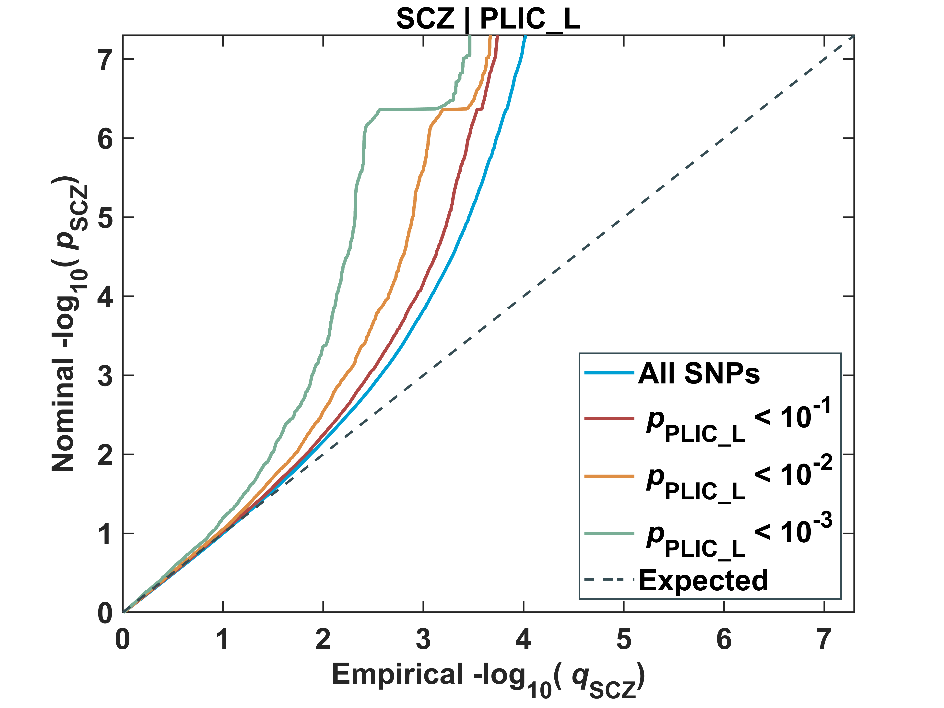

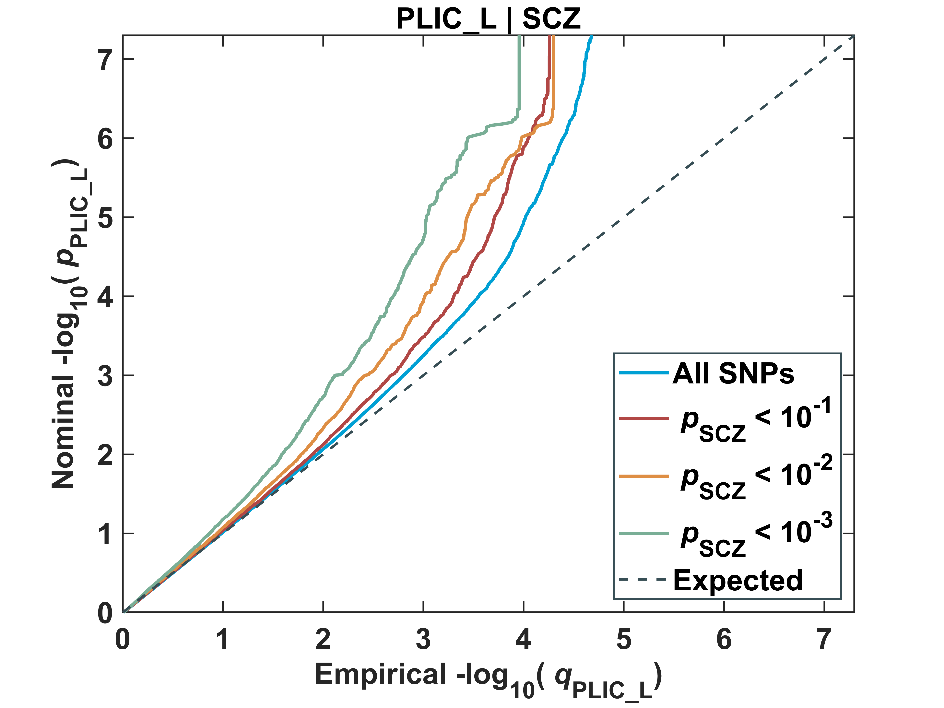

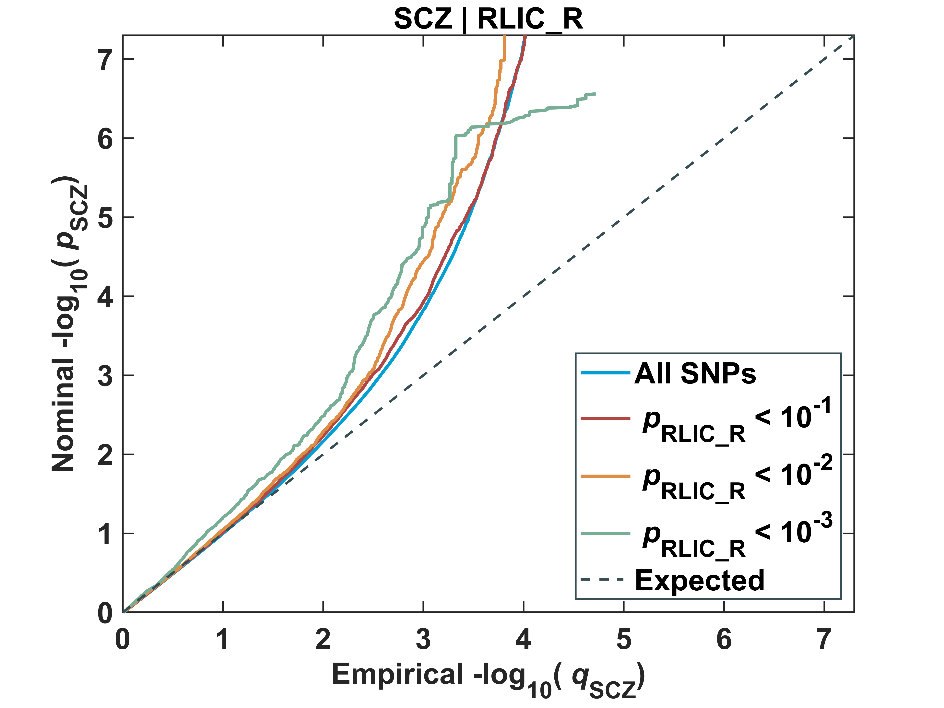

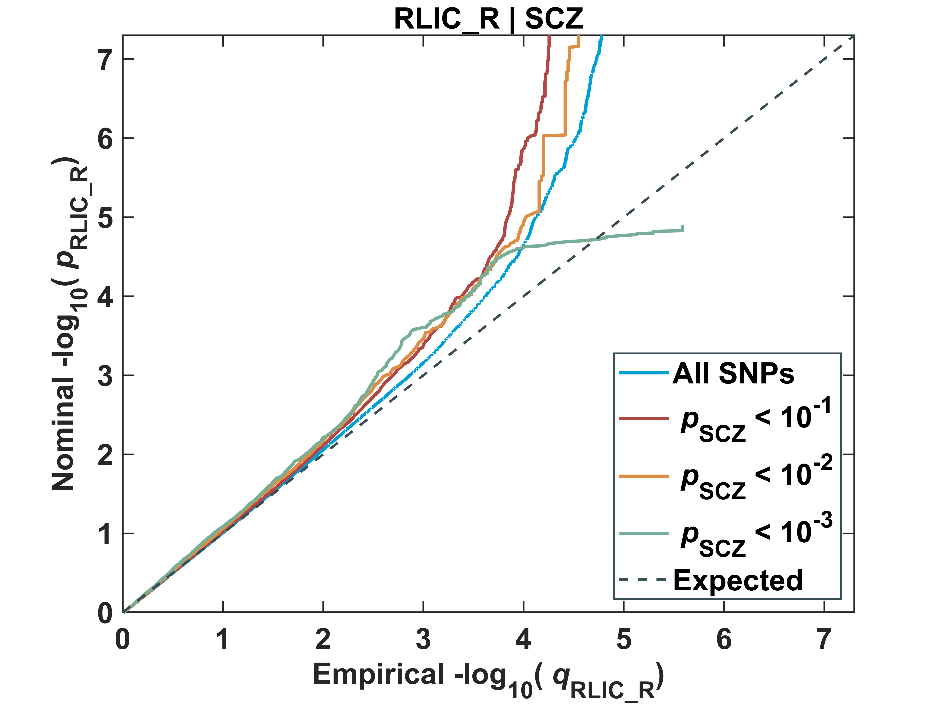

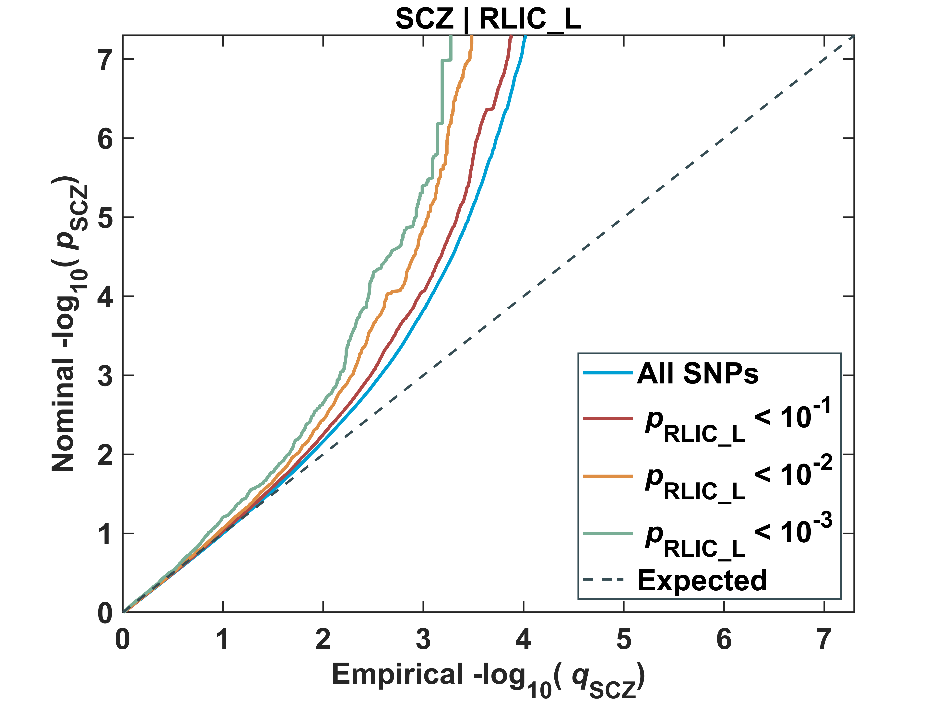

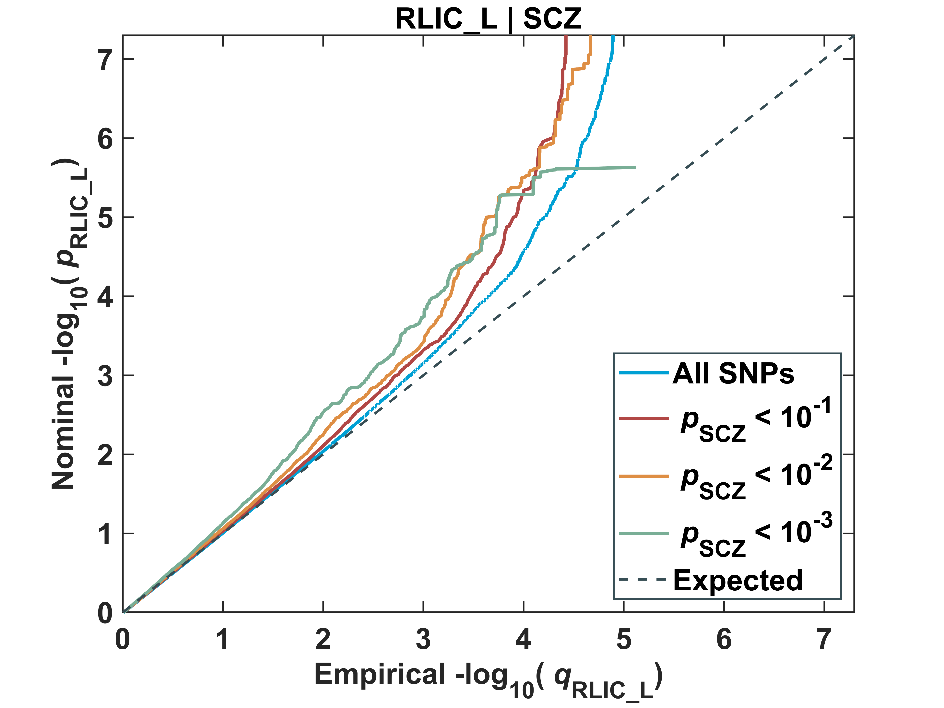

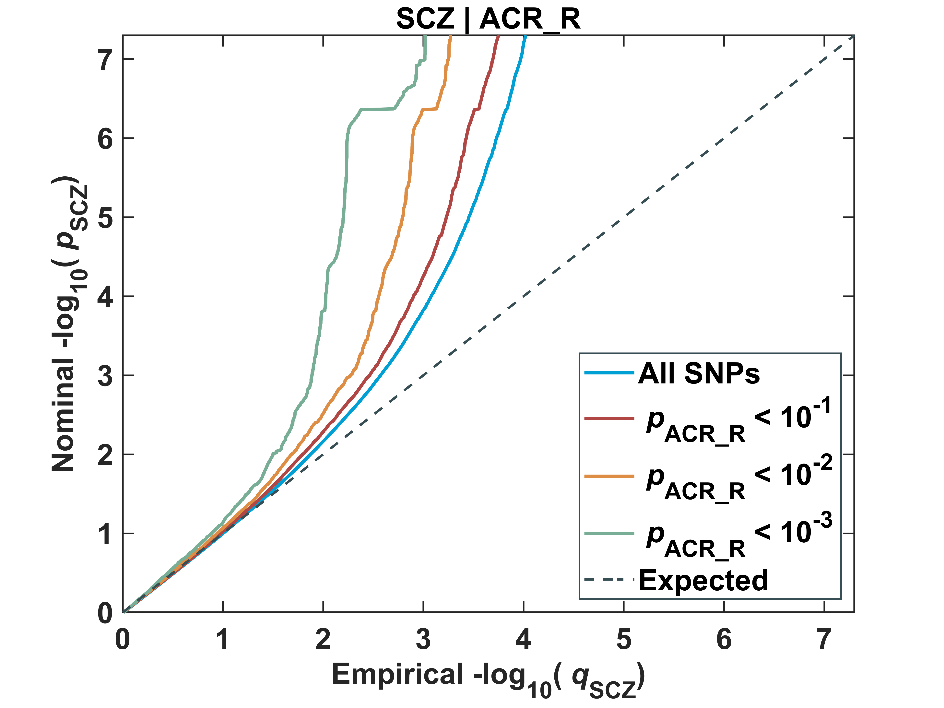

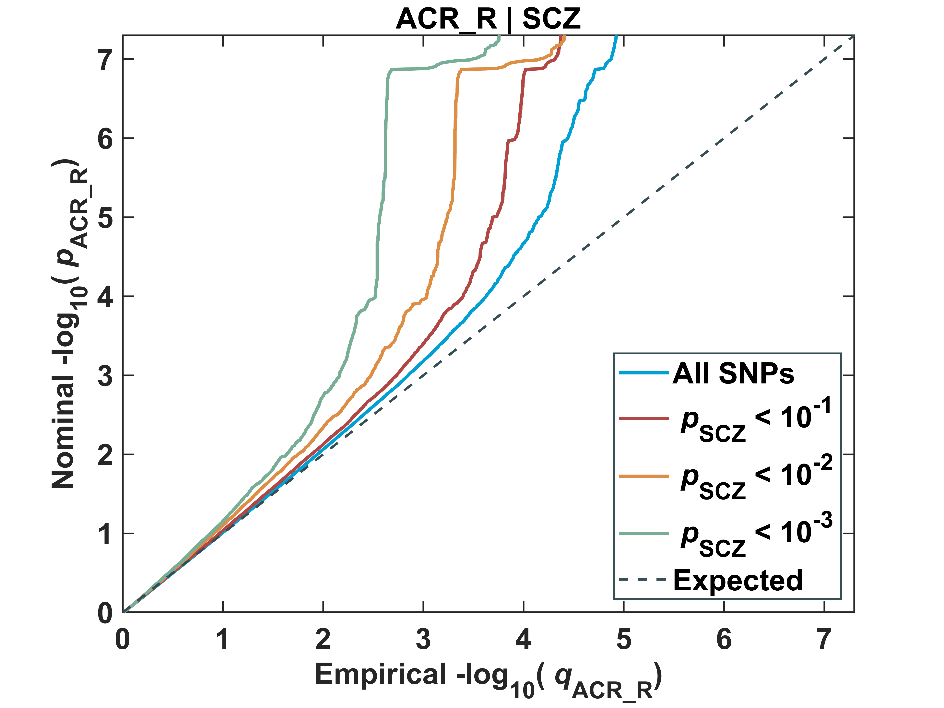

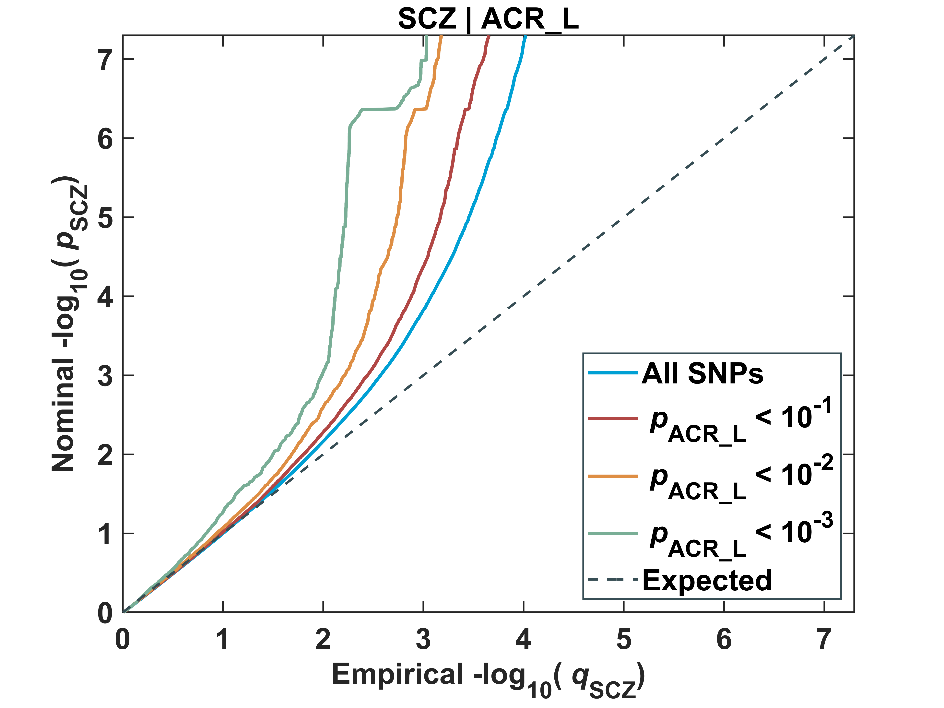

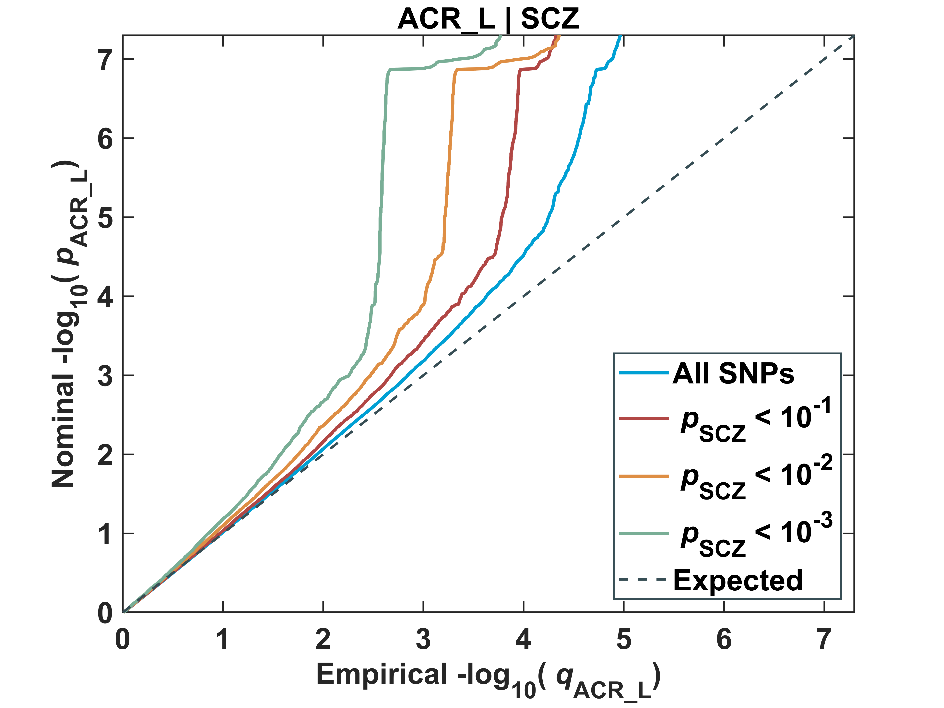

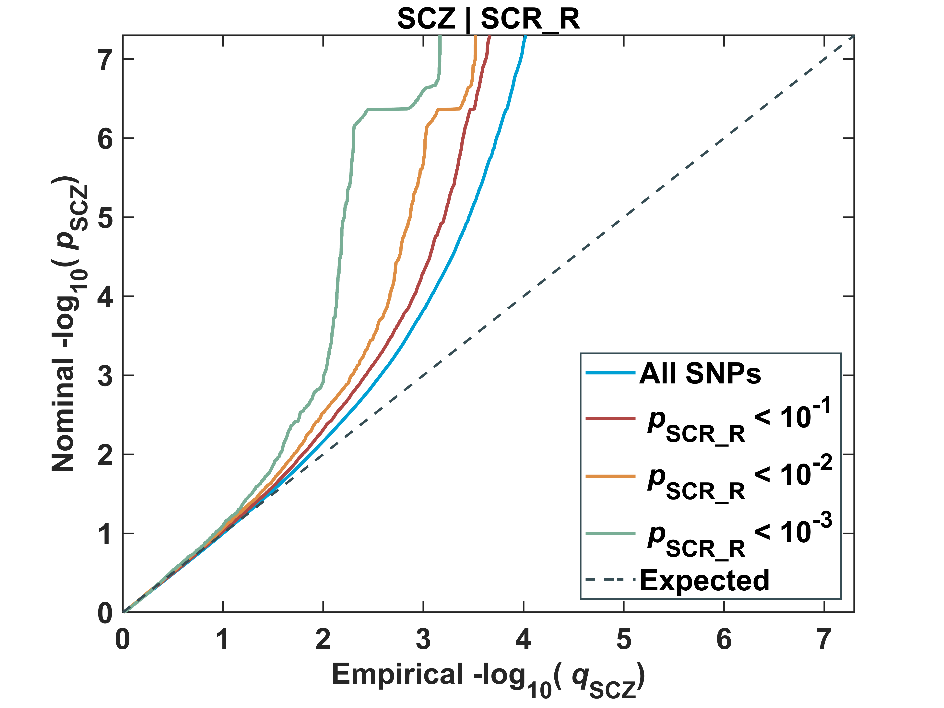

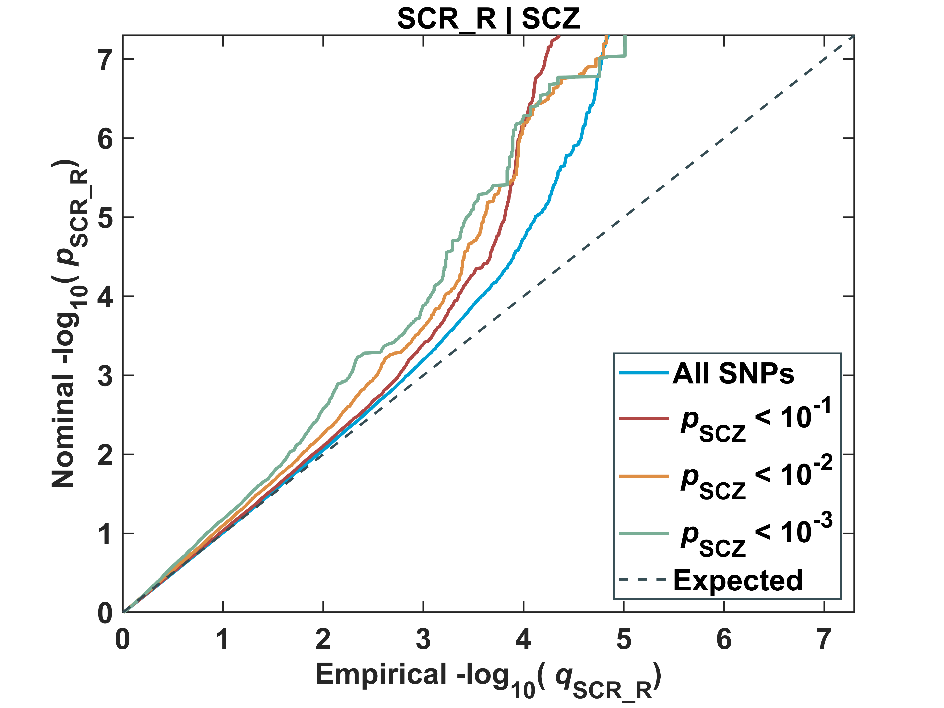

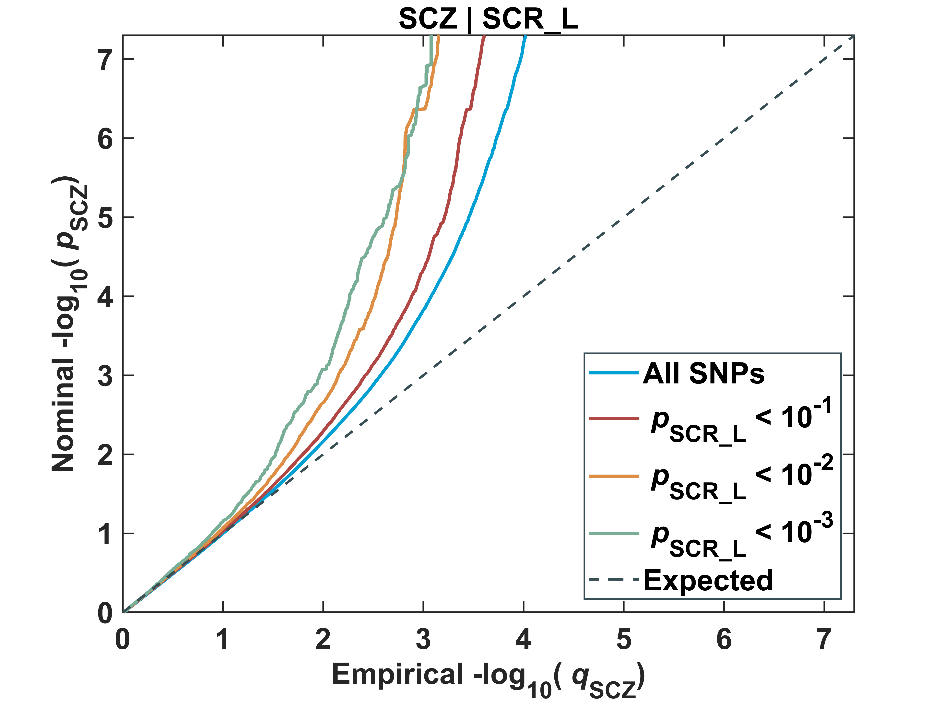

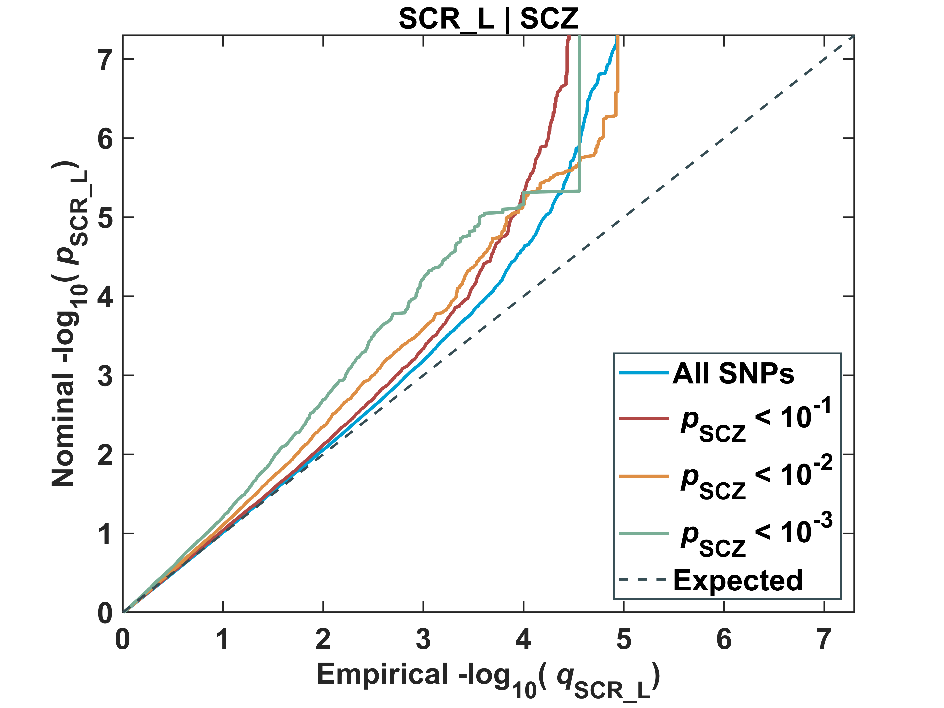

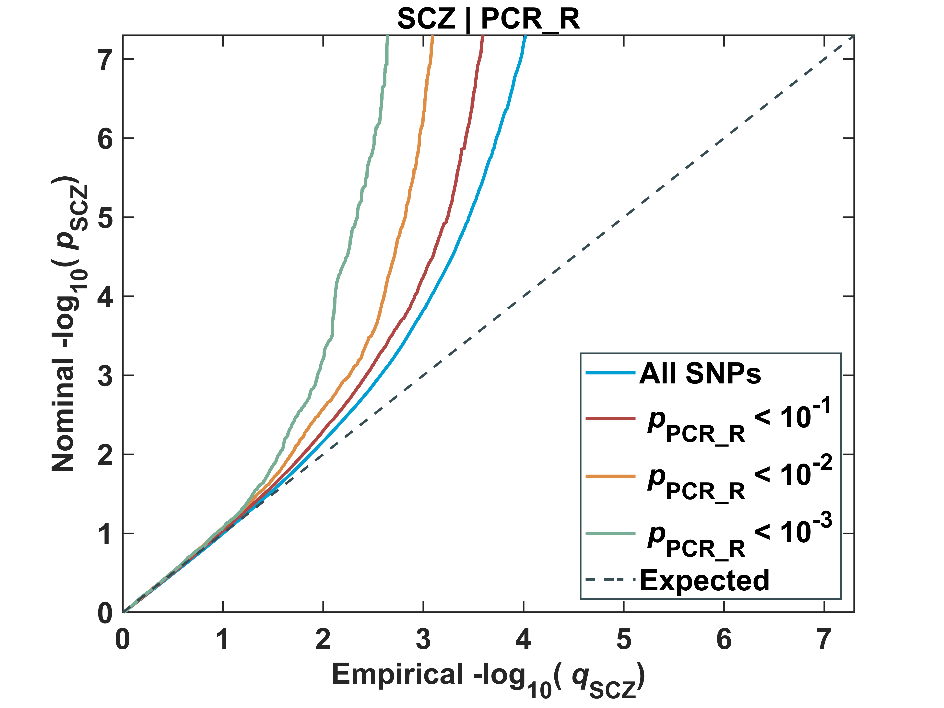

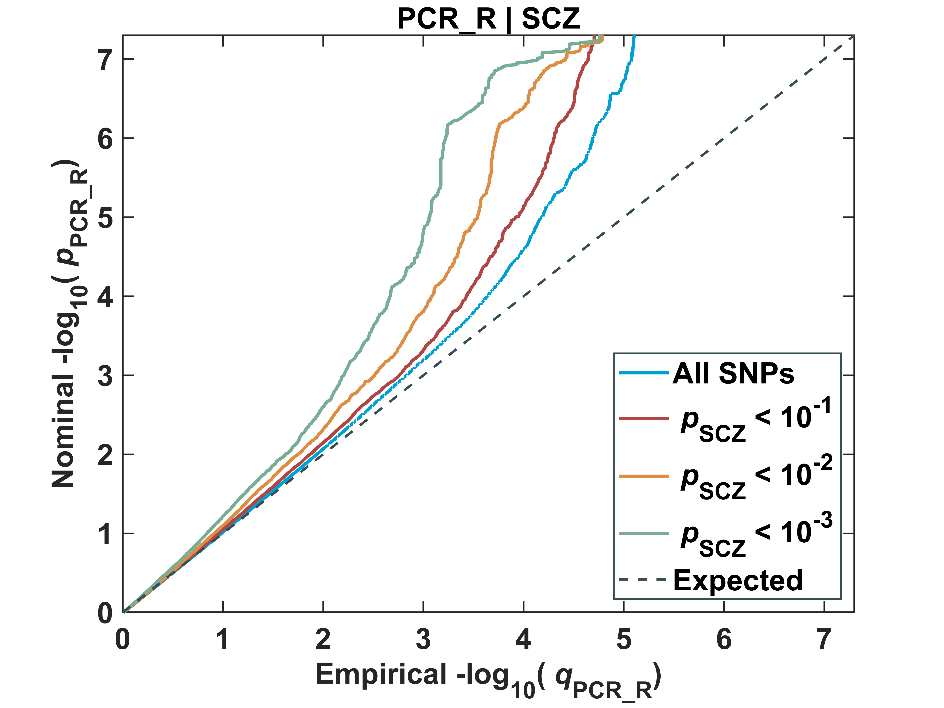

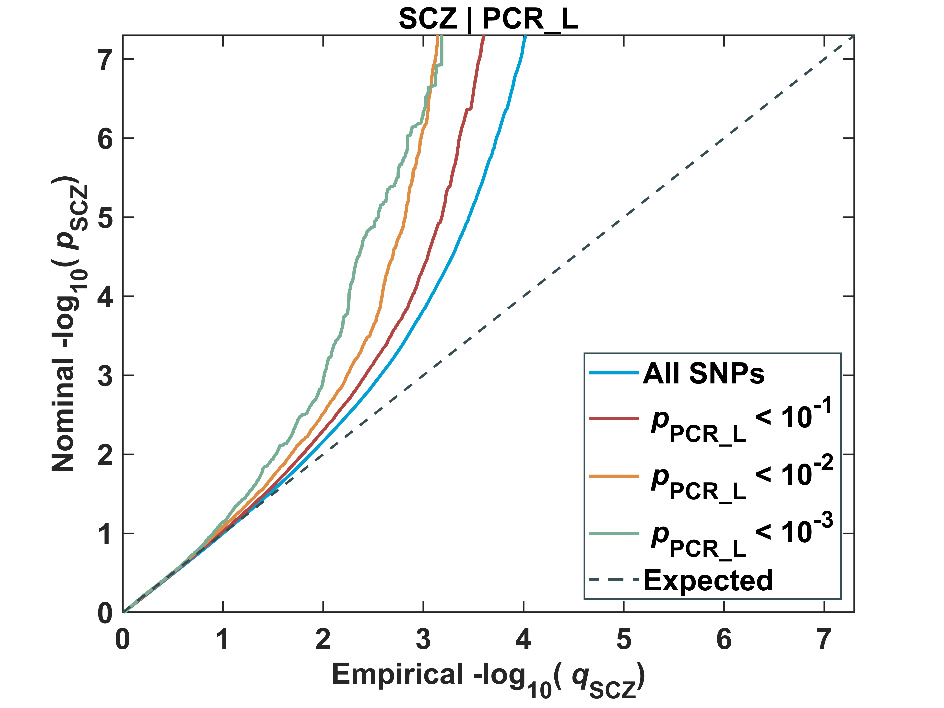

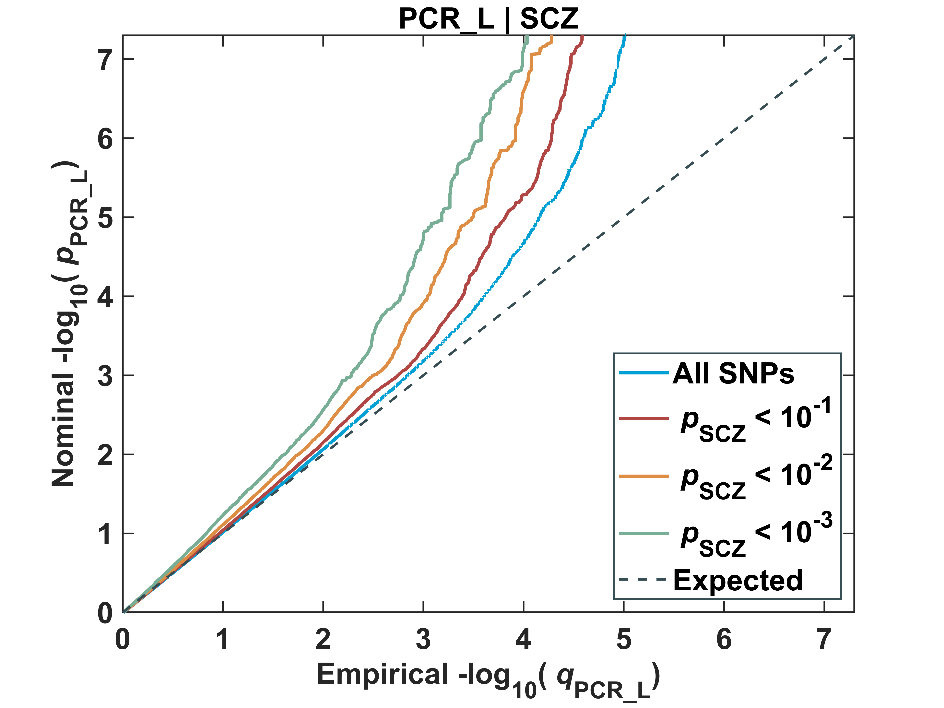

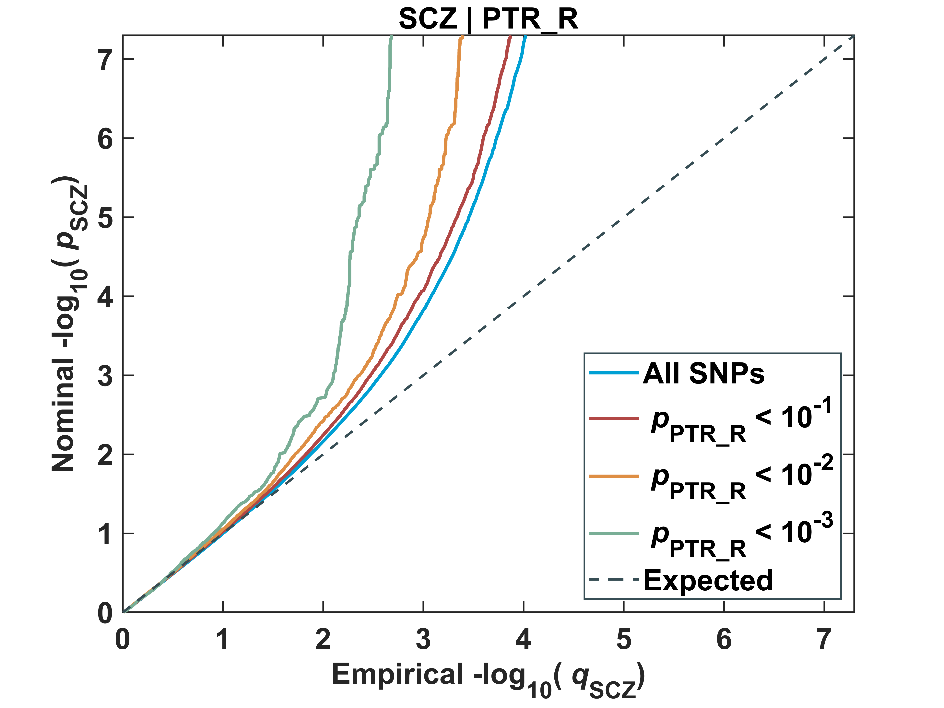

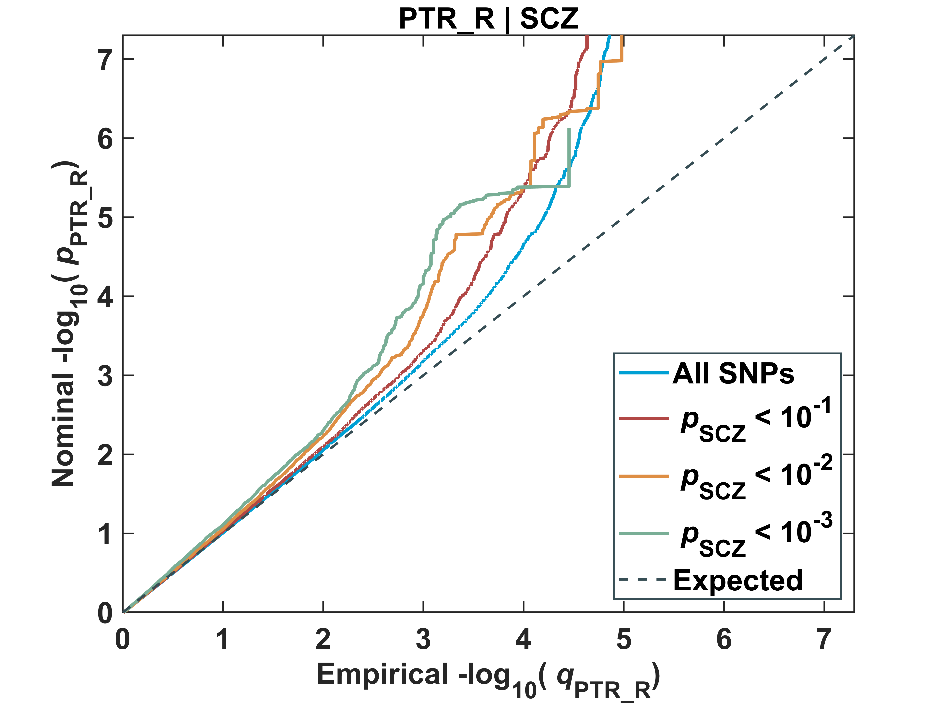

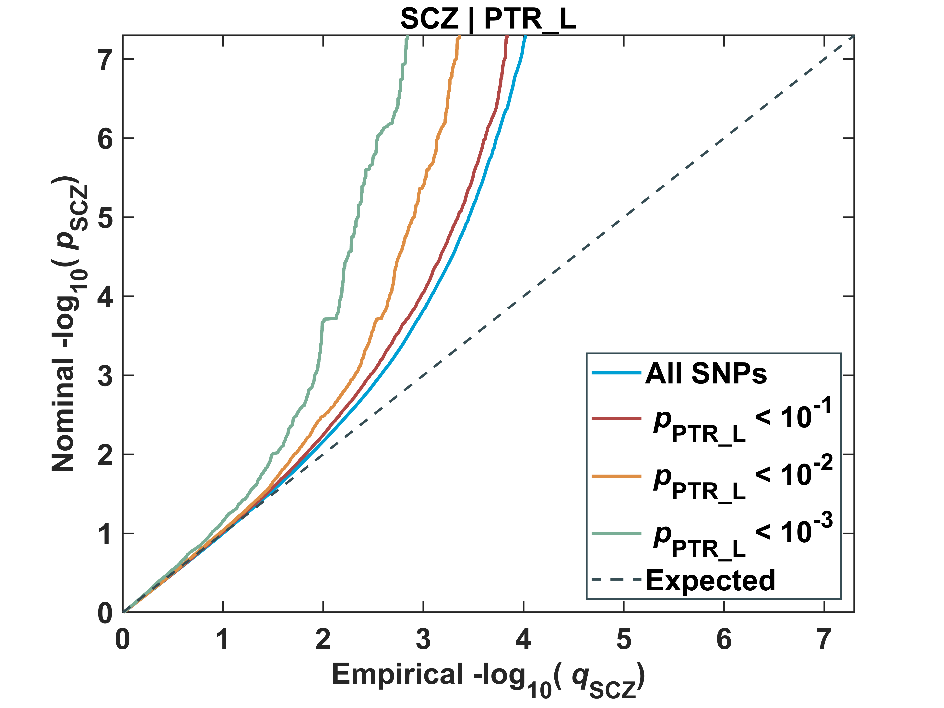

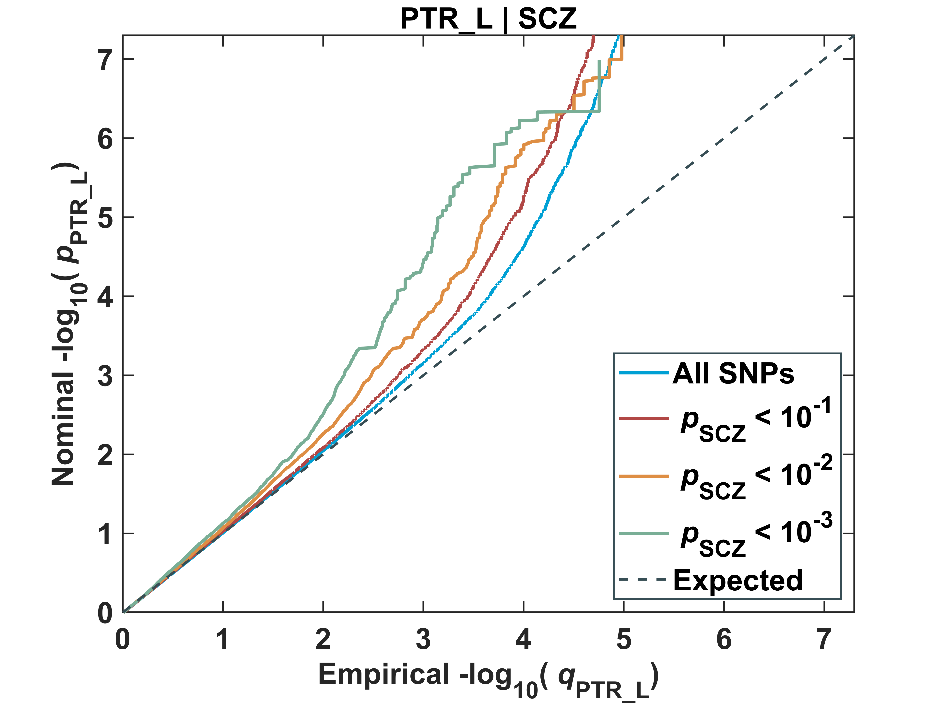

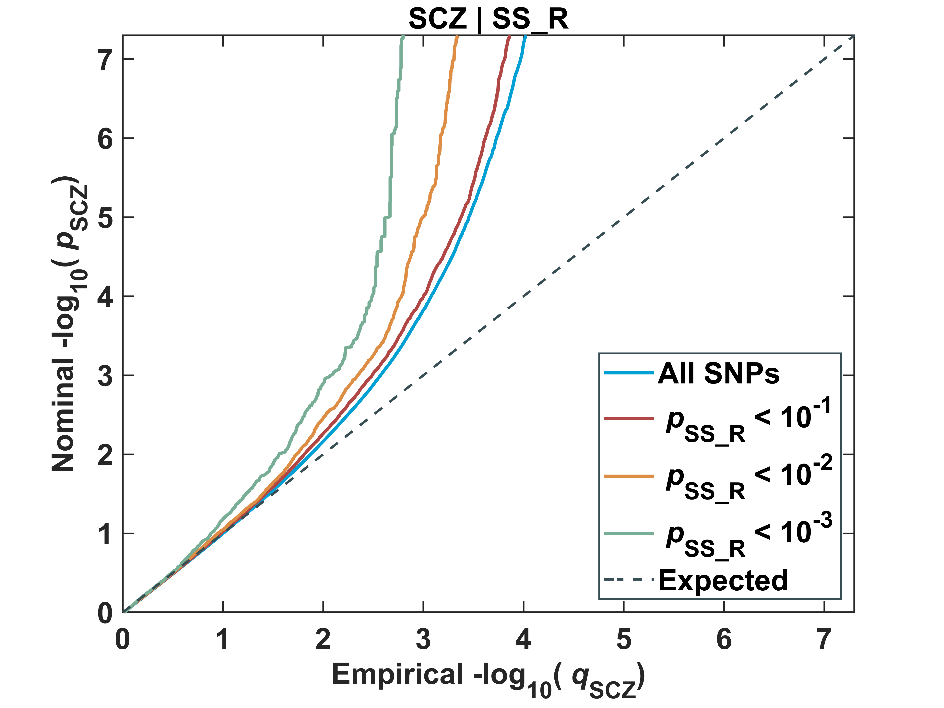

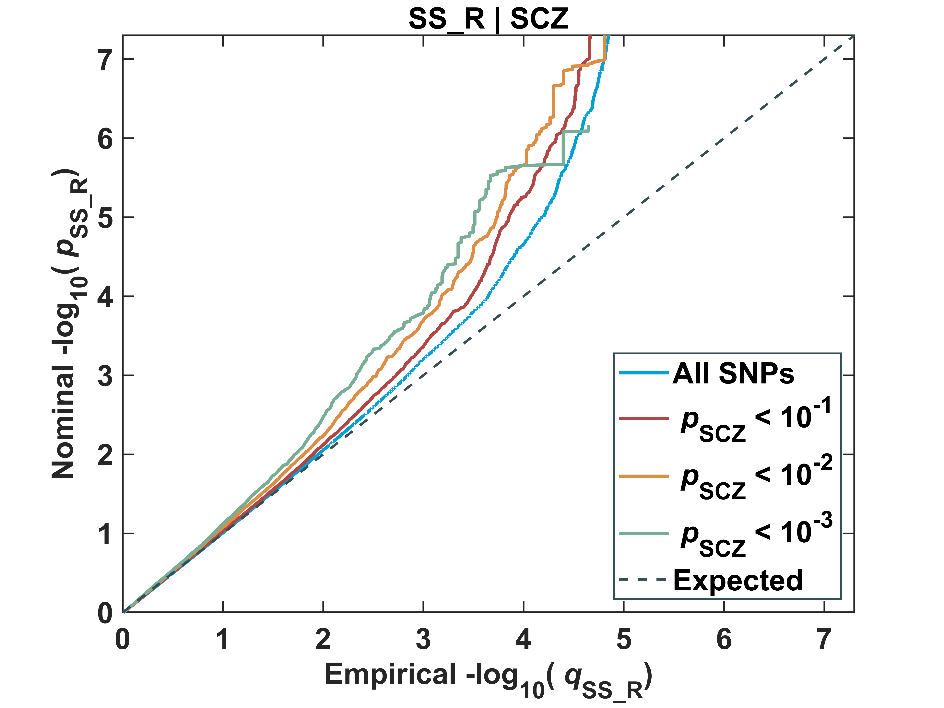

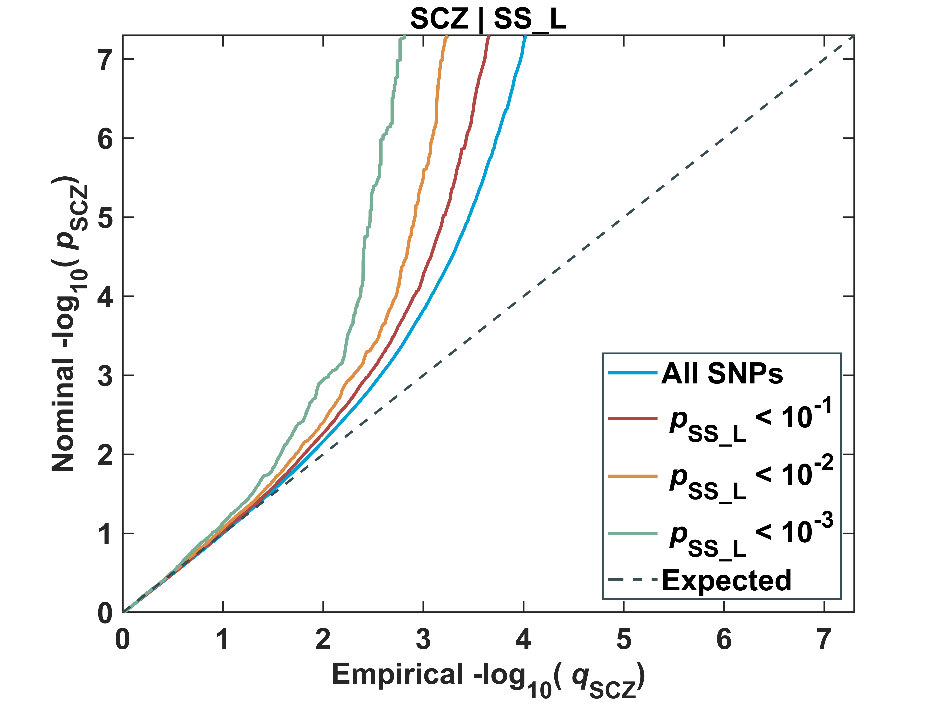

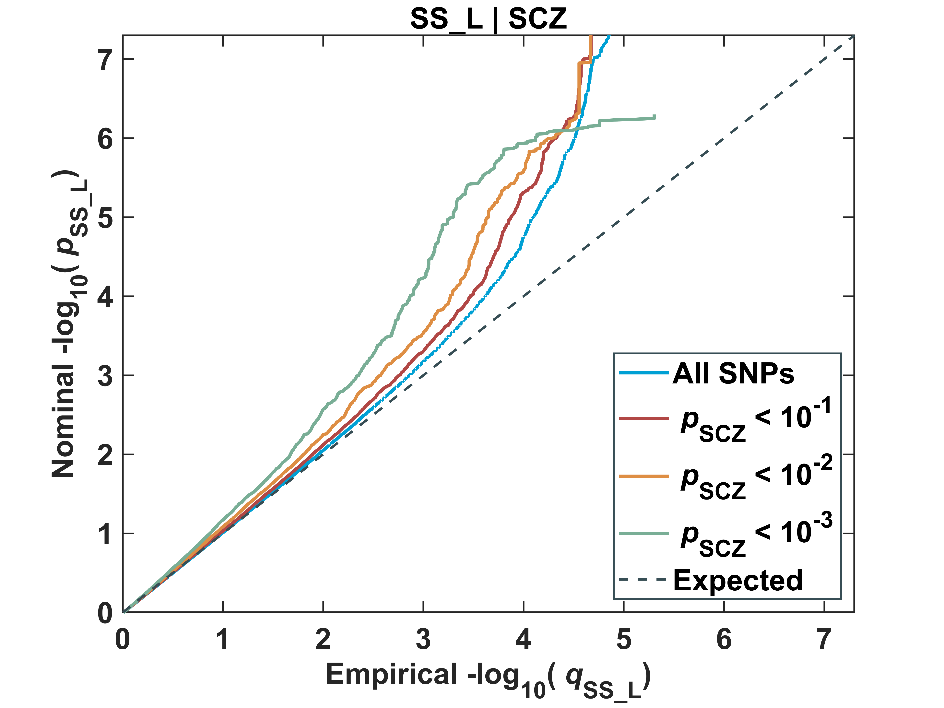

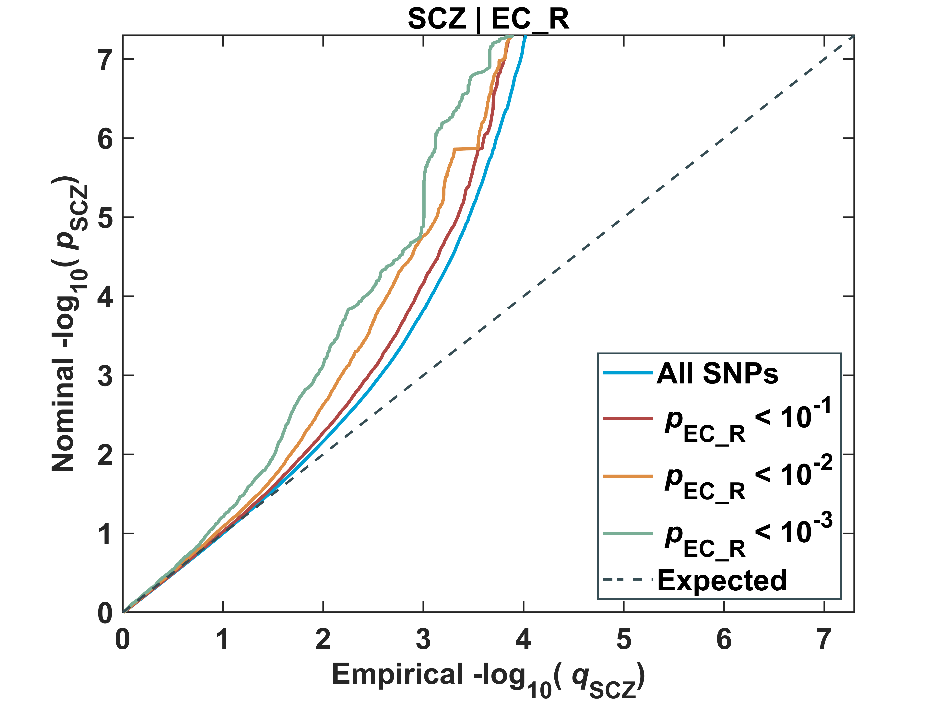

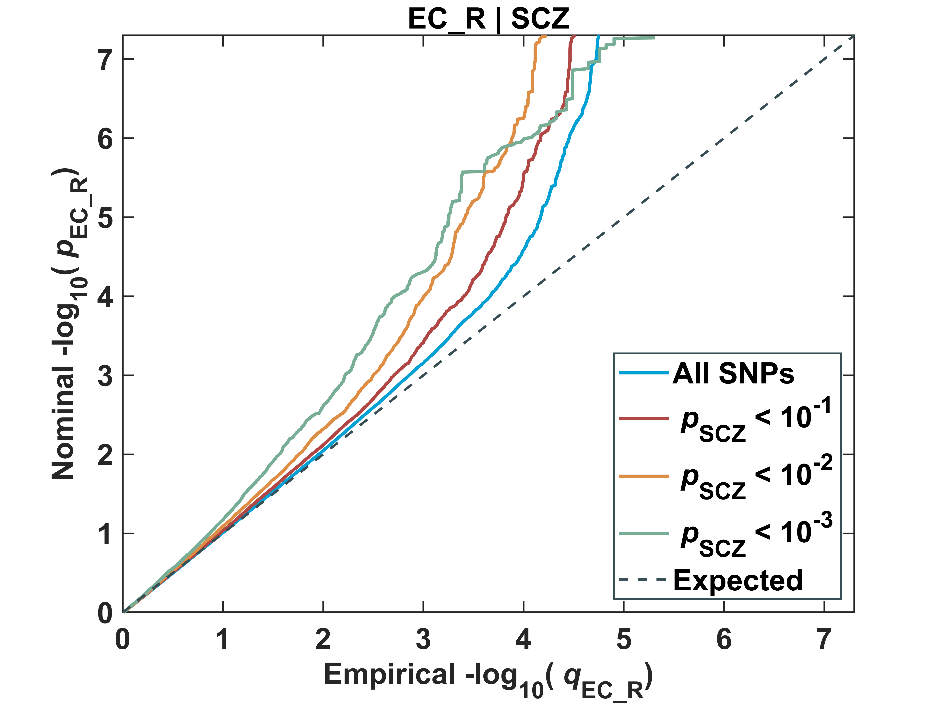

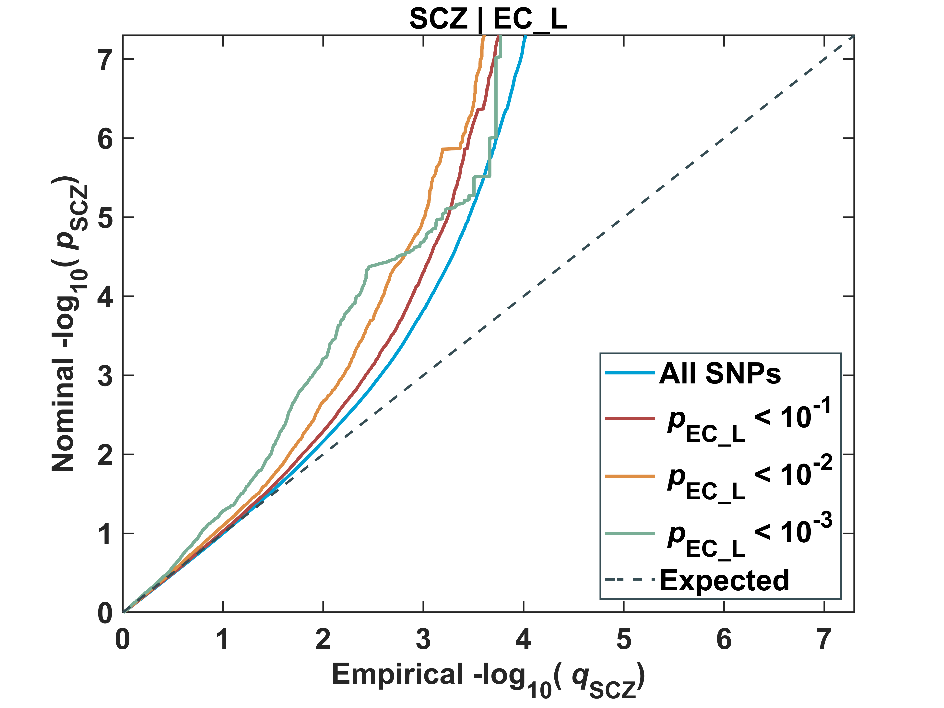

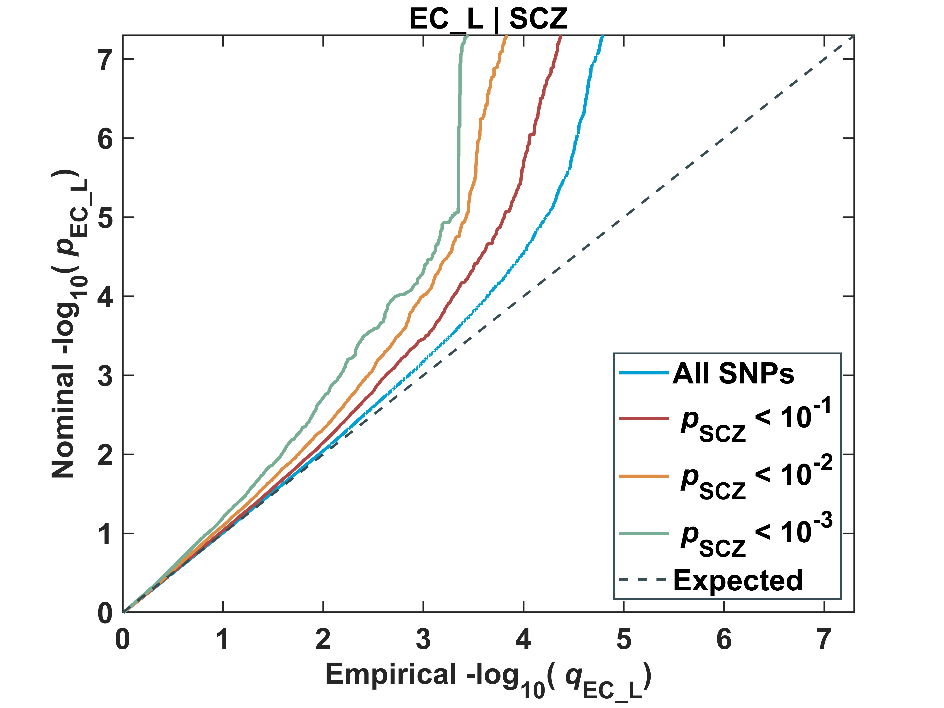

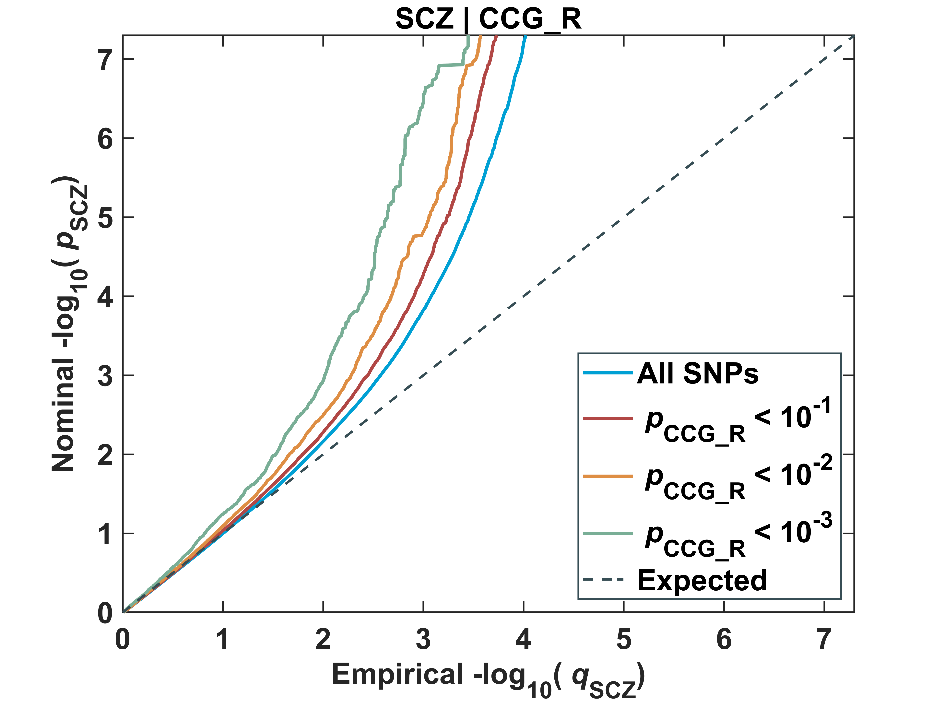

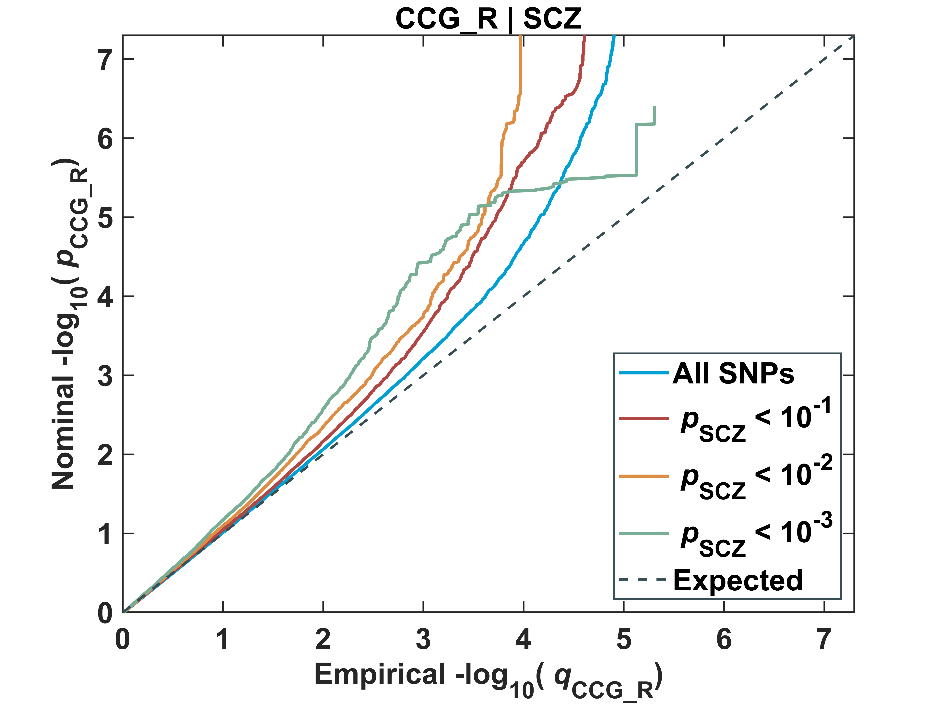

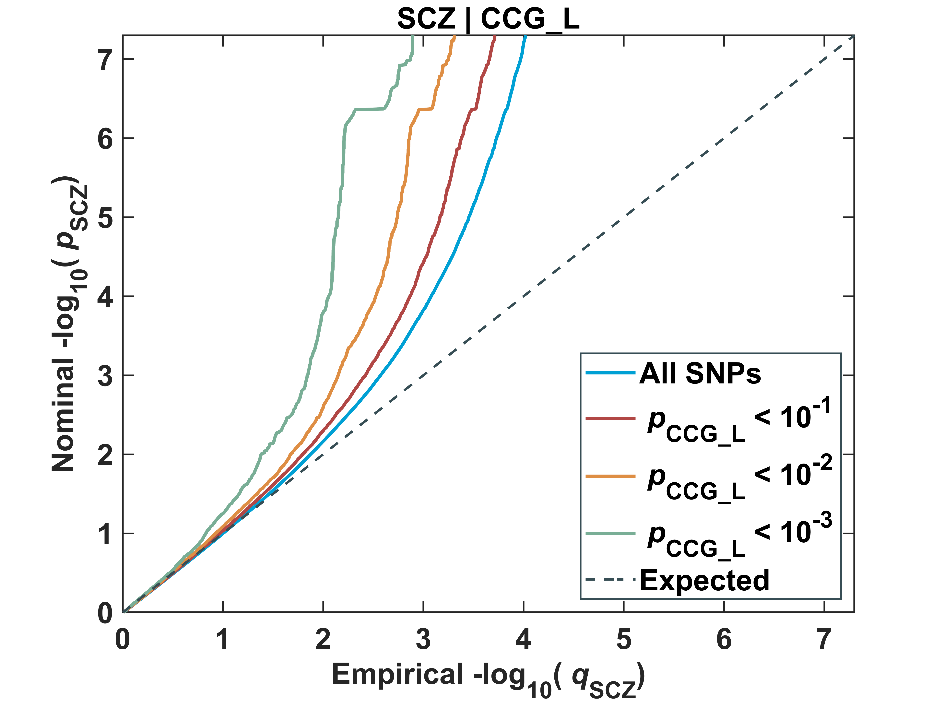

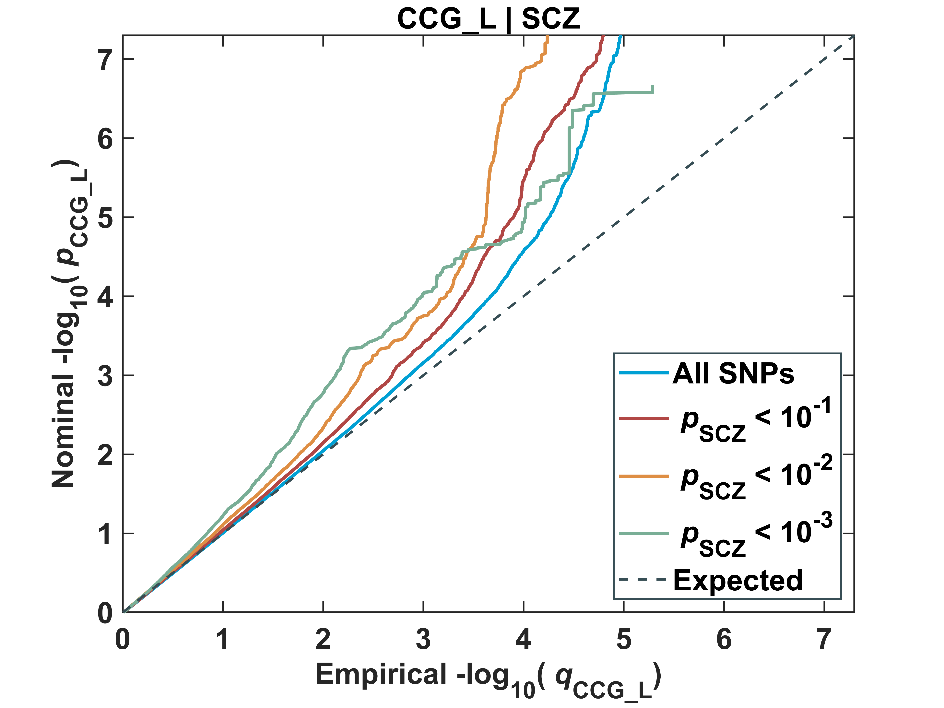

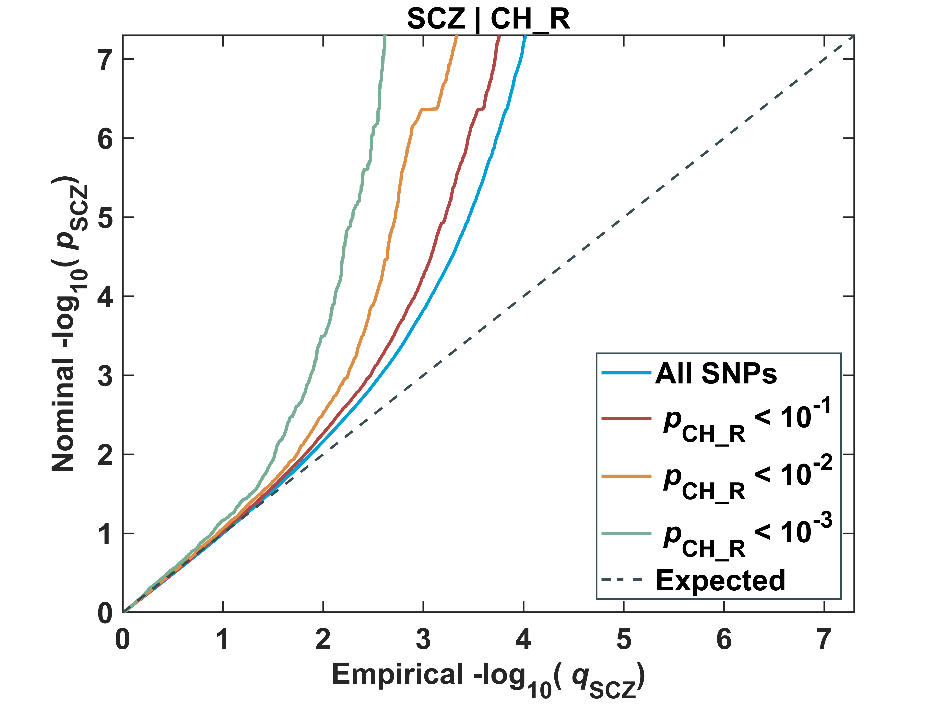

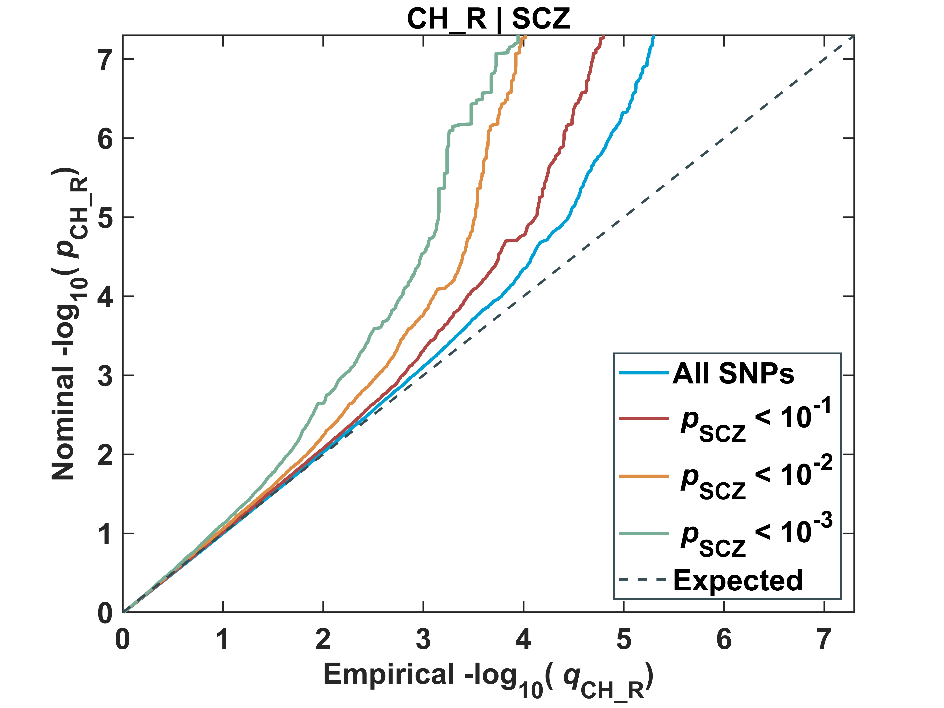

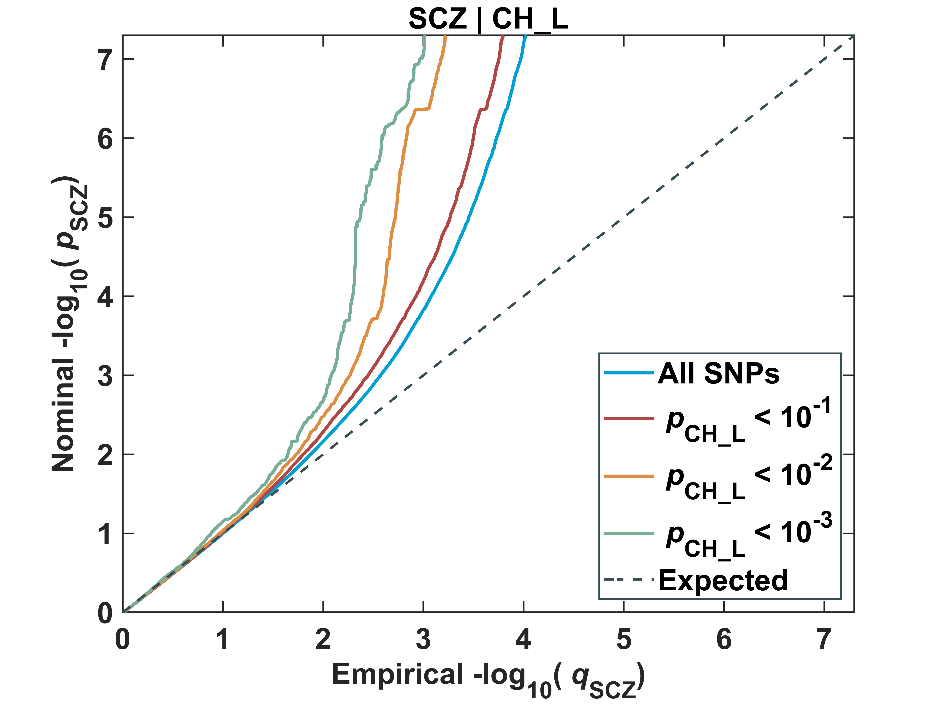

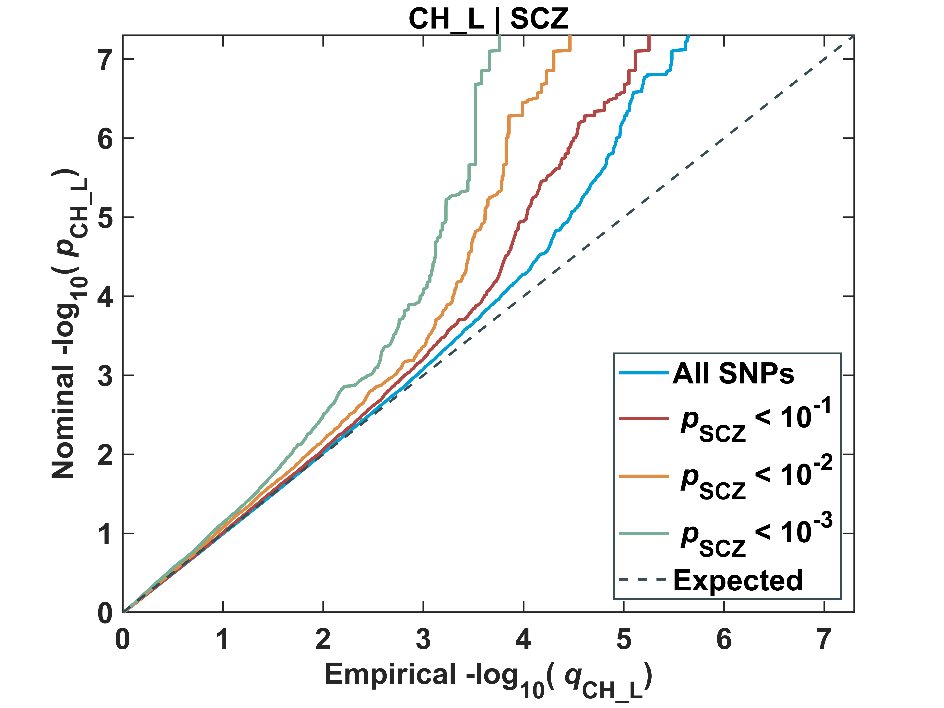

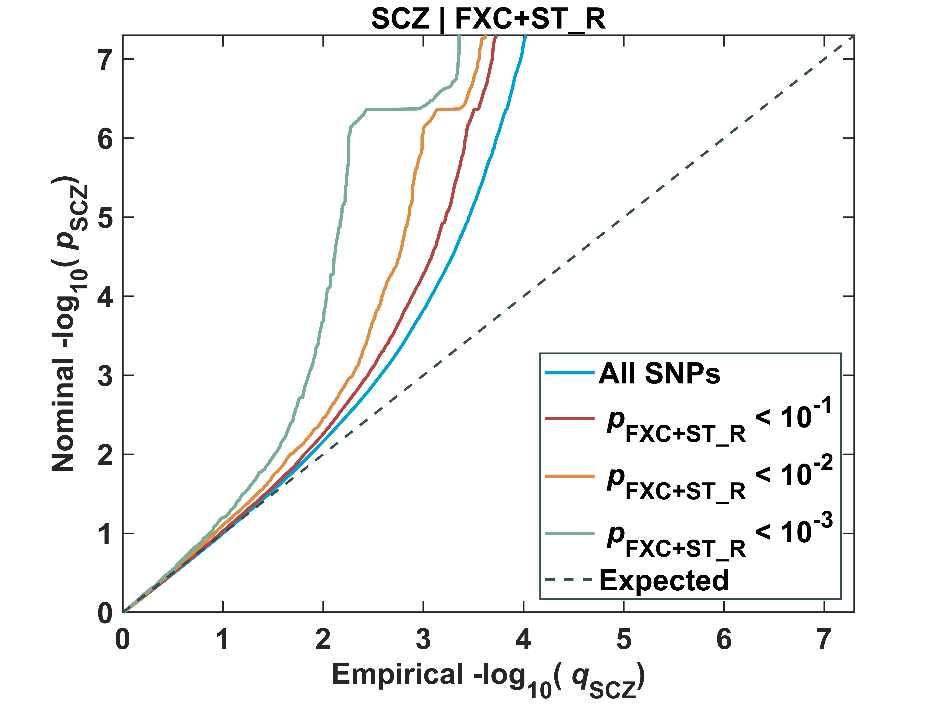

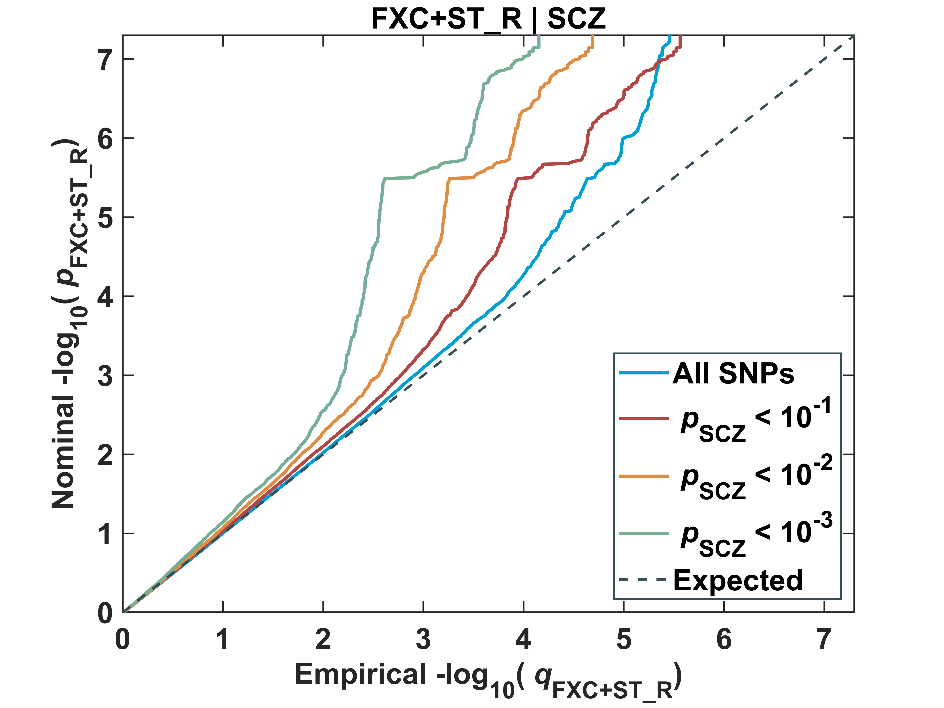

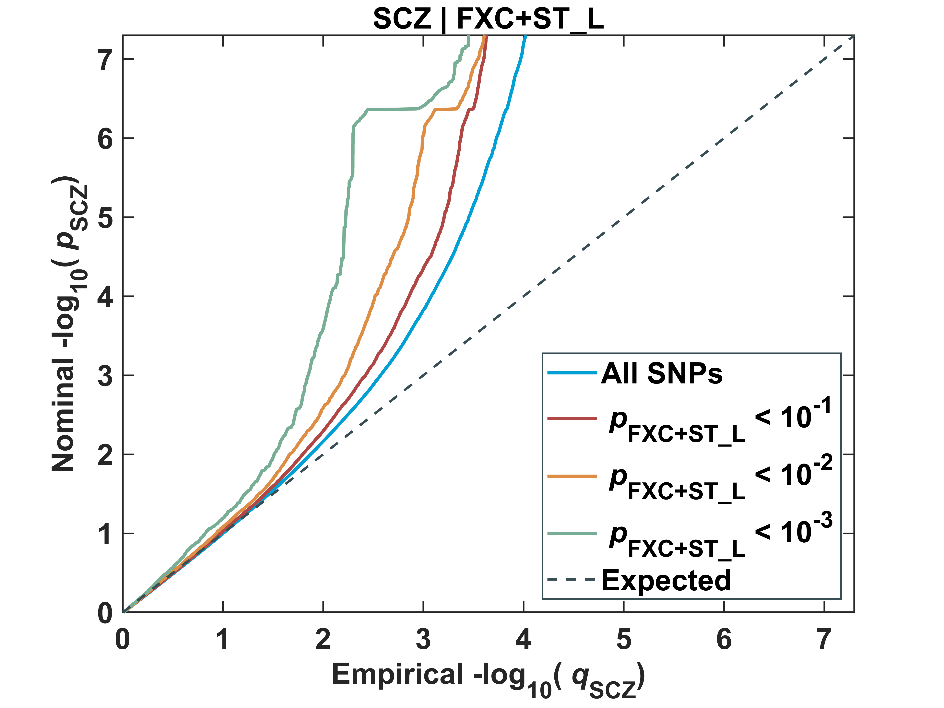

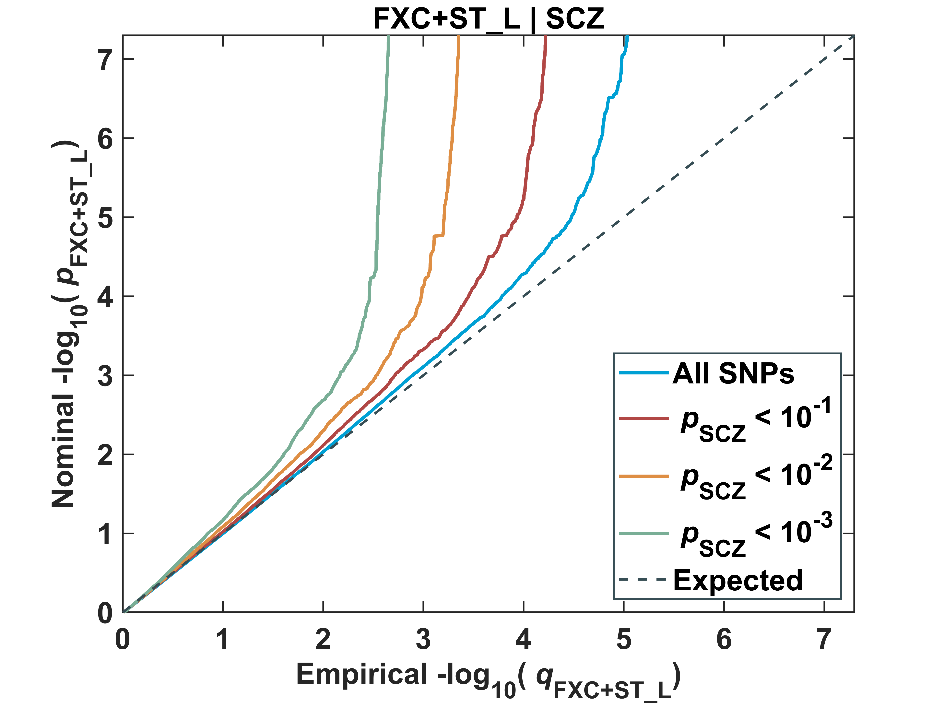

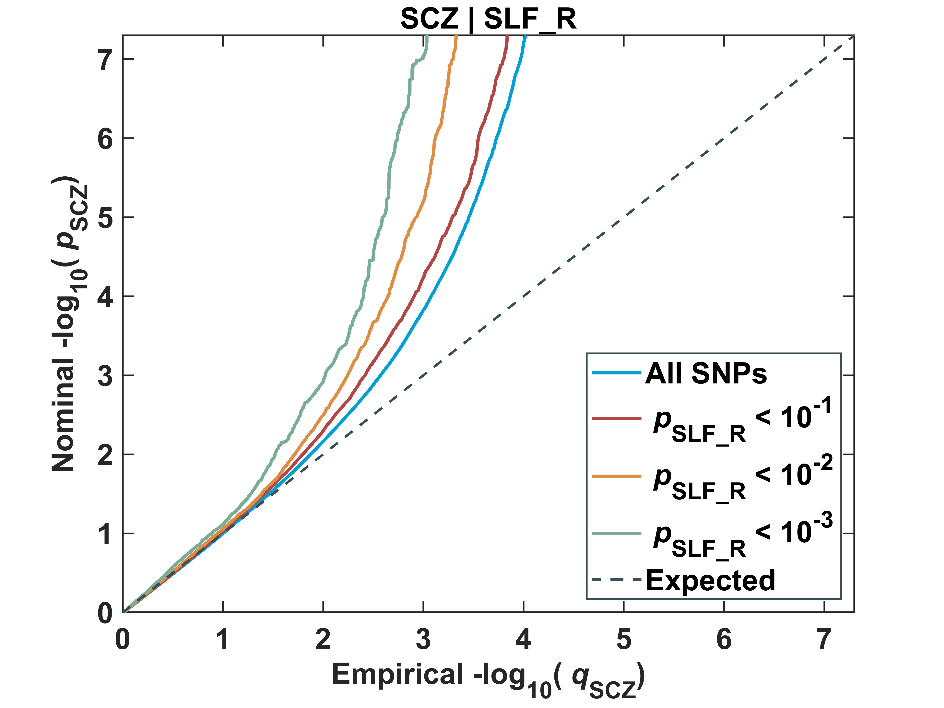

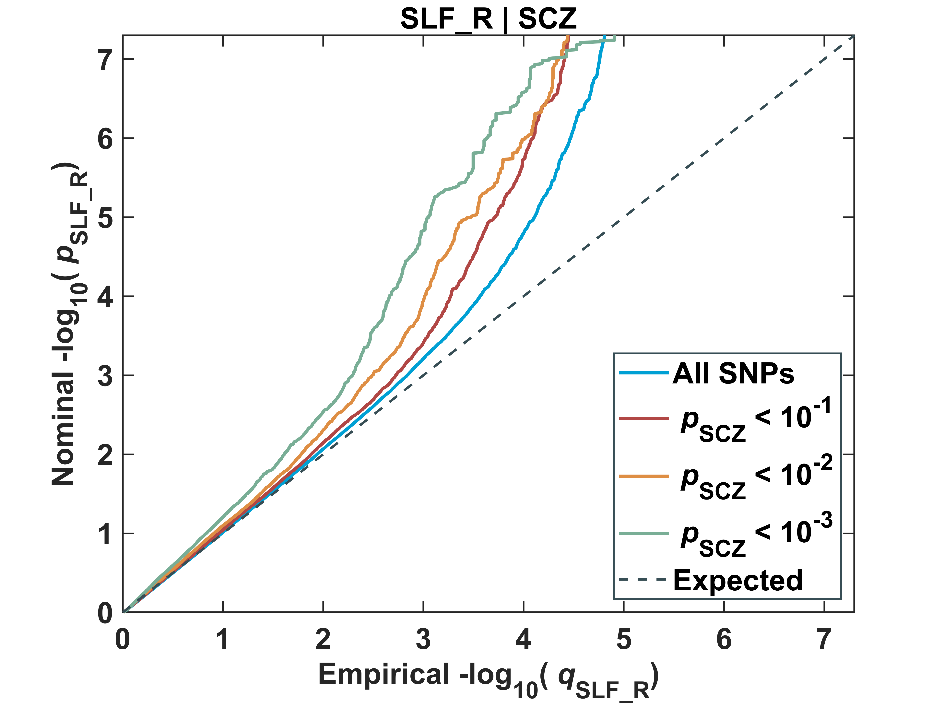

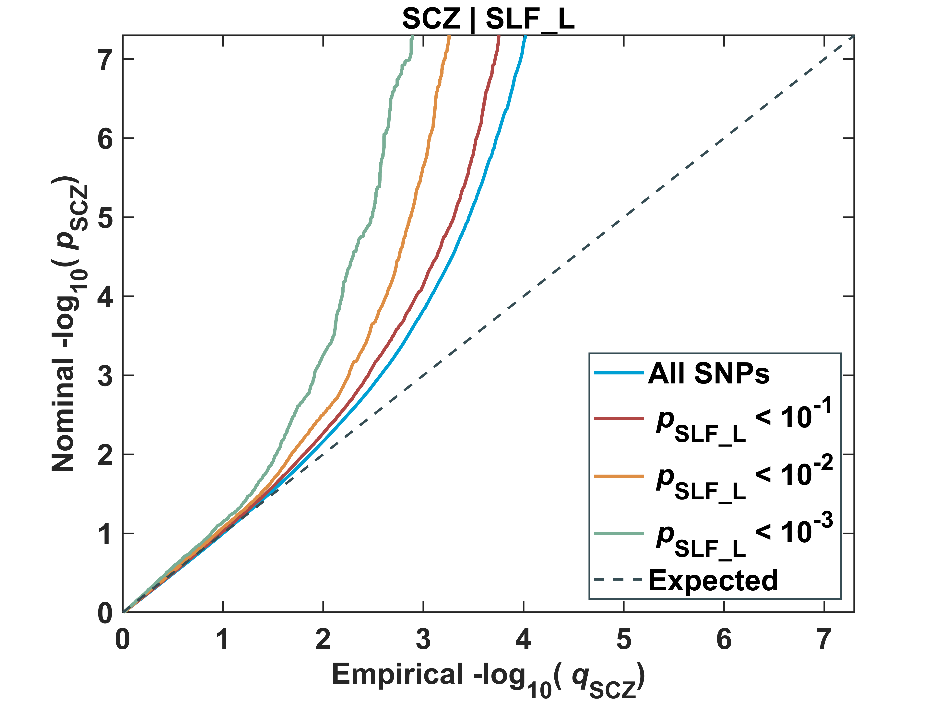

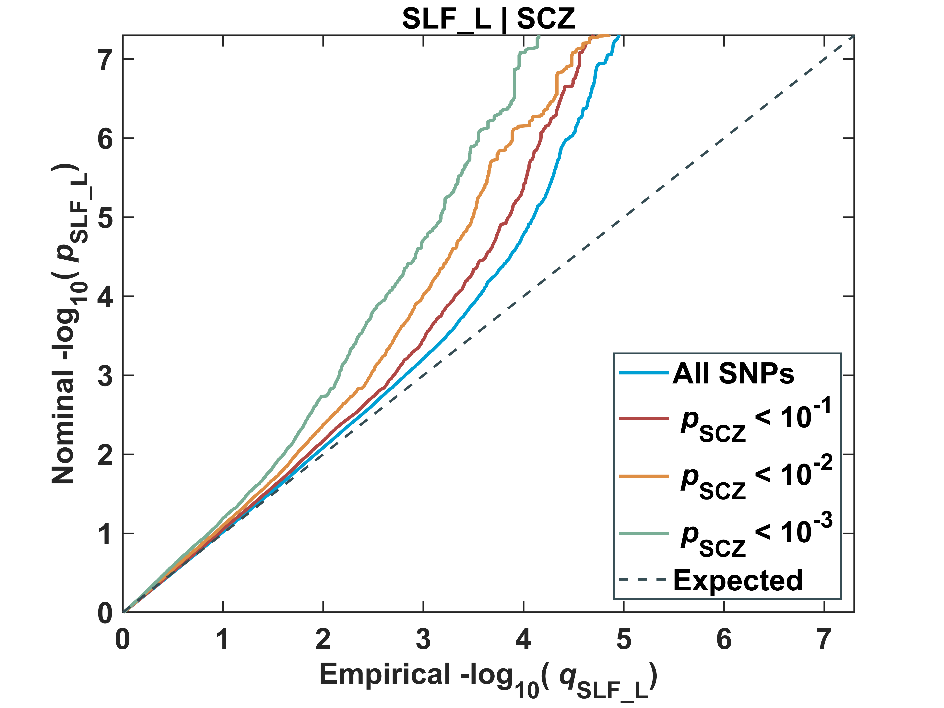

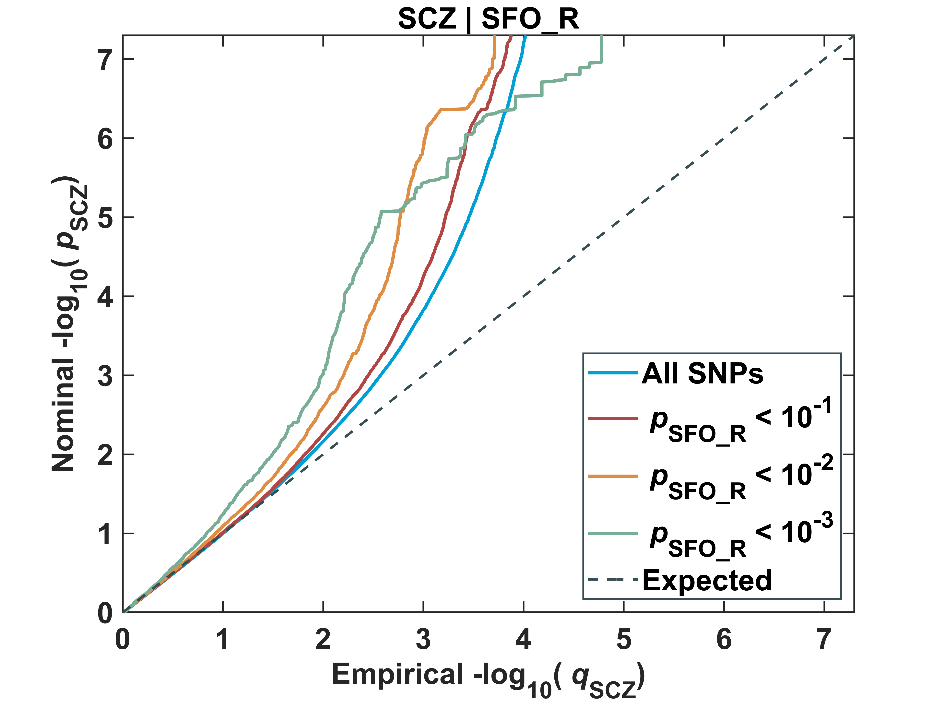

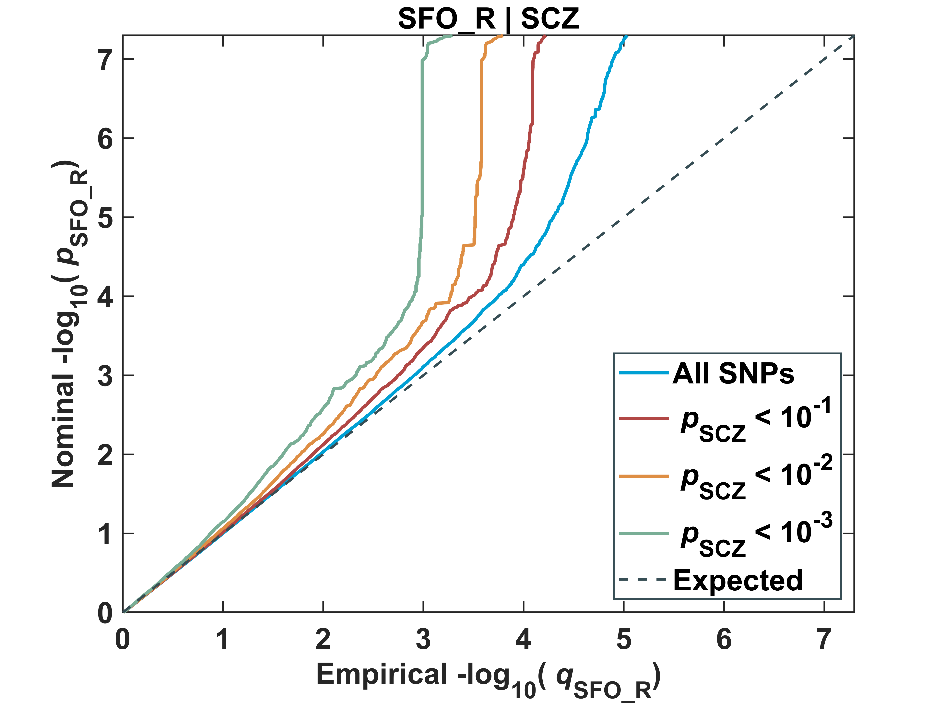

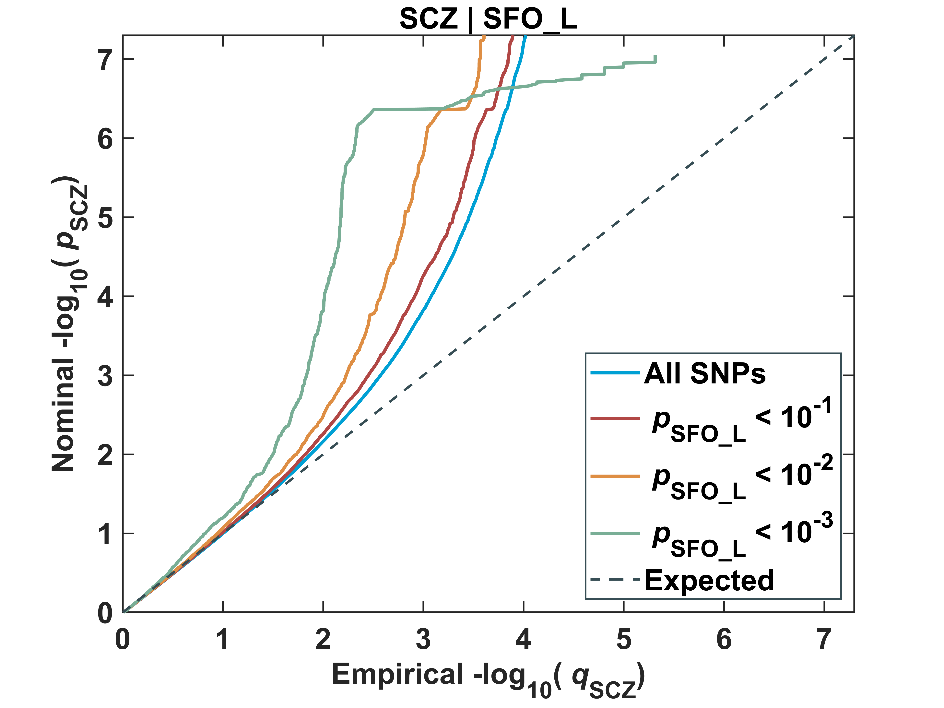

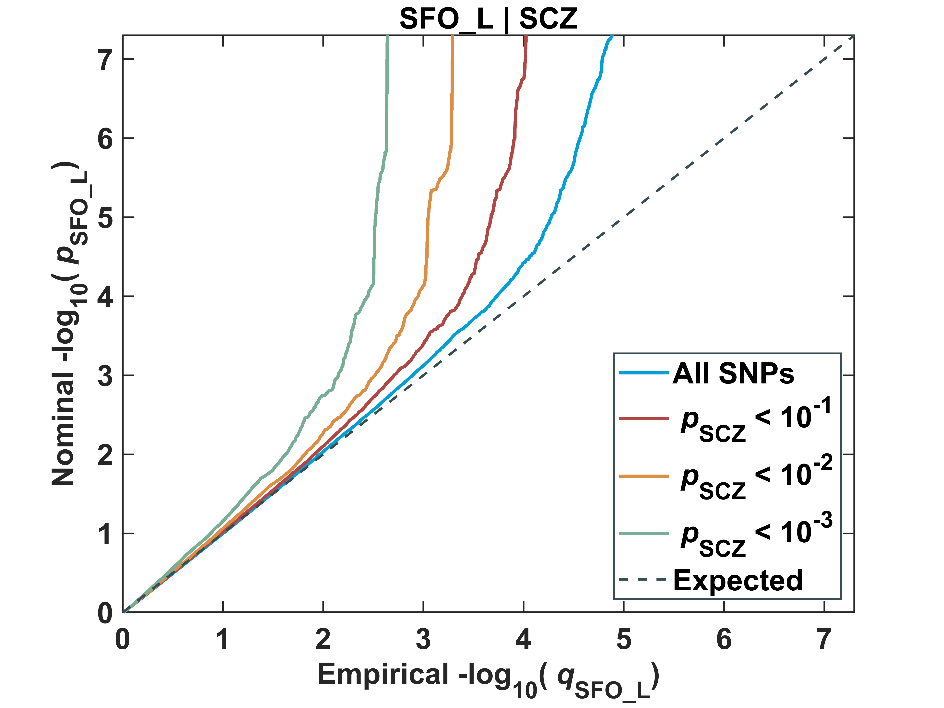

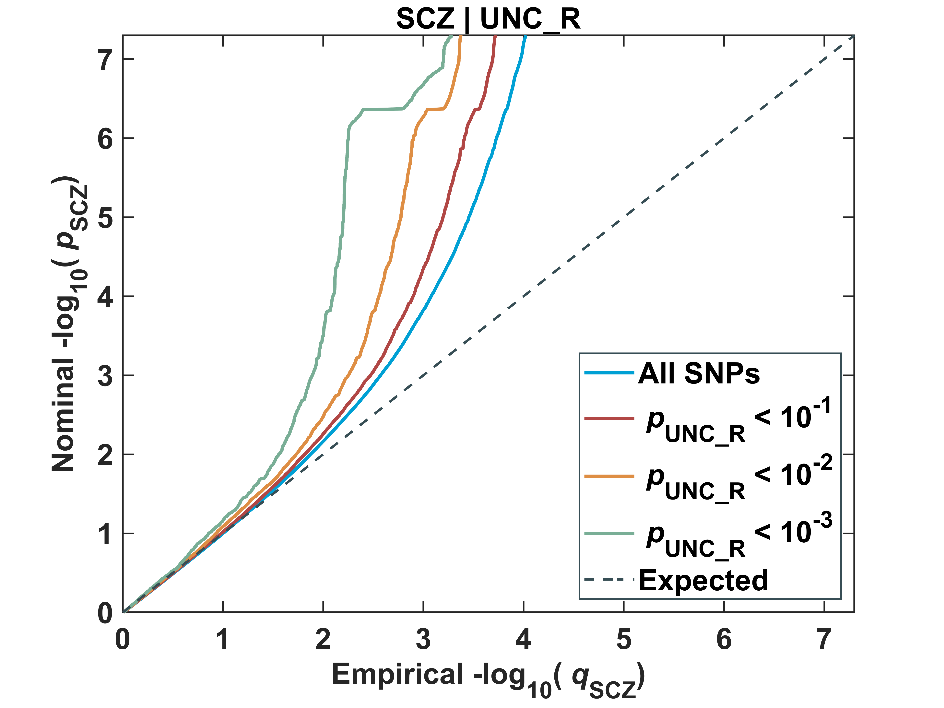

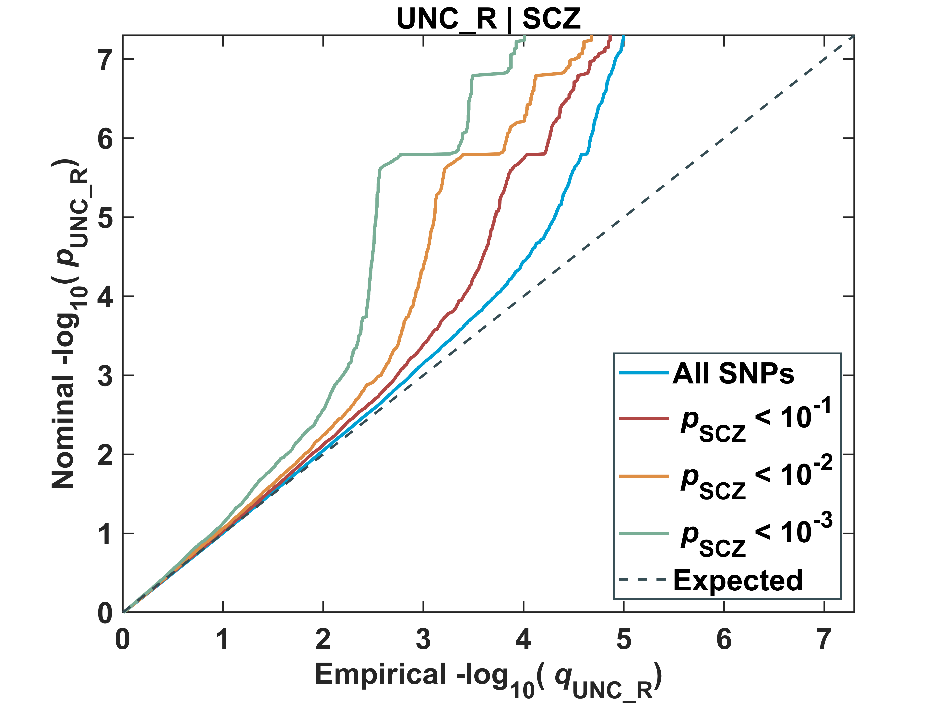

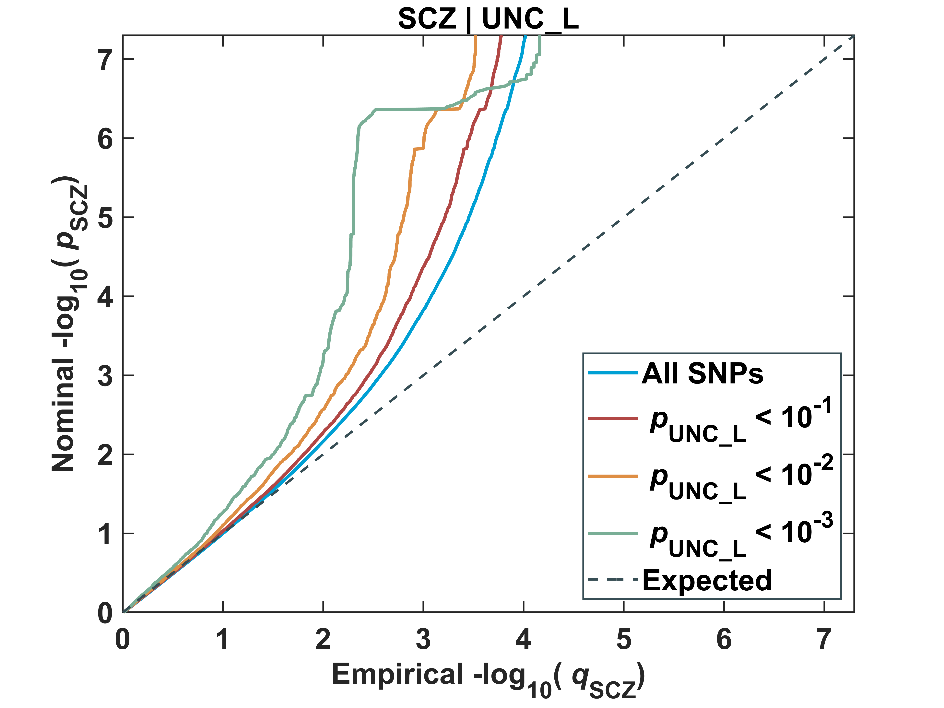

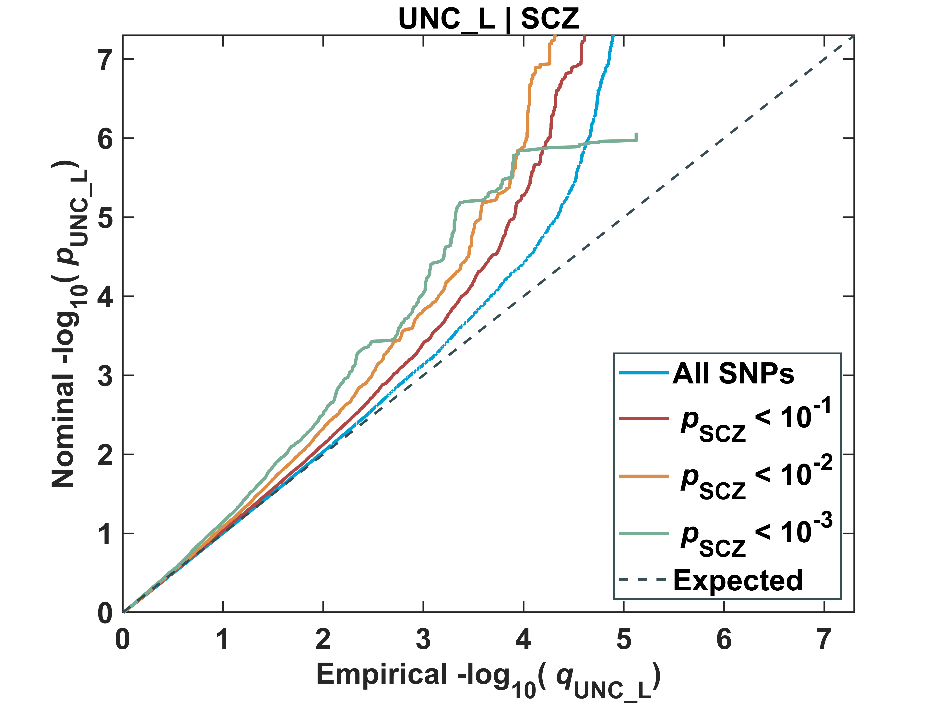

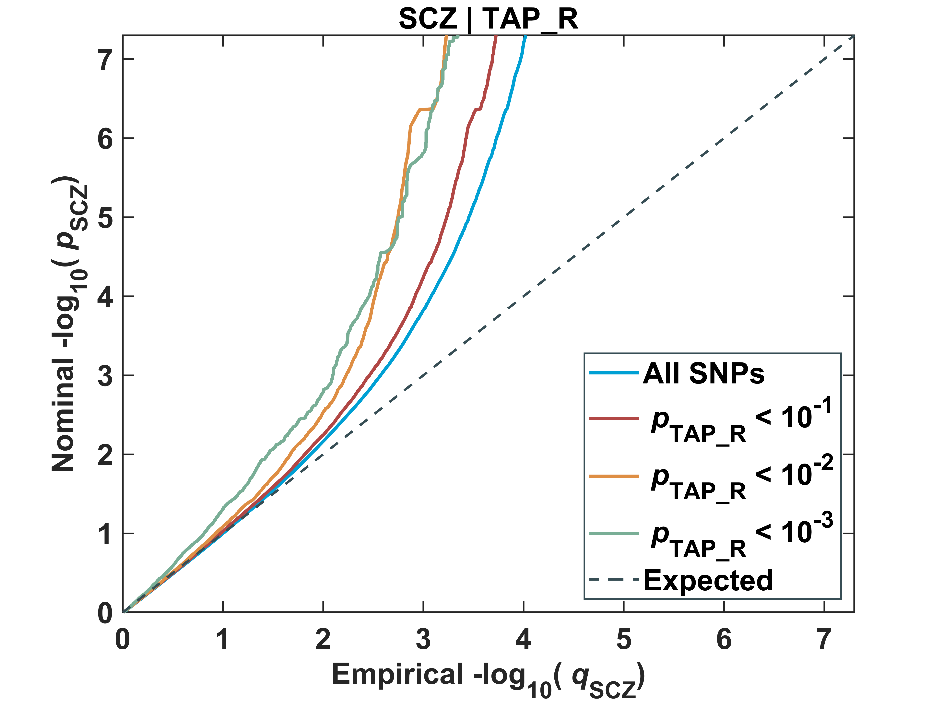

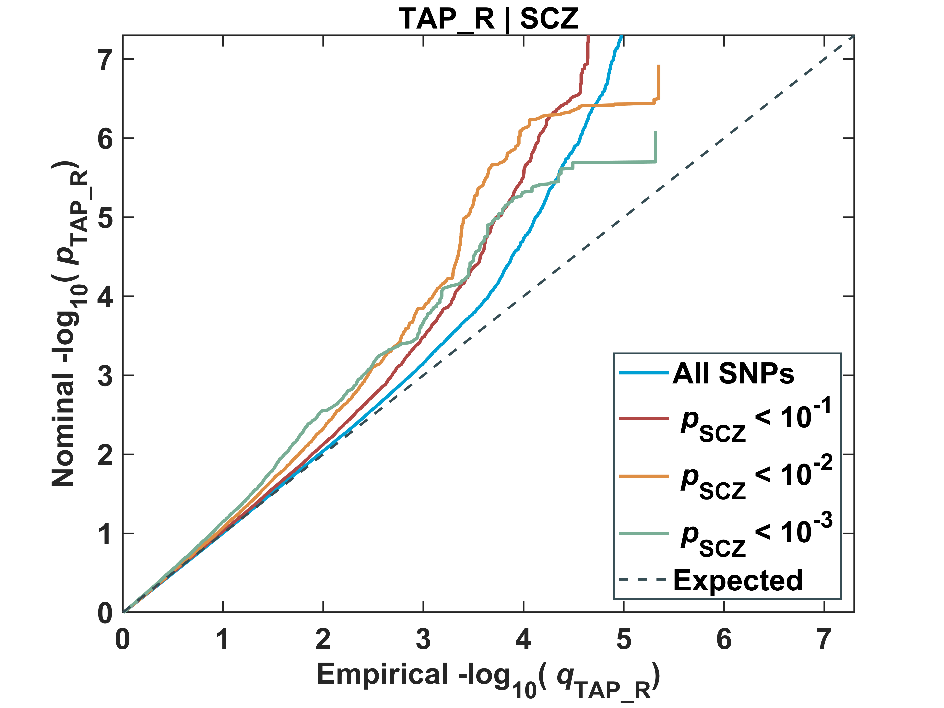

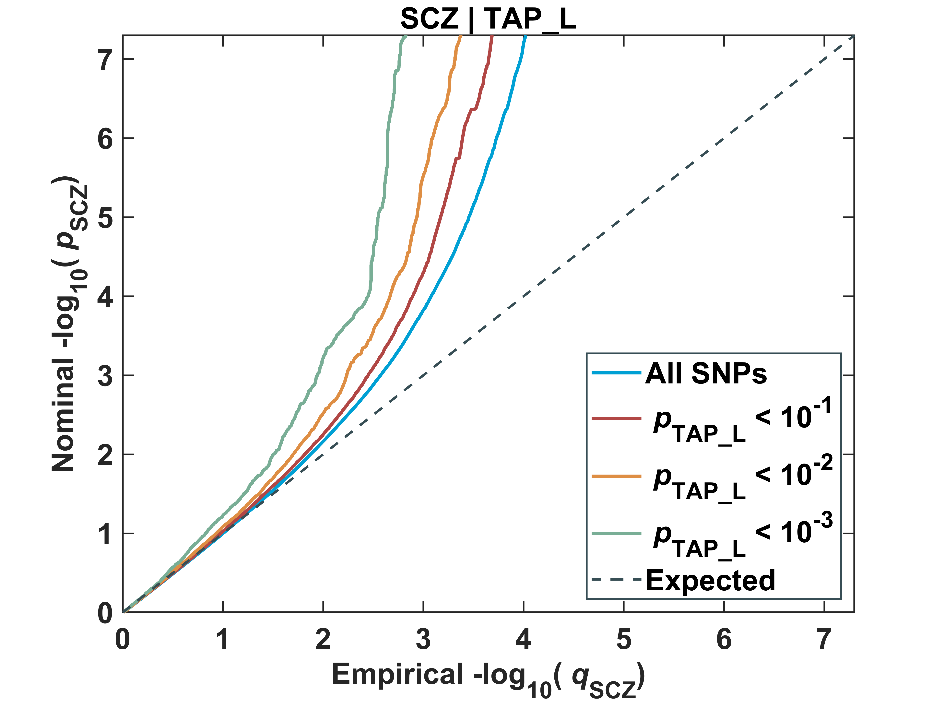

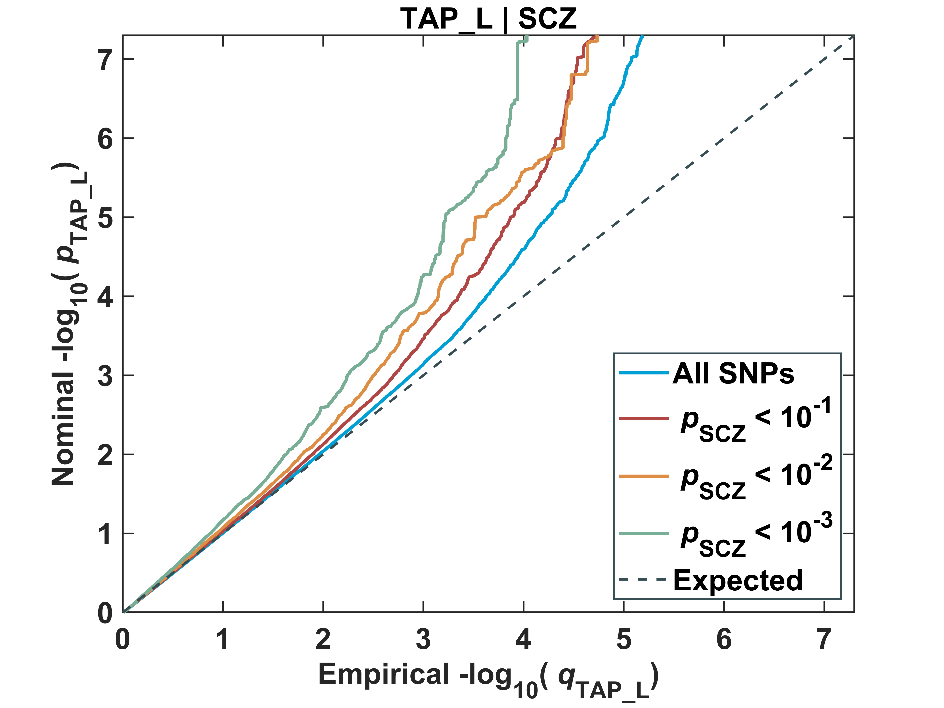


## **Fig. S1. Polygenic overlap between schizophrenia and white matter fractional anisotropy.**

The conditional *Q-Q* plots display nominal versus empirical -log_10_-transformed *p*-values for the primary phenotype across all SNPs or SNPs associated with the secondary phenotype at different significant levels (*p* < 0.1, *p* < 0.01 and *p* < 0.001). First, schizophrenia as the primary phenotype and white matter fractional anisotropy as the secondary phenotype, and then reversing the roles of the primary and secondary phenotypes.

Abbreviations: ACR, anterior corona radiata; ALIC, anterior limb of internal capsule; BCC, body of corpus callosum; CCG, cingulum cingulate gyrus; CH, cingulum hippocampus; CP, cerebral peduncle; CST, corticospinal tract; EC, external capsule; FX, fornix; FXC+ST, fornix cres+stria terminalis; GCC, genu of corpus callosum; ICP, inferior cerebellar peduncle; L, left; MCP, middle cerebellar peduncle; ML, medial lemniscus; PCR, posterior corona radiata; PCT, pontine crossing tract; PLIC, posterior limb of internal capsule; PTR, posterior thalamic radiation; R, right; RLIC, retrolenticular part of internal capsule; SCC, splenium of corpus callosum; SCP, superior cerebellar peduncle; SCR, superior corona radiata; SCZ, schizophrenia; SFO, superior fronto-occipital fasciculus; SLF, superior longitudinal fasciculus; SS, sagittal stratum; TAP, tapetum; UNC, uncinate fasciculus.


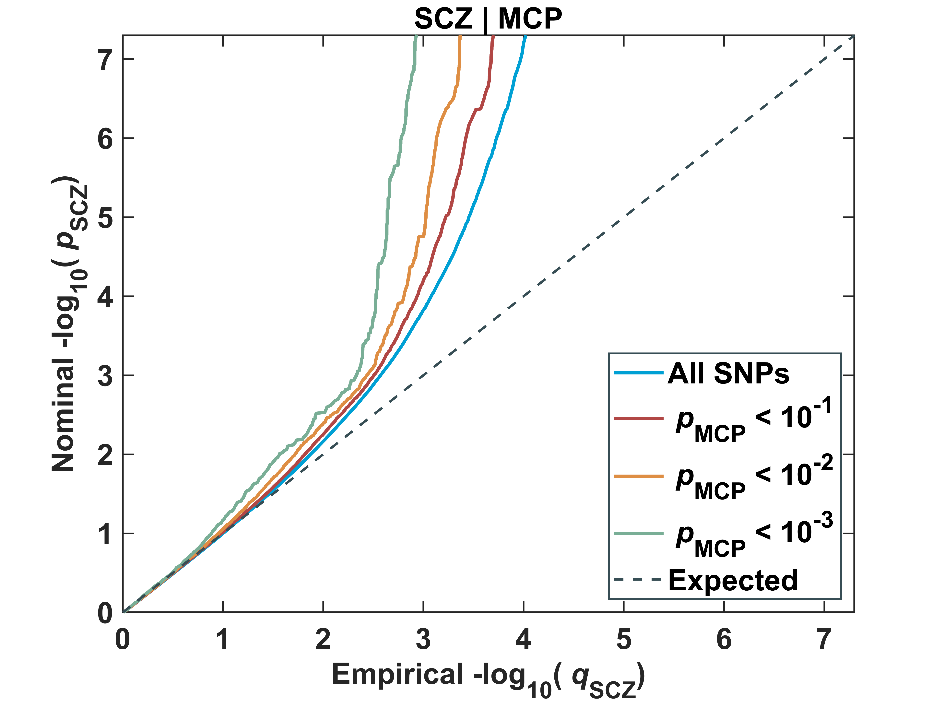

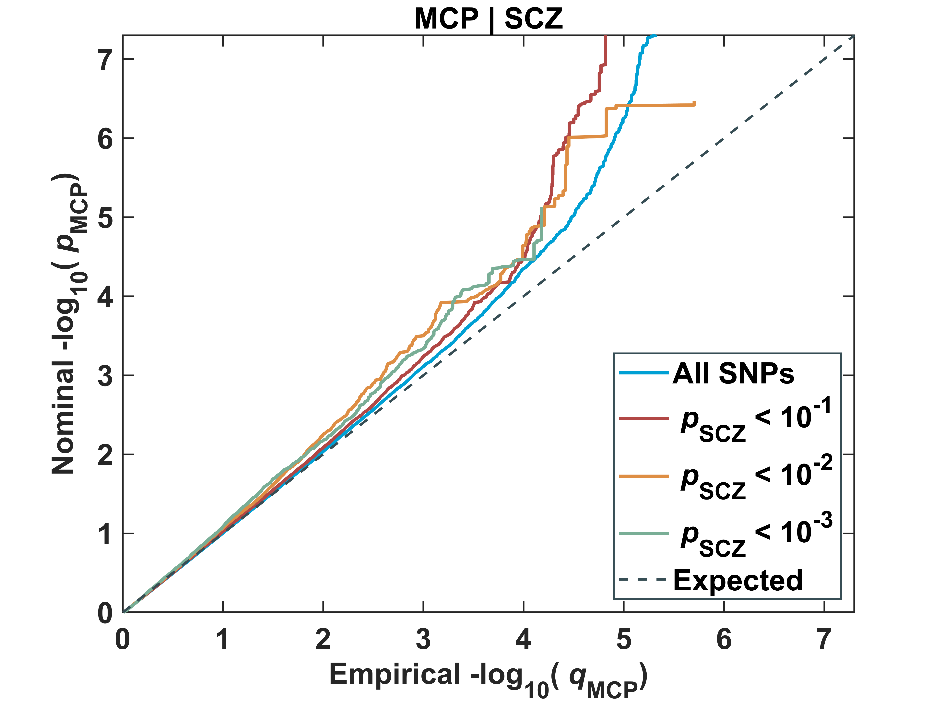

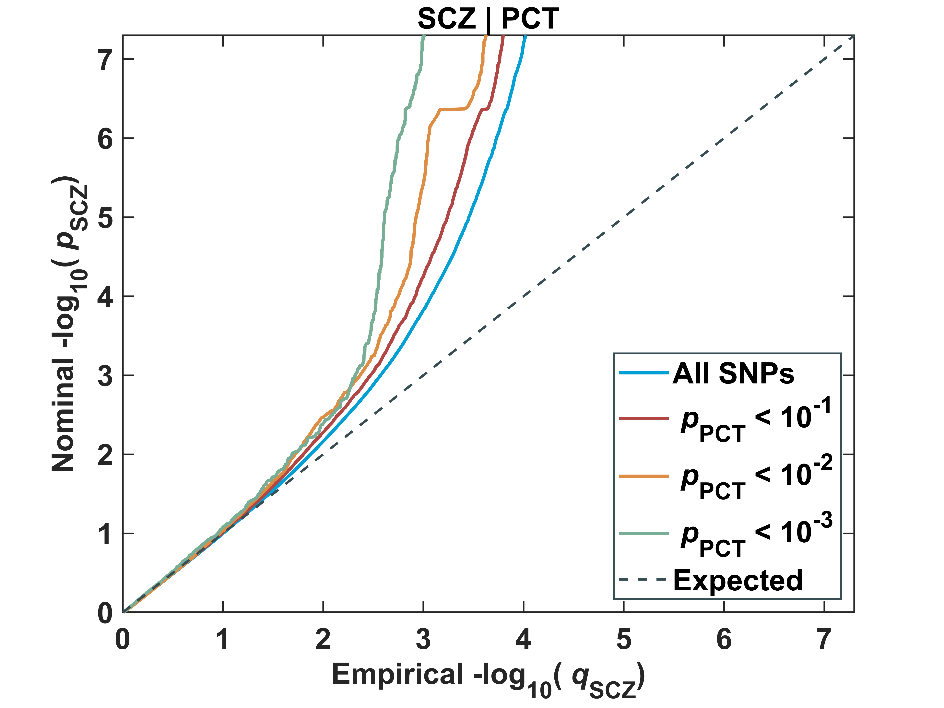

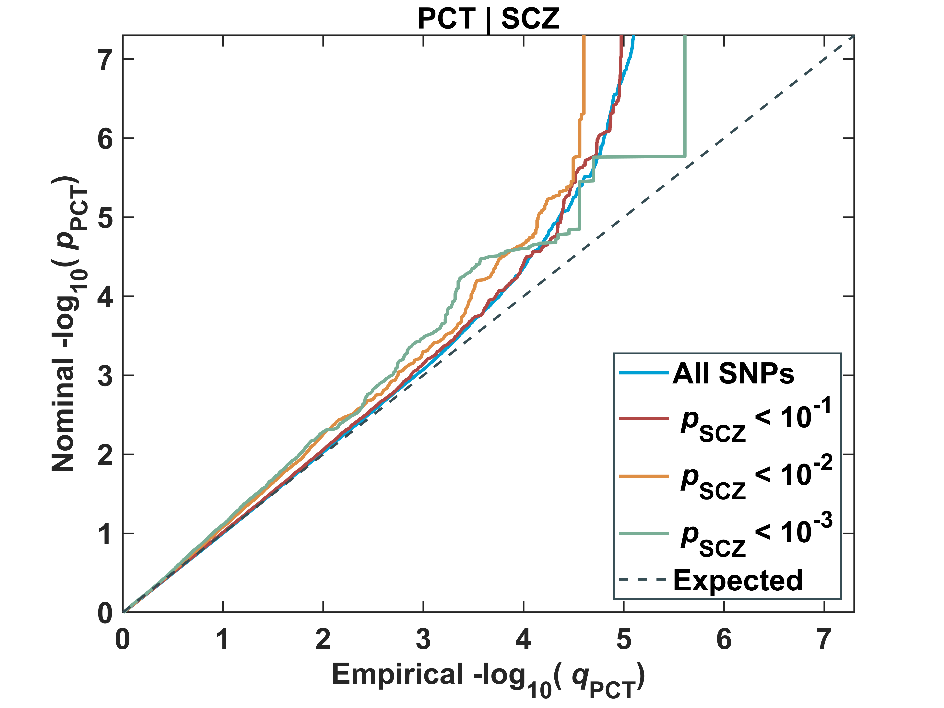


## **Fig. S2. Polygenic overlap between schizophrenia and white matter mean diffusivity.**

The conditional *Q-Q* plots display nominal versus empirical -log_10_-transformed *p*-values for the primary phenotype across all SNPs or SNPs associated with the secondary phenotype at different significant levels (*p* < 0.1, *p* < 0.01 and *p* < 0.001). First, schizophrenia as the primary phenotype and white matter mean diffusivity as the secondary phenotype, and then reversing the roles of the primary and secondary phenotypes.

Abbreviations: ACR, anterior corona radiata; ALIC, anterior limb of internal capsule; BCC, body of corpus callosum; CCG, cingulum cingulate gyrus; CH, cingulum hippocampus; CP, cerebral peduncle; CST, corticospinal tract; EC, external capsule; FX, fornix; FXC+ST, fornix cres+stria terminalis; GCC, genu of corpus callosum; ICP, inferior cerebellar peduncle; L, left; MCP, middle cerebellar peduncle; ML, medial lemniscus; PCR, posterior corona radiata; PCT, pontine crossing tract; PLIC, posterior limb of internal capsule; PTR, posterior thalamic radiation; R, right; RLIC, retrolenticular part of internal capsule; SCC, splenium of corpus callosum; SCP, superior cerebellar peduncle; SCR, superior corona radiata; SCZ, schizophrenia; SFO, superior fronto-occipital fasciculus; SLF, superior longitudinal fasciculus; SS, sagittal stratum; TAP, tapetum; UNC, uncinate fasciculus.

## **Fig. S3. Polygenic overlap between schizophrenia and white matter λ1.**

The conditional *Q-Q* plots display nominal versus empirical -log_10_-transformed *p*-values for the primary phenotype across all SNPs or SNPs associated with the secondary phenotype at different significant levels (*p* < 0.1, *p* < 0.01 and *p* < 0.001). First, schizophrenia as the primary phenotype and white matter λ1 as the secondary phenotype, and then reversing the roles of the primary and secondary phenotypes.

Abbreviations: ACR, anterior corona radiata; ALIC, anterior limb of internal capsule; BCC, body of corpus callosum; CCG, cingulum cingulate gyrus; CH, cingulum hippocampus; CP, cerebral peduncle; CST, corticospinal tract; EC, external capsule; FX, fornix; FXC+ST, fornix cres+stria terminalis; GCC, genu of corpus callosum; ICP, inferior cerebellar peduncle; L, left; MCP, middle cerebellar peduncle; ML, medial lemniscus; PCR, posterior corona radiata; PCT, pontine crossing tract; PLIC, posterior limb of internal capsule; PTR, posterior thalamic radiation; R, right; RLIC, retrolenticular part of internal capsule; SCC, splenium of corpus callosum; SCP, superior cerebellar peduncle; SCR, superior corona radiata; SCZ, schizophrenia; SFO, superior fronto-occipital fasciculus; SLF, superior longitudinal fasciculus; SS, sagittal stratum; TAP, tapetum; UNC, uncinate fasciculus.

## **Fig. S4. Polygenic overlap between schizophrenia and white matter λ2.**

The conditional *Q-Q* plots display nominal versus empirical -log_10_-transformed *p*-values for the primary phenotype across all SNPs or SNPs associated with the secondary phenotype at different significant levels (*p* < 0.1, *p* < 0.01 and *p* < 0.001). First, schizophrenia as the primary phenotype and white matter λ2 as the secondary phenotype, and then reversing the roles of the primary and secondary phenotypes.

Abbreviations: ACR, anterior corona radiata; ALIC, anterior limb of internal capsule; BCC, body of corpus callosum; CCG, cingulum cingulate gyrus; CH, cingulum hippocampus; CP, cerebral peduncle; CST, corticospinal tract; EC, external capsule; FX, fornix; FXC+ST, fornix cres+stria terminalis; GCC, genu of corpus callosum; ICP, inferior cerebellar peduncle; L, left; MCP, middle cerebellar peduncle; ML, medial lemniscus; PCR, posterior corona radiata; PCT, pontine crossing tract; PLIC, posterior limb of internal capsule; PTR, posterior thalamic radiation; R, right; RLIC, retrolenticular part of internal capsule; SCC, splenium of corpus callosum; SCP, superior cerebellar peduncle; SCR, superior corona radiata; SCZ, schizophrenia; SFO, superior fronto-occipital fasciculus; SLF, superior longitudinal fasciculus; SS, sagittal stratum; TAP, tapetum; UNC, uncinate fasciculus.

## **Fig. S5. Polygenic overlap between schizophrenia and white matter λ3.**

The conditional *Q-Q* plots display nominal versus empirical -log_10_-transformed *p*-values for the primary phenotype across all SNPs or SNPs associated with the secondary phenotype at different significant levels (*p* < 0.1, *p* < 0.01 and *p* < 0.001). First, schizophrenia as the primary phenotype and white matter λ3 as the secondary phenotype, and then reversing the roles of the primary and secondary phenotypes.

Abbreviations: ACR, anterior corona radiata; ALIC, anterior limb of internal capsule; BCC, body of corpus callosum; CCG, cingulum cingulate gyrus; CH, cingulum hippocampus; CP, cerebral peduncle; CST, corticospinal tract; EC, external capsule; FX, fornix; FXC+ST, fornix cres+stria terminalis; GCC, genu of corpus callosum; ICP, inferior cerebellar peduncle; L, left; MCP, middle cerebellar peduncle; ML, medial lemniscus; PCR, posterior corona radiata; PCT, pontine crossing tract; PLIC, posterior limb of internal capsule; PTR, posterior thalamic radiation; R, right; RLIC, retrolenticular part of internal capsule; SCC, splenium of corpus callosum; SCP, superior cerebellar peduncle; SCR, superior corona radiata; SCZ, schizophrenia; SFO, superior fronto-occipital fasciculus; SLF, superior longitudinal fasciculus; SS, sagittal stratum; TAP, tapetum; UNC, uncinate fasciculus.

## **Fig. S6. The Manhattan plot of SNP shared between schizophrenia and white matter** **microstructure.**

The *x*-axis represents the chromosome position, whereas the *y*-axis denotes the -log_10_-transformed conjFDR values. The dotted horizontal line indicates the threshold for significant association. Each dot corresponds to a SNP, with those bordered in black signifying lead SNPs.

Abbreviations: FA, fractional anisotropy; MD, mean diffusivity.

## **Fig. S7. Ideogram of genomic genetic variants shared between schizophrenia and white matter fractional anisotropy.**

Colors represent the 48 different white matter tracts. Each point corresponds to a lead SNP jointly associated with schizophrenia and white matter fractional anisotropy.

## **Fig. S8. Ideogram of genomic genetic variants shared between schizophrenia and white matter mean diffusivity.**

Colors represent the 48 different white matter tracts. Each point corresponds to a lead SNP jointly associated with schizophrenia and white matter mean diffusivity.

## **Fig. S9. Ideogram of genomic genetic variants shared between schizophrenia and white matter λ1.**

Colors represent the 48 different white matter tracts. Each point corresponds to a lead SNP jointly associated with schizophrenia and white matter λ1.

## **Fig. S10. Ideogram of genomic genetic variants shared between schizophrenia and white matter λ2.**

Colors represent the 48 different white matter tracts. Each point corresponds to a lead SNP jointly associated with schizophrenia and white matter λ2.

## **Fig. S11. Ideogram of genomic genetic variants shared between schizophrenia and white matter λ3.**

Colors represent the 48 different white matter tracts. Each point corresponds to a lead SNP jointly associated with schizophrenia and white matter λ3.

## **Fig. S12. Hemispheric distribution of distinct loci shared between schizophrenia and fractional anisotropy in 21 pairs of white matter tracts.**

The images illustrate the left hemisphere-specific loci (red), the right hemisphere-specific loci (blue) and loci overlapping between the two hemispheric (green). The dots on the diagram represent the top lead SNP within the distinct locus.

## **Fig. S13. Hemispheric distribution of distinct loci shared between schizophrenia and mean diffusivity in 21 pairs of white matter tracts.**

The images illustrate the left hemisphere-specific loci (red), the right hemisphere-specific loci (blue) and loci overlapping between the two hemispheric (green). The dots on the diagram represent the top lead SNP within the distinct locus.

## **Fig. S14. Hemispheric distribution of distinct loci shared between schizophrenia and λ1 in 21 pairs of white matter tracts.**

The images illustrate the left hemisphere-specific loci (red), the right hemisphere-specific loci (blue) and loci overlapping between the two hemispheric (green). The dots on the diagram represent the top lead SNP within the distinct locus.

## **Fig. S15. Hemispheric distribution of distinct loci shared between schizophrenia and λ2 in 21 pairs of white matter tracts.**

The images illustrate the left hemisphere-specific loci (red), the right hemisphere-specific loci (blue) and loci overlapping between the two hemispheric (green). The dots on the diagram represent the top lead SNP within the distinct locus.

## **Fig. S16. Hemispheric distribution of distinct loci shared between schizophrenia and λ3 in 21 pairs of white matter tracts.**

The images illustrate the left hemisphere-specific loci (red), the right hemisphere-specific loci (blue) and loci overlapping between the two hemispheric (green). The dots on the diagram represent the top lead SNP within the distinct locus.

## **Fig. S17. Proportions of left-specific, right-specific, and overlapping loci across 21 tract pairs under different DTI parameters.**

The *x*-axis represents different white matter tracts and the *y*-axis indicates the proportion of each locus type relative to the total number of distinct loci. Red dashed, blue dashed, and green solid lines represent left-specific, right-specific, and overlapping loci, respectively.

Abbreviations: ACR, anterior corona radiata; ALIC, anterior limb of internal capsule; CCG, cingulum cingulate gyrus; CH, cingulum hippocampus; CP, cerebral peduncle; CST, corticospinal tract; EC, external capsule; FA, fractional anisotropy; FXC+ST, fornix cres+stria terminalis; ICP, inferior cerebellar peduncle; MD, mean diffusivity; ML, medial lemniscus; PCR, posterior corona radiata; PLIC, posterior limb of internal capsule; PTR, posterior thalamic radiation; RLIC, retrolenticular part of internal capsule; SCP, superior cerebellar peduncle; SCR, superior corona radiata; SFO, superior fronto-occipital fasciculus; SLF, superior longitudinal fasciculus; SS, sagittal stratum; TAP, tapetum; UNC, uncinate fasciculus.

## **Fig. S18. Venn diagram of overlapping genes shared between schizophrenia and five diffusion tensor imaging parameters.**

Abbreviations: FA, fractional anisotropy; FDR, false discovery rate; MD, mean diffusivity.

## **Fig. S19. Tissue- and cell-type specificity of genes shared between schizophrenia and white matter microstructure.**

**A.** The top 20 enriched tissues. **B.** The top 20 enriched general cell types. Each dot corresponds to a tissue or cell type. The *x*-axis represents tissues or cell types, while the *y*-axis shows the -log_10_-transformed *p*-values. The red dashed line indicates the Bonferroni-corrected significance threshold (*p* < 3.69E-5), while the grey solid line denotes the nominal significance threshold (*p* < 1E-3).

Abbreviation: TS, tissue-specificity.

## **Fig. S20. Genetic correlations between schizophrenia and white matter microstructure.**

The color of the dots indicates the magnitude of the correlation coefficient, while their size represents the -log_10_-transformed *p*-value of the genetic correlation. Dots with black borders denote nominal *p*-value < 0.05.

Abbreviations: ACR, anterior corona radiata; ALIC, anterior limb of internal capsule; BCC, body of corpus callosum; CCG, cingulum cingulate gyrus; CP, cerebral peduncle; FA, fractional anisotropy; FXC+ST, fornix cres+stria terminalis; GCC, genu of corpus callosum; ICP, inferior cerebellar peduncle; L, left; MCP, middle cerebellar peduncle; MD, mean diffusivity; PCR, posterior corona radiata; PTR, posterior thalamic radiation; R, right; RLIC, retrolenticular part of internal capsule; SCC, splenium of corpus callosum; SCR, superior corona radiata; SFO, superior fronto-occipital fasciculus; SLF, superior longitudinal fasciculus; SS, sagittal stratum; TAP, tapetum; UNC, uncinate fasciculus.

## **Fig. S21. The allelic effect direction test of lead SNPs shared between schizophrenia and white matter microstructure.**

*N_SNP_* represents the number of shared lead SNPs available in the independent dataset, and *N_CON_* represents the number of lead SNPs that exhibit a consistent effect in both the discovery and independent datasets. The *x*-axis is *N_CON_*/*N_SNP_*, reflecting the proportion of consistency. The color represents the -log_10_-transformed FDR adjusted *p*-values.

Abbreviations: FA, fractional anisotropy; FDR, false discovery rate; MD, mean diffusivity.
